# A risk index tool to minimize the risk of nitrogen loss from land to water

**DOI:** 10.1002/jeq2.20660

**Published:** 2024-12-12

**Authors:** R. W. McDowell, V. O. Snow, R. Tamepo, L. Lilburne, R. Cichota, K. Muraoka, E. Soal

**Affiliations:** ^1^ Faculty of Agriculture and Life Sciences Lincoln University Lincoln New Zealand; ^2^ AgResearch, Lincoln Science Centre Lincoln New Zealand; ^3^ Scion Research Rotorua New Zealand; ^4^ Manaaki Whenua Landcare Research Lincoln New Zealand; ^5^ Plant and Food Research Lincoln New Zealand; ^6^ Ministry for the Environment Wellington New Zealand; ^7^ ES Water Policy Oamaru New Zealand

## Abstract

Simple models can help reduce nitrogen (N) loss from land and protect water quality. However, the complexity of primary production systems may impair the accuracy of simple models. A tool was developed that assessed the risk of N loss as the product of N source inputs and relative transport by leaching and runoff. A dynamic process‐based model was used to estimate the long‐term monthly N loss risk by leaching and runoff in response to the interaction of static biophysical factors like soil type, slope, and long‐term climate. Source inputs included dung and urine (from livestock), fertilizer, crop residues, and soil erosion. Estimates of the rank of N loss risk were related (*r* ^2^ = 0.69, *p *< 0.001) to 96 observations of N loss (kg ha^−1^ year^−1^) across nine land uses; all but two of the observations fell within 95% prediction intervals. Across land uses, leaching accounted for 84% of N loss risk. Additional observations are needed to determine if N loss risk is representative of short‐rotation vegetables and to account for potential lag times between calculated and measured losses. The good performance of the tool suggests that when displayed spatially, the tool can be used to target high‐risk areas with actions to reduce the risk of N loss and the likelihood of water quality impairment.

## Core Ideas

- Risk of nitrogen (N) loss varies by the availability of N sources and transport by runoff or leaching.
- Underpinning N transport risk was modeled nationally by climate, soil type (and slope).
- Combining N sources (e.g., fertilizer and excreta) with transport maps risk of N loss across farms.
- Risk of N loss was validated against observed N losses for multiple farm types.
- Implementing mitigation practices by risk is an efficient way to meet water quality policy.

**Abbreviation**
APSIM: Agricultural Production Systems Simulator

## 1 INTRODUCTION

Nitrogen (N) loss from land can harm water quality (Larned et al., 2020). Several factors affect N losses from topsoil. These factors include the rate and form of N input to the soil (Beukes et al., 2020), processes that cause transformation of N between inorganic, organic, dissolved (<0.45 µm), and particulate forms (Dungait et al., 2012; Wang et al., 2019), removal of N from the soil through plant uptake (Malcolm et al., 2022), and soil and climatic factors that affect N transport via surface or subsurface pathways (Christensen et al., 2019; Christie et al., 2020). Because these factors interact, we employ models to capture their complexity and quantify N loss (Hajati et al., 2020).

Various models are used to quantify N loss to water, driven by the need to match N loss predictions with effective management strategies (Follett, 1995; D. P. Holzworth et al., 2014; Shaffer et al., 1991; Singh et al., 2020; Vieritz et al., 2019). Models vary from simple scorecards that estimate N loss based on long‐term management (Fonterra Co‐op Ltd., 2020), to models that quantify N loss from topsoil by considering daily climate conditions and management decisions (D. Holzworth et al., 2018). Simpler models tend to be easier for people to interpret and recommend actions to mitigate N loss and improve water quality. However, models must also be accurate to maximize the likelihood that actions will be successful. For instance, if a scorecard only considered management without considering soil type, it may erroneously suggest similar N loss for two properties using the same management but different soil characteristics (e.g., a stony, shallow soil and a deep, fine‐textured soil). Hence, there is often a trade‐off between simplicity, which aims to make the model accessible and easy to understand, and complexity, which may be necessary to capture biophysical and management interactions.

Risk indices have been used for at least 20 years in the United States and more recently in other jurisdictions as a tool to quantify the risk of contaminant loss (Bechmann et al., 2005; Hughes et al., 2005; Lemunyon & Gilbert, 1993; Ribey & O'Halloran, 2016; Ulén et al., 2011). Most risk indices have been developed to manage and mitigate the loss of phosphorus (P). These P indices have commonly assessed risk on a field‐by‐field basis (or within fields), presenting the risk in a map to facilitate targeting mitigation actions to high‐risk areas (Sharpley et al., 2008). Users appreciate this approach due to its simplicity and transparency, fostering greater trust compared to complex models that may appear opaque to users (Sharpley et al., 2003).

There are also examples of N risk indices. However, these focus on the risk of nitrate loss from arable topsoils to subsurface flow (commonly termed leaching) that may enter artificial drainage networks or shallow to deep groundwater (Delgado et al., 2006; Drewry et al., 2011; Figueroa‐Viramontes et al., 2016). This focus on nitrate is reasonable in such conditions, because nitrate is the dominant form of N loss (McDowell et al., 2008). Nitrate can also dominate (contributing over 80%) total N loss from intensively grazed dairy farms on flat land that is well‐ to moderately‐well‐drained land (Christensen et al., 2019; Monaghan et al., 2016). However, a significant proportion of N loss can occur in other forms and through surface runoff. For instance, Burkitt (2014) found that runoff losses of N could account for as little as 25% (typically less than 5 kg N ha^−1^ year^−1^) of total N losses from flat land with well‐drained soils and up to 50% (23 kg N ha^−1^ year^−1^) on sloping land with high rainfall (e.g., >2000 mm year^−1^) or flat land subjected to flood irrigation. Consequently, any N loss index should consider N loss via multiple pathways at a time frame that is relevant to management.

It is well established that to cost‐effectively improve water quality, mitigation actions need to be targeted correctly and implemented at the appropriate time (Van Grinsven et al., 2016; Vinten et al., 2017). We see an opportunity to produce a hybrid tool that combines accuracy of modeling with the transparency and action‐oriented focus of an index. Modeling can capture the inherent risk of leaching or runoff based on unchanging biophysical factors like soil type and slope. When combined with long‐term climate data, the daily risk of leaching can be produced. Once aggregated to a monthly basis, these risks align with the recording of source inputs like fertilizer application or livestock numbers and can be combined with transport risks to evaluate the overall risk of N loss. We aimed to show how such an index could be constructed, tested, and applied to assess and manage the risk of N loss and help achieve better water quality outcomes.

**Core Ideas**

- Risk of nitrogen (N) loss varies by the availability of N sources and transport by runoff or leaching.
- Underpinning N transport risk was modeled nationally by climate, soil type (and slope).
- Combining N sources (e.g., fertilizer and excreta) with transport maps risk of N loss across farms.
- Risk of N loss was validated against observed N losses for multiple farm types.
- Implementing mitigation practices by risk is an efficient way to meet water quality policy.

## 2 TOOL STRUCTURE

**FIGURE 1:**
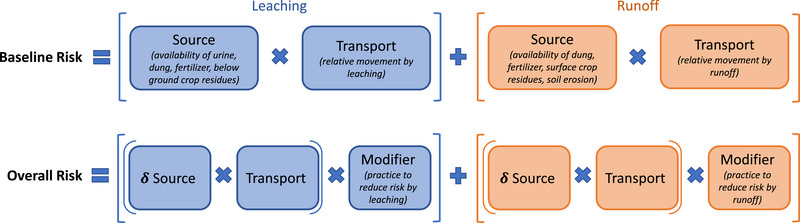
Conceptual outline of the N loss risk index at a block scale.

The overall structure of the tool is shown in Figure 1. The tool is free and available at: https://stage.mfe‐rit.webtools.ag/login. The risk of N loss is assessed as the product of the availability of N for loss (source) and the likelihood of loss (transport). This is termed *baseline risk*, which can be altered by an additional module that *modifies risk* of N loss based on management practices (Figure 1).

Transport is assessed via the open‐source Agricultural Production Systems Simulator model (APSIM; www.apsim.info) (D. Holzworth et al., 2018). This model has been extensively validated across a wide range of environments and land uses (Archontoulis et al., 2014; Cichota et al., 2018, 2010; Hoffmann et al., 2018; V. O. Snow et al., 1999; Vogeler, Thomsen, et al., 2022). The model was run for ∼81,000 locations across New Zealand. These locations are the result of the intersection of >10,000 localized weather grids (∼5 km^2^ grid with 41 years of daily weather data) (Cichota et al., 2008; Tait et al., 2006), with soil siblings from S‐Map (Landcare Research, 2014) present within each weather grid square. Later iterations of the tool (not reported here) also include slope classes broken into flat, rolling, easy, and steep slopes (0–7°, 7.1–15°, 15.1–25°, and >25°, respectively; AgResearch, 2016), resulting in >40 million outputs. Simulations were done both with and without irrigation and a single application of 450 kg N ha^−1^ made on the 15th of a given month (for each month in those 41 years) to a ryegrass‐white clover pasture. To assess relative transport risk by leaching, the amount of N leached beyond a depth of 1000 mm for 2 years after N application was divided by 450. To calculate relative transport risk via runoff, the amount of runoff in 30 days after the N addition was summed and then divided by 200 mm as the 95th percentile of maximum simulated runoff across New Zealand. Additional information about the construction and application of APSIM to calculate transport risk can be found in V. Snow et al. (2024).

Point‐based estimates of transport risk were then combined with sources of N loss recorded in kg N ha^−1^ at a block scale (a block being a group of fields under similar management). For leaching, these sources were animal deposits of dung and urine, fertilizer applications, aboveground (ag) and belowground (bg) crop residues. For runoff, these sources were animal deposits of dung, fertilizer applications, aboveground crop residues, and soil erosion. Urine was assumed to have been washed into the soil and unavailable to runoff. In equation form, monthly baseline risk is calculated as:

(1) $$\begin{array}{ccc} \text{Leaching index} & = & \sum \left(\left(\mathrm{leach}\times \mathrm{urine}\right)+\left(\mathrm{leach}\times \mathrm{dung}\right)\right.\\ & & \left.+\;\left(\mathrm{leach}\times \mathrm{fert}\right)\right.\\ & & \left.+\;\left(\mathrm{leach}\times \mathrm{bg}\mathrm{residues}\right)\right) \end{array}$$

(2) $$\begin{array}{ccc} \text{Runoff index} & = & \sum \left(\left(\mathrm{runoff}\times \mathrm{soil}\mathrm{erosion}\right)+\left(\mathrm{leach}\times \mathrm{dung}\right)\right.\\ & & \left.+\;\left(\mathrm{leach}\times \mathrm{fert}\right)+\left(\mathrm{leach}\times \mathrm{ag}\mathrm{residues}\right)\right) \end{array}$$

Baseline risk is then adjusted by applying mitigation actions. Mitigations can act by changing (δ) the baseline risk *sources* or by *modifying* runoff and leaching risks after baseline risk has been calculated (Figure 1).

## 3 CALCULATING SOURCES

All sources are input in kg N ha^−1^ for each month. The following outlines a summary of how each of the N sources (dung, urine, fertilizer, residues, and soil erosion) was calculated. More detail is in Tables S1–S18).

Data for livestock inputs of dung and urine were sourced from the New Zealand Agricultural Inventory used for reporting the New Zealand Greenhouse Gas emissions profile (Pickering et al., 2022) (Tables S1–S4). This method has undergone significant review and is regularly updated. The inventory isolates livestock into different age classes by region authorities for dairy cattle and age for beef cattle, sheep, and red deer. Single inputs are sourced for pigs, poultry, goats, horses, and alpacas. Nitrogen from dairy shed effluent is calculated using literature values for the N concentrations of wash down water and the N concentration of the effluent (Luo et al., 2022; Stewart & Rout, 2007).

The New Zealand Agricultural Inventory was also used to estimate the N concentration of crop residues (Tables S5–S9). These were augmented with additional data to yield N concentrations for approximately 30 cereal and vegetable crops (e.g., barley, wheat, oats, maize, onions, potatoes, brassicas, squash, peas, legumes, apples, vines, and avocados). The user selects what crop is grown on a block each month. Sources of N from above‐ and belowground residues were calculated after considering crop yield and the fraction of the crop left after harvest. Nitrogen was only released to the soil for the 3 months after harvest if the carbon:nitrogen ratio was <25 (Paul & Clark, 1989); otherwise, N was assumed to have been immobilized (and of no risk) (De Neve & Hofman, 1996). To account for N that could be caught by roots from a previous crop, the risk of N release from belowground residues was increased by 40% for shallow‐rooting crops and decreased by 30% for deep‐rooting crops. Finally, N inputs from the cultivation of short (<3 years old) and long‐term pastures under dairy or sheep and beef farming, and the associated mineralization of above‐ and belowground residues, along with any periods in fallow, were calculated using the method of Thomas et al. (2014).

Information on N inputs from fertilizer (Table S11) were sourced from user‐defined inputs of fertilizer products after adjusting them for their N concentration using published and verified laboratory analyses (Ballance Agri‐Nutrients, 2022; Ravensdown Fertiliser Co‐operative, 2022).

To estimate the source of N input from soil erosion loss via runoff, we first sourced 49 observations of sediment loss under different land uses (cropping, dairy, red deer, exotic and native forestry, sheep and beef, vegetables, and grazed winter forage crops) and slope classes (flat, rolling, easy, and steep) (Tables S12–S17). Estimates of annual and seasonal cover factors used in the Revised Universal Soil Loss Equation were taken from Donovan (2022) for New Zealand and from a European study for vegetables, as no data were available for New Zealand (Bakker et al., 2008). These data were adjusted by multipliers (from 70 to 800) to give sediment yields that closely matched median observed sediment yields (observed sediment yield = 0.92 × estimated sediment yield − 15.7, *r* ^2^ = 0.74, *p *< 0.05). Finally, sediment yields were combined with measurements of median soil total N concentration from sampling conducted by Regional Authorities from 1995 to 2017 and reported to the Ministry for the Environment and Statistics New Zealand as part of State of the Environment reporting (NZStats, 2022) to yield estimates of seasonal soil N losses via erosion (kg N ha^−1^) by land use (including grazed winter forage crops) and slope class.

## 4 INCORPORATING MITIGATION ACTIONS

We included mitigations (Table S18) in the tool that (1) were accessible and published, only including gray literature where the report was peer‐reviewed and there was no conflict of interest such as commercial gain for the commissioning agency; (2) had data sourced from multiple, and preferentially replicated, studies; and (3) was tested in a range of locations.

When implementing mitigations, they were filtered for their relevance to the land use (cropping, dairy, deer, sheep and beef, forestry, and horticulture), flow path (runoff or leaching), and then applied by either changing (δ; Figure 1) the baseline risk source inputs (urine, dung, fertilizer, and soil mineral N) or modifying baseline risk scores. Mitigation actions that change source inputs allow for multiple actions to be implemented by recalculating baseline risk (e.g., by changing stocking rate, which alters urine and dung N inputs). However, some mitigation actions, such as wetlands, do not act upon source inputs. We termed these actions—*modifiers*. Modifiers act by multiplying the baseline block risk score for runoff and leaching by a value between 0 and 1. We assumed that modifiers were used in the order of most to least effective and that any subsequent modifiers would act upon the product of the previous modifier. For example, let us assume a natural wetland that reduces risk by 20% is installed alongside a constructed wetland that reduces risk by 10%. A block with a risk score of 100 would be modified as 100 × 0.8 = 80, followed by 80 × 0.9 = 72. This process reflects the diminishing returns associated with the sequential implementation of multiple edge‐of‐field mitigations (McDowell et al., 2021). We do not account for potential synergies or antagonisms between practices in the implementation of modifiers. In other words, each practice is assumed to affect risk independently from the others.

We adjust modifiers to account for the influence of climate, slope, and soil type. We also assume that modifiers like a constructed wetland or a denitrification bed are placed in the optimal position to intercept runoff or leaching prior to exiting the block.

## 5 VALIDATION AND TESTING

**FIGURE 2:**
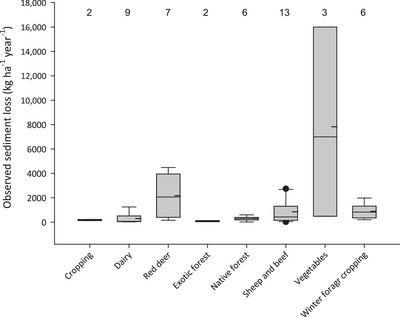
Box plots of observed sediment yields by land use in New Zealand showing the 25th, 50th, and 75th percentiles as the top, middle, and bottom of each box along with the 5th and 95th percentiles and any outliers (as filled circles). The dashed line refers the mean of data in each box while the numbers refer to the number of studies.

**FIGURE 3:**
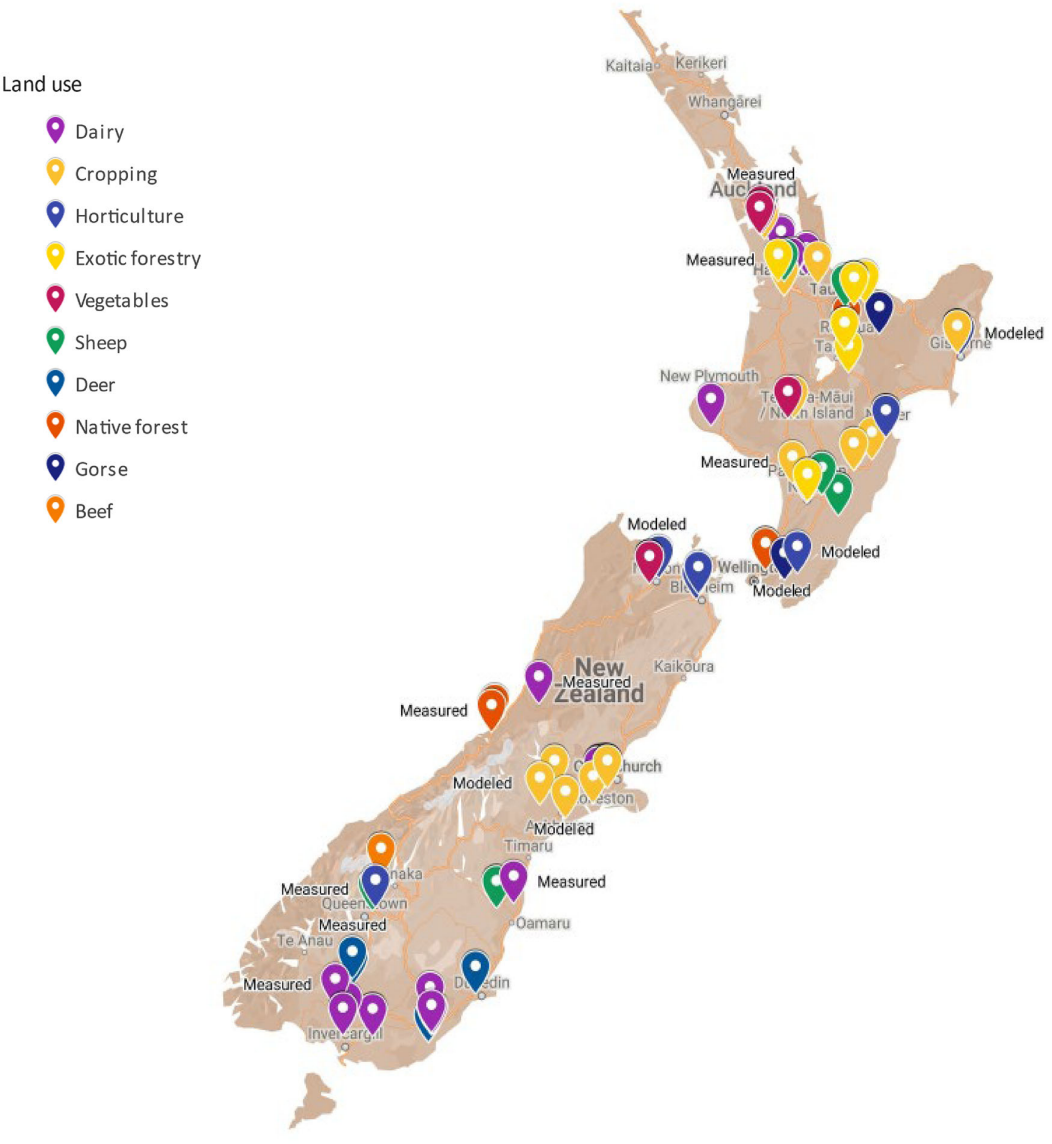
Map showing the location and dominant land use of N loss observations. Note that “gorse” land use data were not used in the development or testing of the tool but are included for reference.

**FIGURE 4:**
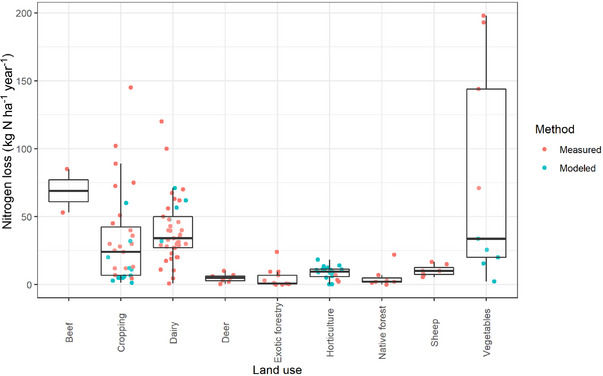
Nitrogen yields by land use and method (measured or modeled). Top, middle, and bottom of the boxes represent the 25th, 50th, and 75th percentiles, while the whiskers are the 5th and 95th percentiles for combined measured and modeled data.

**FIGURE 5:**
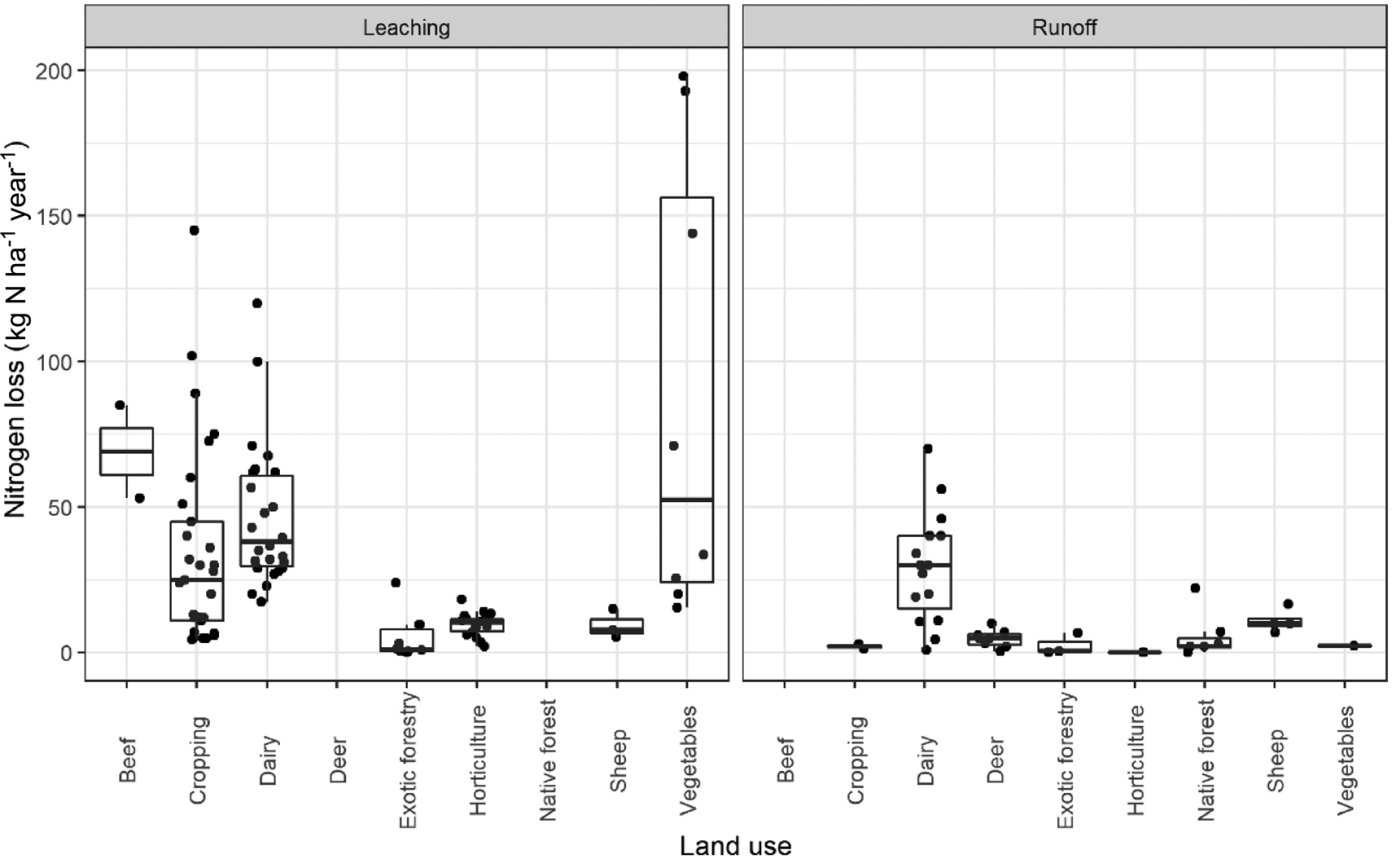
Nitrogen yields by land use and flow path (leaching and runoff). Top, middle, and bottom of the boxes represent the 25th, 50th, and 75th percentiles, while the whiskers are the 5th and 95th percentiles.

We collated a database containing 155 observations of N loss from 55 studies of different land uses (Drewry et al., 2022) (Figures 2 and 3). The database (Figure 4) contained 114 measured and 41 modeled observations via models validated to yield accurate estimates of daily N loss in New Zealand: APSIM and Soil Plant Atmosphere System Model (Cichota & Snow, 2009); 58 were of total N and 124 were of nitrate‐N; 51 were of runoff (often combining leaching and surface runoff) and 104 were of leaching losses (Figure 5). Among land uses, there were three observations for beef, 31 for cropping, 47 for dairy, eight for deer, 13 for exotic forestry, four for gorse, 21 for horticulture, seven for native forest, 10 for sheep, and 11 for vegetables. Additional data were collated for stocking rate (46 observations) and for annual N fertilizer application (97 observations) (Table 1).

**TABLE 1:** Mean (with standard deviation in parentheses) N loss and N fertilizer applied (both kg N ha^−1^ year^−1^) and stocking rate (stock units ha^−1^) for the unfiltered (*n* = 155) and filtered (*n* = 97) data by land use.

Land use	Unfiltered			Filtered		
	N loss	Fertilizer	Stocking rate^a^	N loss	Fertilizer	Stocking rate
Vegetables	65(76)	171(95)	–	52(67)	176(99)	–
Cropping	58(49)	154(109)	6(8)	23(29)	215(106)	–
Horticulture	8(6)	29(33)	–	10(6)	29(33)	–
Dairy	38(20)	161(88)	22(22)	44(19)	165(99)	22(18)
Native forest	5(8)	–	–	7(9)	–	–
Sheep/beef	21(7)	60(60)	12(4)	10(4)	60(60)	11(3)
Deer	7(2)	65	15(4)	7(2)	65	15(4)
Exotic forestry	7(2)	–	–	7(2)	–	–
Winter forage cropping^a^	69(42)	89(60)	312(482)	70(28)	–	517(678)

^a^ Stocking rate for dairy cattle is calculated for the 2010/2011 season (Livestock Improvement Cooproration Limited & DairyNZ Limited, 2021) with a mean production of 878 kg milksolids per cow, stocking rate of 2.76 cows ha^−1^ and a stock unit conversion of 7.3 for a cow of 400 kg liveweight (Reynish, 2018). Note that the stocking rate for winter forage cropping is calculated for when stock are grazing the crop not on a farm‐wide basis.

For each observation, we separated losses by flow path and inspected the original publication and data to obtain monthly estimates of fertilizer data and livestock numbers (namely, dung and urine inputs). Site‐specific data were used to generate the relevant transport risk from a digital elevation model formed from 10‐m resolution data and soil data, using S‐map auto‐generated factsheets (Landcare Research, 2014) if no data were available from the literature study. We recognize that S‐map soil mapping, published at 1:50,000 (regional) scale, only provides a coarse spatial estimate. We also estimated soil N concentration (NZStats, 2022) on a soil order by land use basis, and when soil N inputs were likely via cultivation, if converting from pasture, or from crop residues (Thomas et al., 2011, 2014). Observations were removed where there was low confidence in N inputs or the location or where observations were recorded at an inappropriate scale (e.g., catchment > 50 ha). This left 96 observations.

Using each observation's location, modeled transport risks were multiplied by monthly estimates of N sources. Overall risk was calculated for runoff and leaching, which for the observations was on average, 3.7 and 19.3, respectively.

**FIGURE 6:**
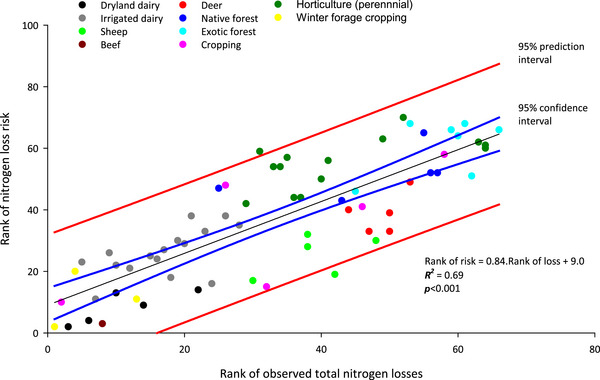
Plot of the rank of the risk (1 = greatest) of nitrogen loss against the rank of observed nitrogen loss. Ranks overcome clustering and the non‐normal distribution of the data allowing a regression equation (plus 95% confidence and prediction intervals) and coefficient of determination to be fitted.

Since some land uses had relatively few data or data that were clustered over different yields (Figures 4 and 5), data were non‐normally distributed. Risk scores were therefore converted into ranks before fitting a regression and the risks plotted against the observations (Figure 6).

**FIGURE 7:**
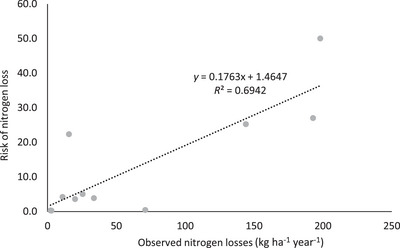
Plot of the risk of nitrogen loss against observed losses for vegetables. The regression fit is significant at the *p *< 0.05 level.

Vegetables were not included in the plot as risk index values were consistently lower than observed losses (Figure 7; slope ∼6 times too low). This is likely to be caused by the underprediction of N transport in shallow‐rooting vegetable crops compared to a deeper rooting ryegrass‐white clover pasture used as the baseline crop in APSIM.

We also note that some observed losses for cropping sites were out of sync with risk scores for the same year. This was caused by observations that were recorded in publications for hydrological years (September–August), but risks that were calculated on a calendar year (Table 2). Although this evened out when data for all years were averaged, these data suggest that the risk index should be calculated across multiple years if month‐by‐month risks are desired.

**TABLE 2:** Comparison of observed losses and risk scores over 6 years of ryegrass–wheat–barley–plantain rotation in mid‐Canterbury that was periodically grazed by livestock (Trolove et al., 2021).

Year	Management	Observed loss (kg N ha^−1^ year^−1)^	Risk score
1	25 lambs ha^−1^ in September and 17 in calf cows ha^−1^ in July	36	73
2		49	24
3		69	17
4	28 lambs ha^−1^ in September	20	45
5		31	26
6	17 cows ha^−1^ July/August, 22–31 lambs in September	13	60

**FIGURE 8:**
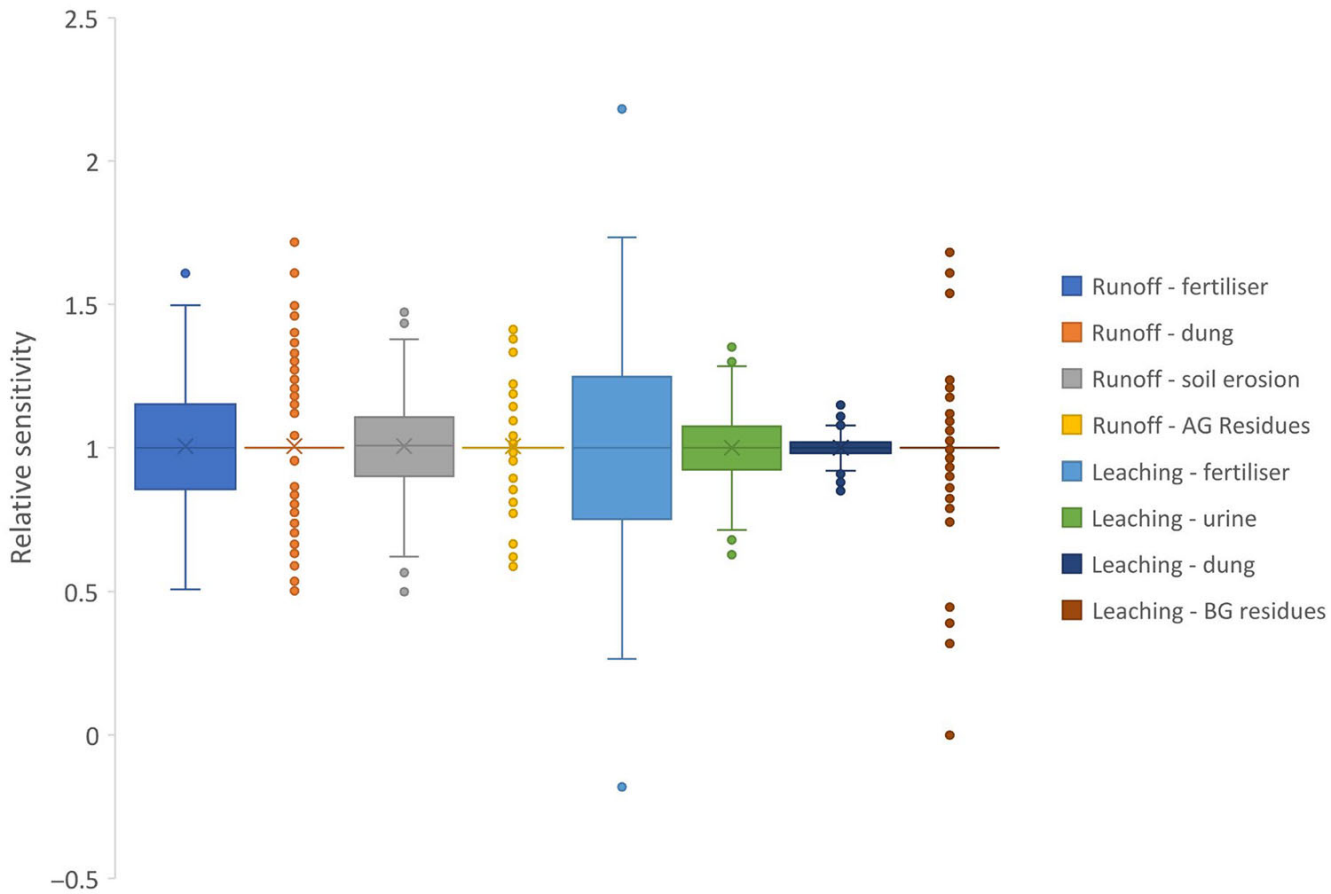
Sensitivity of increasing or decreasing different source factors by 50% on the risk of nitrogen loss as estimated for estimates of the filtered observed data. A sensitivity of 1 indicates that the site was insensitive to increases or decreases, which could reflect that the source was not applied at that site (e.g., no animals in a perennial horticulture site). AG and BG refer to above‐ and belowground, respectively.

To determine the sensitivity of sources on risk scores, we simulated 50% and 150% of the inputs captured across the filtered observed data and expressed their effect on the overall index score relative to the mean of the original data (Figure 8). Because we had a limited set of observations, outputs are unlikely to capture the sensitivity of the index across a broader range of possible inputs. The output (Figure 8) is split into the effect of inputs to runoff and leaching.

Very few data were available to test the efficacy of mitigations. However, in the original database, sufficient data (*n* = 88) were available to generate a regression relationship to predict the effect of fertilizer rates on nitrate‐N and TN losses (*r* ^2^ = 0.80) in pastoral systems (see Supporting Information) (Drewry et al., 2022). We therefore tested the effect of applying fertilizer at intervals of 30–40 kg N ha^−1^ year^−1^ up to the maximum allowable rate for dairy in New Zealand of 190 kg N ha^−1^ year^−1^ (spread across the growing season of October to May) (Ministry for the Environment, 2020). We used expert opinion to adjust stocking rate to reflect a reduction in N fertilizer (and feed). The output is shown in Table 3 alongside losses estimated from the model of N observations (*r* ^2^ = 0.80) with terms for land use, flow path, fertilizer rate, and irrigation (Drewry et al., 2022). This comparison estimates N loss risk to be about 20% lower, but more importantly, increasing at the same rate as estimates N losses.

**TABLE 3:** Estimates of N loss using and the commensurate risk score for a dairy farm in the Manawatu (Christensen et al., 2019).

Fertilizer/stocking rate (cow ha^−1^)	Predicted N loss as per Drewry et al. (2022) (kg ha^−1^ year^−1^)	Estimated risk score
30 (2.2)^a^	21	10
60 (2.6)	23	12
90 (2.8)	25	14
120 (3.0)	27	16
150 (3.1)	30	20
190 (3.3)	33	23

^a^ Stocking rate in cows ha^−1^ are in parentheses.

We determined that sense checking of the effect of modifiers was not required for two reasons: (1) owing to a paucity of data, we used all free, robust, and accessible studies to create modifiers—meaning that an independent set of data to check their performance was unavailable; and (2) no data were available to compare any potential interactions between modifiers. However, we note that the likelihood of the combined effect of two modifiers applied in parallel exceeding that of modifiers applied in series is low. In other words, most of the effect on risk is likely captured by the fact that modifiers are applied in the order of most to least effective, meaning that the less effective modifier will always have less N to reduce.

## 6 COMPARISON TO OTHER INDEX APPROACHES

Although our tool shares some similarities with nutrient indices in other jurisdictions, it is important to contrast and highlight what is novel about our tool. One of the main differences is how our tool uses and presents spatial data. Most N and P indices evaluate the risk of loss on a field‐by‐field basis (Bechmann et al., 2005; Sharpley et al., 2003), ignoring subfield spatial variation that could significantly alter the risk of N loss (Carrick et al., 2013; Vogeler, Lilburne, et al., 2022). Some advances have been made to map the influence of, for example, soil type (Delgado et al., 2010), but factors like slope and long‐term climate have seldom been considered. Reasons for this include a lack of data available to train the likelihood of transport across large areas and a focus on nitrate loss (ignoring non‐nitrate N forms). We used nationally available data for soils, slope, and climate and the well‐validated model APSIM to generate a national layer for transport risk.

To avoid arguments over the quality of source inputs and uphold standards for transparency, we chose to only use published, open data and linked some inputs to datasets already used for national and international reporting. For example, the Agricultural Inventory method used to estimate urine and dung N inputs from stock class, age, and region (for dairy) is used to report the national greenhouse gas profile from livestock. It is regularly updated ensuring that the tool will use the best data available.

One final, novel aspect of our tool is that it quantifies N loss risk from grazing livestock. In contrast to other indices that just focus on cropping systems or confined animal feeding operations (Figueroa‐Viramontes et al., 2016), livestock farming in New Zealand is the dominant land use occupying ∼95% of agricultural land (Ministry for the Environment & Statistics New Zealand, 2021). These livestock graze outdoors almost year‐round, and hence we need to consider their inputs as they move from one part of the farm to another as part of a planned (e.g., rotational) grazing system.

## 7 LIMITATIONS AND USE

Although developed for a range of land use and flow paths, we noted issues that may question or limit its use in certain circumstances. These issues can be broken into the way in which the tool represents some land uses and flow paths and a paucity of data to confirm if the tool's performance is “acceptable.”

During testing we identified the tool may underrepresent the leaching risk of transport for shallow‐rooted crops like vegetables compared to ryegrass‐white clover as the baseline crop. However, the degree of underrepresentation was consistent across a wide range of possible transport risks (0.1–0.7 out of a possible 0–1 scale). We have no data to confirm if shallow‐rooting crops are being underrepresented. To assess this potential issue, we would need to compare risk from a rotation of shallow‐rooting crops against ryegrass‐white clover in the same location. Until this is done, the consistent underrepresentation infers that risk can be scaled (by about 6) to approximate true risk.

Another issue we identified during testing was the temporal misalignment of month‐by‐month risk with the observations of annual N losses for cropping systems. This is likely caused by risks that are recorded “instantaneously” and N losses that are affected by time lags as N moves through the soil. This issue does not occur to the same degree in other land uses where changes caused by factors such as cultivation are less frequent or abrupt. Where lags between risk and observations are considered and synced, there was good agreement with observations. This is not a problem for management as the risk will eventually translate into a loss. However, it may be an issue should the tool be used at larger scales where lag times may delay a decrease in N loads beyond a desired timeframe. This might occur in, for example, jurisdictions that experience frozen soils. Here, risk might be predicted but not occur until the soil thaws. Using the tool in these jurisdictions could be enabled by incorporating a lag into the calculation of runoff risk.

In addition to identifying potential issues with how the tool functions, we also noted data gaps that raise the potential for some land uses or regions to be underrepresented. All land uses had some observations, but some had few (e.g., 8–13 for deer, sheep (only), exotic forestry, and vegetables). Furthermore, when separated by flow path, there were fewer observations of runoff than for leaching. This does not mean that calculated risks are wrong as the range of calculated risk was wide (and significantly related to N loss), but additional data should be collected to increase confidence in the range of outputs. Similarly, although there were few data for runoff, data were collected for land uses where runoff was most likely to occur (e.g., sloping land supporting deer, and sheep and beef farming), and confirmed to be a low proportion of total N losses under flat dairy land.

Data gaps can also exist spatially. There is a widespread distribution of observations (Figure 3), but only two of the 156 observations were recorded on land under Māori ownership (the indigenous people of New Zealand). The proportion of observations (<2%) on Māori‐owned land (1.3 M ha) is in proportion to the ratio of land owned and not owned by Māori (1.3/136 M ha or 1%) (Ministry for the Environment & Statistics New Zealand, 2021). However, Māori‐owned land tends to be steeper, restricting land use options, and under different ownership structures that may alter the risk of N loss. For example, if operated as a collective, Māori‐owned farms can make management decisions that are spread across multiple farms. These differences in slope and potential management infer that more data on Māori‐owned land are required to confirm the performance of the tool.

Having considered the limitations and performance of the tool, we assert that the tool can be used to inform and manage the risk of N loss from a range of land uses and flow paths. However, we recognize that this is a value judgment. Others may wish that the tool is used to estimate daily risk of N loss or to estimate absolute losses so it can be connected to catchment models. We resisted both approaches. While we could have estimated daily risks, we felt it unlikely that a user would maintain accurate records, thereby underestimating risk if sources are not recorded on the same day, for example, a runoff event. It is valid to say that monthly transport and input data will dilute the risk of loss, but others have already calculated that, on average, the dilution effect from capturing monthly data are less than the errors caused by the misalignment of daily data.

We also recognize that while the tool was developed and validated for New Zealand land uses and management, with some modification, the approach could be transferrable to other jurisdictions. Such modifications include potential calibration and testing of the transport model (APSIM), accounting for freeze‐thaw cycles, recalculation of the N concentration and array of crop residue types, and the inclusion of modifiers that have been validated locally.

The uncertainties in estimating absolute N loss without a complex process‐based model are potentially large. However, the complexity of process‐based models makes them unsuitable for widespread use owing to limited resources and the need for specific expertise. We chose a risk‐based approach as this would be more transparent, simpler to understand, and easily accessible. Nevertheless, we recognize that the tool's performance is limited by the data available for testing. Because these data do not represent the full range of management possibilities nor responses to mitigations and modifiers, we have presented outputs as a risk score expressed as a number. While the risk number is related to absolute (kg N ha^−1^ year^−1^) losses, the paucity of data required that we establish the link to absolute losses via non‐parametric methods. This means that the rank of land uses and the managements that they contain therein are related to absolute losses, but the risk number should not be used as a proxy for absolute losses in, for example, catchment models.

A link to the management of catchment water quality objectives can still be made with the outputs from the tool. Risk should be assessed and decreased by applying appropriate mitigations and modifiers (or land use change) in conjunction with water quality monitoring. The monitoring would be conducted at a combination of sentinel sites that respond quickly to management change and long‐term sites that show those changes at a larger scale. This way, management can be focused on showing that the direction of travel in decreasing risk is mirrored by sentinel and long‐term sites over the time that the objective is set. If no progress is being detected at the sentinel sites, risk must be lowered further.

## 8 CONCLUSIONS

The risk index tool combined a process understanding of transport risk associated with static soil, climate, and slope factors with dynamic inputs of N sources. Using the management and location data available for 156 observations of N loss, risks were quantified for both leaching and runoff across nine land uses. The risk of N loss by runoff was approximately 17% of total risk but may be greater owing to a paucity of runoff data (i.e., measured at 51 sites). The rank of risk was related to the rank of N loss (*r* ^2^ = 0.69, *p *< 0.001), with all but two of the 96 datapoints (filtered for data quality) fitting within the 95% prediction intervals. This suggests that the tool was able to successfully rank the risk of N loss between land uses and the variation within land uses. The tool may be useful in identifying where within a property the risk of N loss is greatest and targeting these high‐risk areas with actions to reduce the risk of N loss and improve water quality. The tool is based on published open access data and equations making it transparent and easily updated or repurposed.

## AUTHOR CONTRIBUTIONS

**R. W. McDowell**: Conceptualization; data curation; formal analysis; funding acquisition; investigation; methodology; project administration; resources; supervision; validation; writing—original draft; writing—review and editing. **V. O. Snow**: Data curation; formal analysis; investigation; methodology; validation; writing—original draft; writing—review and editing. **R. Tamepo**: Data curation; methodology; writing—original draft. **L. Lilburne**: Data curation; methodology; visualization; writing—review and editing. **R. Cichota**: Data curation; formal analysis; methodology; writing—review and editing. **K. Muraoka**: Conceptualization; methodology; writing—original draft; writing—review and editing. **E. Soal**: Conceptualization; writing—review and editing.

## CONFLICT OF INTEREST STATEMENT

The authors declare no conflicts of interest.

## Supporting information



Supplementary Information
