# Supplementary material for: A risk index tool to minimize the risk of nitrogen loss from land to water
Source: J Environ Qual. 2024 Dec 12;54(1):233–45. doi: 10.1002/jeq2.20660 (PMC11718142; doi:10.1002/jeq2.20660)
Supplement: Supplementary file 1 — Supplementary Information [file JEQ2-54-233-s001.docx]

**Supplementary Information**

**A risk index tool to minimise the risk of nitrogen loss from land to water**

R.W. McDowell ^1,2,*^, V.O. Snow^2^, R. Tamepo^3^, L. Lilburne^4^, R. Cichota^5^, K. Muraoka^6^, E. Soal^7^

*^1^ Faculty of Agriculture and Life Sciences, Lincoln University, Lincoln, New Zealand*

*^2^ AgResearch, Lincoln Science Centre, Lincoln, New Zealand*

*^3^ Scion Research, Rotorua, New Zealand*

*^4^ Manaaki Whenua Landcare Research, Lincoln, New Zealand*

*^5^ Plant and Food Research, Lincoln, New Zealand*

*^6^ Ministry for the Environment, Wellington, New Zealand*

*^7^ ES Water Policy, Oamaru, New Zealand*

*^*^Corresponding author’s email: richard.mcdowell@lincoln.ac.nz*

# Sources of N from dung and urine

The following Tables (S1-S4) outline the nitrogen (N) excretion rates for livestock to be used as input values for different stock classes, ages and rates for leaching and runoff.

If a block is identified as receiving effluent, the quantity of fertiliser N is boosted by the N contained in the effluent [1] and the daily volume of wash down water [2] (summed to 30 days from September to May) cycled through the effluent system and applied to land (assumes a travelling irrigator) where:

Effluent N (kg month^-1^) = Number of dairy cows

× 70 L cow^-1^ day^-1^

× 0.2 g N L^-1^

× 30 (days / month) / 1000 (g /kg)

No data are currently available for effluent from dairy sheep or goats.

The tables are based on the New Zealand Agricultural Inventory Model (AIM). AIM is designed for inventory purposes and contains some features not consistent with the RIT. The inconsistencies (<2% of values) are entries of zero excreta values for some months where, at a regional or national scale, an animal class is not present. The RIT is applied at a sub-farm level and so these assumptions can be problematic. In these cases, the excreta values were estimated from the existing data. Rows in the tables below that have been modified are highlighted in the “Notes” column with an explanation of the estimation method.

## Dairy cattle

**Table S1**. N excretion rates for different age classes of dairy cattle by region (2021-2022), sourced from Pickering, Gibbs [3]. Note that estimated values are in italics with the method indicated in a footnote to the Table.

| Region | Class | Month | Total Excreta kg N/head per month | Nitrogen Excreted In Urine kg N/head | Nitrogen Excreted In Faeces kg N/head |
| --- | --- | --- | --- | --- | --- |
| Auckland | Milking Cows - Mature | Jan | 9.06 | 6.63 | 2.43 |
| Auckland | Milking Cows - Mature | Feb | 7.85 | 5.75 | 2.1 |
| Auckland | Milking Cows - Mature | Mar | 9.6 | 7.03 | 2.57 |
| Auckland | Milking Cows - Mature | Apr | 8.73 | 6.4 | 2.34 |
| Auckland | Milking Cows - Mature | May | 8.14 | 5.96 | 2.18 |
| Auckland | Milking Cows - Mature | Jun | 7.22 | 5.29 | 1.93 |
| Auckland | Milking Cows - Mature | Jul | 9.16 | 6.71 | 2.45 |
| Auckland | Milking Cows - Mature | Aug | 8.33 | 6.1 | 2.23 |
| Auckland | Milking Cows - Mature | Sep | 10.97 | 8.03 | 2.94 |
| Auckland | Milking Cows - Mature | Oct | 10.78 | 7.89 | 2.89 |
| Auckland | Milking Cows - Mature | Nov | 10.05 | 7.36 | 2.69 |
| Auckland | Milking Cows - Mature | Dec | 9.78 | 7.16 | 2.62 |
| Auckland | Growing Heifers - 0-1 | Jan | 2.89 | 2.12 | 0.77 |
| Auckland | Growing Heifers - 0-1 | Feb | 2.86 | 2.1 | 0.77 |
| Auckland | Growing Heifers - 0-1 | Mar | 4.04 | 2.96 | 1.08 |
| Auckland | Growing Heifers - 0-1 | Apr | 4.23 | 3.09 | 1.13 |
| Auckland | Growing Heifers - 0-1 | May | 4.81 | 3.52 | 1.29 |
| Auckland | Growing Heifers - 0-1 | Jun | 4.76 | 3.49 | 1.27 |
| Auckland | Growing Heifers - 0-1 | Jul | 3.56 | 2.6 | *0.95** |
| Auckland | Growing Heifers - 0-1 | Aug | 3.56 | 2.6 | *0.95** |
| Auckland | Growing Heifers - 0-1 | Sep | 3.56 | 2.6 | *0.95** |
| Auckland | Growing Heifers - 0-1 | Oct | 1.87 | 1.37 | 0.5 |
| Auckland | Growing Heifers - 0-1 | Nov | 2.12 | 1.55 | 0.57 |
| Auckland | Growing Heifers - 0-1 | Dec | 2.5 | 1.83 | 0.67 |
| Auckland | Growing Heifers - 1-2 | Jan | 6.42 | 4.7 | 1.72 |
| Auckland | Growing Heifers - 1-2 | Feb | 6.04 | 4.42 | 1.62 |
| Auckland | Growing Heifers - 1-2 | Mar | 8.01 | 5.86 | 2.14 |
| Auckland | Growing Heifers - 1-2 | Apr | 7.88 | 5.77 | 2.11 |
| Auckland | Growing Heifers - 1-2 | May | 6.67 | 4.88 | *1.79*† |
| Auckland | Growing Heifers - 1-2 | Jun | 6.67 | 4.88 | *1.79*† |
| Auckland | Growing Heifers - 1-2 | Jul | 5.27 | 3.86 | 1.41 |
| Auckland | Growing Heifers - 1-2 | Aug | 5.52 | 4.04 | 1.48 |
| Auckland | Growing Heifers - 1-2 | Sep | 5.58 | 4.08 | 1.49 |
| Auckland | Growing Heifers - 1-2 | Oct | 5.32 | 3.9 | 1.43 |
| Auckland | Growing Heifers - 1-2 | Nov | 5.49 | 4.02 | 1.47 |
| Auckland | Growing Heifers - 1-2 | Dec | 6 | 4.39 | 1.61 |
| Auckland | Breeding Bulls | Jan | 8.13 | 5.96 | 2.18 |
| Auckland | Breeding Bulls | Feb | 7.38 | 5.4 | 1.98 |
| Auckland | Breeding Bulls | Mar | 9.42 | 6.9 | 2.52 |
| Auckland | Breeding Bulls | Apr | 8.94 | 6.55 | 2.39 |
| Auckland | Breeding Bulls | May | 9.36 | 6.86 | 2.51 |
| Auckland | Breeding Bulls | Jun | 8.6 | 6.3 | 2.3 |
| Auckland | Breeding Bulls | Jul | 8.93 | 6.54 | 2.39 |
| Auckland | Breeding Bulls | Aug | 8.77 | 6.42 | 2.35 |
| Auckland | Breeding Bulls | Sep | 8.37 | 6.13 | 2.24 |
| Auckland | Breeding Bulls | Oct | 7.63 | 5.59 | 2.04 |
| Auckland | Breeding Bulls | Nov | 7.51 | 5.5 | 2.01 |
| Auckland | Breeding Bulls | Dec | 7.84 | 5.74 | 2.1 |
| BOP | Milking Cows - Mature | Jan | 11.14 | 8.16 | 2.98 |
| BOP | Milking Cows - Mature | Feb | 9.53 | 6.98 | 2.55 |
| BOP | Milking Cows - Mature | Mar | 11.52 | 8.43 | 3.08 |
| BOP | Milking Cows - Mature | Apr | 10.19 | 7.47 | 2.73 |
| BOP | Milking Cows - Mature | May | 8.89 | 6.51 | 2.38 |
| BOP | Milking Cows - Mature | Jun | 7.37 | 5.39 | 1.97 |
| BOP | Milking Cows - Mature | Jul | 9.4 | 6.88 | 2.52 |
| BOP | Milking Cows - Mature | Aug | 9.72 | 7.11 | 2.6 |
| BOP | Milking Cows - Mature | Sep | 13.81 | 10.12 | 3.7 |
| BOP | Milking Cows - Mature | Oct | 13.74 | 10.06 | 3.68 |
| BOP | Milking Cows - Mature | Nov | 12.86 | 9.42 | 3.44 |
| BOP | Milking Cows - Mature | Dec | 12.33 | 9.03 | 3.3 |
| BOP | Growing Heifers - 0-1 | Jan | 2.9 | 2.13 | 0.78 |
| BOP | Growing Heifers - 0-1 | Feb | 2.88 | 2.11 | 0.77 |
| BOP | Growing Heifers - 0-1 | Mar | 4.06 | 2.97 | 1.09 |
| BOP | Growing Heifers - 0-1 | Apr | 4.25 | 3.11 | 1.14 |
| BOP | Growing Heifers - 0-1 | May | 4.84 | 3.54 | 1.3 |
| BOP | Growing Heifers - 0-1 | Jun | 4.79 | 3.51 | 1.28 |
| BOP | Growing Heifers - 0-1 | Jul | 3.58 | 2.62 | *0.96** |
| BOP | Growing Heifers - 0-1 | Aug | 3.58 | 2.62 | *0.96** |
| BOP | Growing Heifers - 0-1 | Sep | 3.58 | 2.62 | *0.96** |
| BOP | Growing Heifers - 0-1 | Oct | 1.88 | 1.38 | 0.5 |
| BOP | Growing Heifers - 0-1 | Nov | 2.13 | 1.56 | 0.57 |
| BOP | Growing Heifers - 0-1 | Dec | 2.52 | 1.84 | 0.67 |
| BOP | Growing Heifers - 1-2 | Jan | 6.45 | 4.73 | 1.73 |
| BOP | Growing Heifers - 1-2 | Feb | 6.07 | 4.44 | 1.62 |
| BOP | Growing Heifers - 1-2 | Mar | 8.05 | 5.89 | 2.15 |
| BOP | Growing Heifers - 1-2 | Apr | 7.92 | 5.8 | 2.12 |
| BOP | Growing Heifers - 1-2 | May | 6.71 | 4.91 | *1.79*† |
| BOP | Growing Heifers - 1-2 | Jun | 6.71 | 4.91 | *1.79*† |
| BOP | Growing Heifers - 1-2 | Jul | 5.3 | 3.88 | 1.42 |
| BOP | Growing Heifers - 1-2 | Aug | 5.55 | 4.06 | 1.48 |
| BOP | Growing Heifers - 1-2 | Sep | 5.61 | 4.11 | 1.5 |
| BOP | Growing Heifers - 1-2 | Oct | 5.35 | 3.92 | 1.43 |
| BOP | Growing Heifers - 1-2 | Nov | 5.52 | 4.04 | 1.48 |
| BOP | Growing Heifers - 1-2 | Dec | 6.03 | 4.41 | 1.61 |
| BOP | Breeding Bulls | Jan | 8.13 | 5.96 | 2.18 |
| BOP | Breeding Bulls | Feb | 7.38 | 5.4 | 1.98 |
| BOP | Breeding Bulls | Mar | 9.42 | 6.9 | 2.52 |
| BOP | Breeding Bulls | Apr | 8.94 | 6.55 | 2.39 |
| BOP | Breeding Bulls | May | 9.36 | 6.86 | 2.51 |
| BOP | Breeding Bulls | Jun | 8.6 | 6.3 | 2.3 |
| BOP | Breeding Bulls | Jul | 8.93 | 6.54 | 2.39 |
| BOP | Breeding Bulls | Aug | 8.77 | 6.42 | 2.35 |
| BOP | Breeding Bulls | Sep | 8.37 | 6.13 | 2.24 |
| BOP | Breeding Bulls | Oct | 7.63 | 5.59 | 2.04 |
| BOP | Breeding Bulls | Nov | 7.51 | 5.5 | 2.01 |
| BOP | Breeding Bulls | Dec | 7.84 | 5.74 | 2.1 |
| Canterbury | Milking Cows - Mature | Jan | 12.59 | 9.22 | 3.37 |
| Canterbury | Milking Cows - Mature | Feb | 10.71 | 7.84 | 2.87 |
| Canterbury | Milking Cows - Mature | Mar | 12.87 | 9.43 | 3.45 |
| Canterbury | Milking Cows - Mature | Apr | 11.23 | 8.22 | 3.01 |
| Canterbury | Milking Cows - Mature | May | 9.53 | 6.98 | 2.55 |
| Canterbury | Milking Cows - Mature | Jun | 7.49 | 5.48 | 2 |
| Canterbury | Milking Cows - Mature | Jul | 9.48 | 6.94 | 2.54 |
| Canterbury | Milking Cows - Mature | Aug | 10.17 | 7.45 | 2.72 |
| Canterbury | Milking Cows - Mature | Sep | 14.74 | 10.79 | 3.95 |
| Canterbury | Milking Cows - Mature | Oct | 14.68 | 10.75 | 3.93 |
| Canterbury | Milking Cows - Mature | Nov | 13.76 | 10.08 | 3.68 |
| Canterbury | Milking Cows - Mature | Dec | 13.15 | 9.63 | 3.52 |
| Canterbury | Growing Heifers - 0-1 | Jan | 2.91 | 2.13 | 0.78 |
| Canterbury | Growing Heifers - 0-1 | Feb | 2.89 | 2.12 | 0.77 |
| Canterbury | Growing Heifers - 0-1 | Mar | 4.08 | 2.99 | 1.09 |
| Canterbury | Growing Heifers - 0-1 | Apr | 4.26 | 3.12 | 1.14 |
| Canterbury | Growing Heifers - 0-1 | May | 4.86 | 3.56 | 1.3 |
| Canterbury | Growing Heifers - 0-1 | Jun | 4.81 | 3.52 | 1.29 |
| Canterbury | Growing Heifers - 0-1 | Jul | 3.59 | 2.63 | *0.96** |
| Canterbury | Growing Heifers - 0-1 | Aug | 3.59 | 2.63 | *0.96** |
| Canterbury | Growing Heifers - 0-1 | Sep | 3.59 | 2.63 | *0.96** |
| Canterbury | Growing Heifers - 0-1 | Oct | 1.88 | 1.38 | 0.5 |
| Canterbury | Growing Heifers - 0-1 | Nov | 2.14 | 1.56 | 0.57 |
| Canterbury | Growing Heifers - 0-1 | Dec | 2.53 | 1.85 | 0.68 |
| Canterbury | Growing Heifers - 1-2 | Jan | 6.48 | 4.74 | 1.73 |
| Canterbury | Growing Heifers - 1-2 | Feb | 6.09 | 4.46 | 1.63 |
| Canterbury | Growing Heifers - 1-2 | Mar | 8.08 | 5.91 | 2.16 |
| Canterbury | Growing Heifers - 1-2 | Apr | 7.94 | 5.82 | 2.13 |
| Canterbury | Growing Heifers - 1-2 | May | 6.73 | 4.93 | *1.8*† |
| Canterbury | Growing Heifers - 1-2 | Jun | 6.73 | 4.93 | *1.8*† |
| Canterbury | Growing Heifers - 1-2 | Jul | 5.32 | 3.89 | 1.42 |
| Canterbury | Growing Heifers - 1-2 | Aug | 5.57 | 4.08 | 1.49 |
| Canterbury | Growing Heifers - 1-2 | Sep | 5.63 | 4.12 | 1.51 |
| Canterbury | Growing Heifers - 1-2 | Oct | 5.37 | 3.93 | 1.44 |
| Canterbury | Growing Heifers - 1-2 | Nov | 5.54 | 4.06 | 1.48 |
| Canterbury | Growing Heifers - 1-2 | Dec | 6.05 | 4.43 | 1.62 |
| Canterbury | Breeding Bulls | Jan | 8.13 | 5.96 | 2.18 |
| Canterbury | Breeding Bulls | Feb | 7.38 | 5.4 | 1.98 |
| Canterbury | Breeding Bulls | Mar | 9.42 | 6.9 | 2.52 |
| Canterbury | Breeding Bulls | Apr | 8.94 | 6.55 | 2.39 |
| Canterbury | Breeding Bulls | May | 9.36 | 6.86 | 2.51 |
| Canterbury | Breeding Bulls | Jun | 8.6 | 6.3 | 2.3 |
| Canterbury | Breeding Bulls | Jul | 8.93 | 6.54 | 2.39 |
| Canterbury | Breeding Bulls | Aug | 8.77 | 6.42 | 2.35 |
| Canterbury | Breeding Bulls | Sep | 8.37 | 6.13 | 2.24 |
| Canterbury | Breeding Bulls | Oct | 7.63 | 5.59 | 2.04 |
| Canterbury | Breeding Bulls | Nov | 7.51 | 5.5 | 2.01 |
| Canterbury | Breeding Bulls | Dec | 7.84 | 5.74 | 2.1 |
| Gisborne | Milking Cows - Mature | Jan | 9.23 | 6.76 | 2.47 |
| Gisborne | Milking Cows - Mature | Feb | 7.99 | 5.85 | 2.14 |
| Gisborne | Milking Cows - Mature | Mar | 9.77 | 7.15 | 2.61 |
| Gisborne | Milking Cows - Mature | Apr | 8.86 | 6.49 | 2.37 |
| Gisborne | Milking Cows - Mature | May | 8.25 | 6.04 | 2.21 |
| Gisborne | Milking Cows - Mature | Jun | 7.26 | 5.31 | 1.94 |
| Gisborne | Milking Cows - Mature | Jul | 9.11 | 6.67 | 2.44 |
| Gisborne | Milking Cows - Mature | Aug | 7.88 | 5.77 | 2.11 |
| Gisborne | Milking Cows - Mature | Sep | 10.02 | 7.34 | 2.68 |
| Gisborne | Milking Cows - Mature | Oct | 9.78 | 7.16 | 2.62 |
| Gisborne | Milking Cows - Mature | Nov | 9.1 | 6.66 | 2.44 |
| Gisborne | Milking Cows - Mature | Dec | 8.92 | 6.53 | 2.39 |
| Gisborne | Growing Heifers - 0-1 | Jan | 2.9 | 2.13 | 0.78 |
| Gisborne | Growing Heifers - 0-1 | Feb | 2.88 | 2.11 | 0.77 |
| Gisborne | Growing Heifers - 0-1 | Mar | 4.06 | 2.97 | 1.09 |
| Gisborne | Growing Heifers - 0-1 | Apr | 4.25 | 3.11 | 1.14 |
| Gisborne | Growing Heifers - 0-1 | May | 4.84 | 3.54 | 1.3 |
| Gisborne | Growing Heifers - 0-1 | Jun | 4.79 | 3.51 | 1.28 |
| Gisborne | Growing Heifers - 0-1 | Jul | 3.58 | 2.62 | *0.96** |
| Gisborne | Growing Heifers - 0-1 | Aug | 3.58 | 2.62 | *0.96** |
| Gisborne | Growing Heifers - 0-1 | Sep | 3.58 | 2.62 | *0.96** |
| Gisborne | Growing Heifers - 0-1 | Oct | 1.88 | 1.38 | 0.5 |
| Gisborne | Growing Heifers - 0-1 | Nov | 2.13 | 1.56 | 0.57 |
| Gisborne | Growing Heifers - 0-1 | Dec | 2.52 | 1.84 | 0.67 |
| Gisborne | Growing Heifers - 1-2 | Jan | 6.45 | 4.73 | 1.73 |
| Gisborne | Growing Heifers - 1-2 | Feb | 6.07 | 4.44 | 1.62 |
| Gisborne | Growing Heifers - 1-2 | Mar | 8.05 | 5.89 | 2.15 |
| Gisborne | Growing Heifers - 1-2 | Apr | 7.92 | 5.8 | 2.12 |
| Gisborne | Growing Heifers - 1-2 | May | 6.71 | 4.91 | *1.79*† |
| Gisborne | Growing Heifers - 1-2 | Jun | 6.71 | 4.91 | *1.79*† |
| Gisborne | Growing Heifers - 1-2 | Jul | 5.3 | 3.88 | 1.42 |
| Gisborne | Growing Heifers - 1-2 | Aug | 5.55 | 4.06 | 1.48 |
| Gisborne | Growing Heifers - 1-2 | Sep | 5.61 | 4.11 | 1.5 |
| Gisborne | Growing Heifers - 1-2 | Oct | 5.35 | 3.92 | 1.43 |
| Gisborne | Growing Heifers - 1-2 | Nov | 5.52 | 4.04 | 1.48 |
| Gisborne | Growing Heifers - 1-2 | Dec | 6.03 | 4.41 | 1.61 |
| Gisborne | Breeding Bulls | Jan | 8.13 | 5.96 | 2.18 |
| Gisborne | Breeding Bulls | Feb | 7.38 | 5.4 | 1.98 |
| Gisborne | Breeding Bulls | Mar | 9.42 | 6.9 | 2.52 |
| Gisborne | Breeding Bulls | Apr | 8.94 | 6.55 | 2.39 |
| Gisborne | Breeding Bulls | May | 9.36 | 6.86 | 2.51 |
| Gisborne | Breeding Bulls | Jun | 8.6 | 6.3 | 2.3 |
| Gisborne | Breeding Bulls | Jul | 8.93 | 6.54 | 2.39 |
| Gisborne | Breeding Bulls | Aug | 8.77 | 6.42 | 2.35 |
| Gisborne | Breeding Bulls | Sep | 8.37 | 6.13 | 2.24 |
| Gisborne | Breeding Bulls | Oct | 7.63 | 5.59 | 2.04 |
| Gisborne | Breeding Bulls | Nov | 7.51 | 5.5 | 2.01 |
| Gisborne | Breeding Bulls | Dec | 7.84 | 5.74 | 2.1 |
| Hawkes Bay | Milking Cows - Mature | Jan | 11.97 | 8.77 | 3.2 |
| Hawkes Bay | Milking Cows - Mature | Feb | 10.21 | 7.47 | 2.73 |
| Hawkes Bay | Milking Cows - Mature | Mar | 12.29 | 9 | 3.29 |
| Hawkes Bay | Milking Cows - Mature | Apr | 10.79 | 7.9 | 2.89 |
| Hawkes Bay | Milking Cows - Mature | May | 9.1 | 6.66 | 2.44 |
| Hawkes Bay | Milking Cows - Mature | Jun | 7.41 | 5.43 | 1.98 |
| Hawkes Bay | Milking Cows - Mature | Jul | 9.59 | 7.02 | 2.57 |
| Hawkes Bay | Milking Cows - Mature | Aug | 10.73 | 7.86 | 2.87 |
| Hawkes Bay | Milking Cows - Mature | Sep | 15.89 | 11.63 | 4.25 |
| Hawkes Bay | Milking Cows - Mature | Oct | 15.88 | 11.63 | 4.25 |
| Hawkes Bay | Milking Cows - Mature | Nov | 14.91 | 10.92 | 3.99 |
| Hawkes Bay | Milking Cows - Mature | Dec | 14.19 | 10.39 | 3.8 |
| Hawkes Bay | Growing Heifers - 0-1 | Jan | 2.91 | 2.13 | 0.78 |
| Hawkes Bay | Growing Heifers - 0-1 | Feb | 2.89 | 2.12 | 0.77 |
| Hawkes Bay | Growing Heifers - 0-1 | Mar | 4.08 | 2.99 | 1.09 |
| Hawkes Bay | Growing Heifers - 0-1 | Apr | 4.26 | 3.12 | 1.14 |
| Hawkes Bay | Growing Heifers - 0-1 | May | 4.86 | 3.56 | 1.3 |
| Hawkes Bay | Growing Heifers - 0-1 | Jun | 4.81 | 3.52 | 1.29 |
| Hawkes Bay | Growing Heifers - 0-1 | Jul | 3.59 | 2.63 | *0.96** |
| Hawkes Bay | Growing Heifers - 0-1 | Aug | 3.59 | 2.63 | *0.96** |
| Hawkes Bay | Growing Heifers - 0-1 | Sep | 3.59 | 2.63 | *0.96** |
| Hawkes Bay | Growing Heifers - 0-1 | Oct | 1.89 | 1.38 | 0.51 |
| Hawkes Bay | Growing Heifers - 0-1 | Nov | 2.14 | 1.57 | 0.57 |
| Hawkes Bay | Growing Heifers - 0-1 | Dec | 2.54 | 1.86 | 0.68 |
| Hawkes Bay | Growing Heifers - 1-2 | Jan | 6.48 | 4.74 | 1.73 |
| Hawkes Bay | Growing Heifers - 1-2 | Feb | 6.09 | 4.46 | 1.63 |
| Hawkes Bay | Growing Heifers - 1-2 | Mar | 8.08 | 5.91 | 2.16 |
| Hawkes Bay | Growing Heifers - 1-2 | Apr | 7.94 | 5.82 | 2.13 |
| Hawkes Bay | Growing Heifers - 1-2 | May | 6.74 | 4.93 | *1.81*† |
| Hawkes Bay | Growing Heifers - 1-2 | Jun | 6.74 | 4.93 | *1.81*† |
| Hawkes Bay | Growing Heifers - 1-2 | Jul | 5.34 | 3.91 | 1.43 |
| Hawkes Bay | Growing Heifers - 1-2 | Aug | 5.59 | 4.09 | 1.5 |
| Hawkes Bay | Growing Heifers - 1-2 | Sep | 5.65 | 4.14 | 1.51 |
| Hawkes Bay | Growing Heifers - 1-2 | Oct | 5.39 | 3.95 | 1.44 |
| Hawkes Bay | Growing Heifers - 1-2 | Nov | 5.56 | 4.07 | 1.49 |
| Hawkes Bay | Growing Heifers - 1-2 | Dec | 6.07 | 4.45 | 1.63 |
| Hawkes Bay | Breeding Bulls | Jan | 8.13 | 5.96 | 2.18 |
| Hawkes Bay | Breeding Bulls | Feb | 7.38 | 5.4 | 1.98 |
| Hawkes Bay | Breeding Bulls | Mar | 9.42 | 6.9 | 2.52 |
| Hawkes Bay | Breeding Bulls | Apr | 8.94 | 6.55 | 2.39 |
| Hawkes Bay | Breeding Bulls | May | 9.36 | 6.86 | 2.51 |
| Hawkes Bay | Breeding Bulls | Jun | 8.6 | 6.3 | 2.3 |
| Hawkes Bay | Breeding Bulls | Jul | 8.93 | 6.54 | 2.39 |
| Hawkes Bay | Breeding Bulls | Aug | 8.77 | 6.42 | 2.35 |
| Hawkes Bay | Breeding Bulls | Sep | 8.37 | 6.13 | 2.24 |
| Hawkes Bay | Breeding Bulls | Oct | 7.63 | 5.59 | 2.04 |
| Hawkes Bay | Breeding Bulls | Nov | 7.51 | 5.5 | 2.01 |
| Hawkes Bay | Breeding Bulls | Dec | 7.84 | 5.74 | 2.1 |
| Manawatu Wanganui | Milking Cows - Mature | Jan | 10.79 | 7.9 | 2.89 |
| Manawatu Wanganui | Milking Cows - Mature | Feb | 9.25 | 6.77 | 2.48 |
| Manawatu Wanganui | Milking Cows - Mature | Mar | 11.2 | 8.2 | 3 |
| Manawatu Wanganui | Milking Cows - Mature | Apr | 9.96 | 7.29 | 2.67 |
| Manawatu Wanganui | Milking Cows - Mature | May | 8.77 | 6.42 | 2.35 |
| Manawatu Wanganui | Milking Cows - Mature | Jun | 7.36 | 5.39 | 1.97 |
| Manawatu Wanganui | Milking Cows - Mature | Jul | 9.4 | 6.88 | 2.52 |
| Manawatu Wanganui | Milking Cows - Mature | Aug | 9.58 | 7.01 | 2.56 |
| Manawatu Wanganui | Milking Cows - Mature | Sep | 13.49 | 9.88 | 3.61 |
| Manawatu Wanganui | Milking Cows - Mature | Oct | 13.4 | 9.81 | 3.59 |
| Manawatu Wanganui | Milking Cows - Mature | Nov | 12.54 | 9.18 | 3.36 |
| Manawatu Wanganui | Milking Cows - Mature | Dec | 12.04 | 8.82 | 3.22 |
| Manawatu Wanganui | Growing Heifers - 0-1 | Jan | 2.91 | 2.13 | 0.78 |
| Manawatu Wanganui | Growing Heifers - 0-1 | Feb | 2.89 | 2.12 | 0.77 |
| Manawatu Wanganui | Growing Heifers - 0-1 | Mar | 4.08 | 2.99 | 1.09 |
| Manawatu Wanganui | Growing Heifers - 0-1 | Apr | 4.26 | 3.12 | 1.14 |
| Manawatu Wanganui | Growing Heifers - 0-1 | May | 4.86 | 3.56 | 1.3 |
| Manawatu Wanganui | Growing Heifers - 0-1 | Jun | 4.81 | 3.52 | 1.29 |
| Manawatu Wanganui | Growing Heifers - 0-1 | Jul | 3.59 | 2.63 | *0.96** |
| Manawatu Wanganui | Growing Heifers - 0-1 | Aug | 3.59 | 2.63 | *0.96** |
| Manawatu Wanganui | Growing Heifers - 0-1 | Sep | 3.59 | 2.63 | *0.96** |
| Manawatu Wanganui | Growing Heifers - 0-1 | Oct | 1.89 | 1.38 | 0.51 |
| Manawatu Wanganui | Growing Heifers - 0-1 | Nov | 2.14 | 1.57 | 0.57 |
| Manawatu Wanganui | Growing Heifers - 0-1 | Dec | 2.54 | 1.86 | 0.68 |
| Manawatu Wanganui | Growing Heifers - 1-2 | Jan | 6.48 | 4.74 | 1.73 |
| Manawatu Wanganui | Growing Heifers - 1-2 | Feb | 6.09 | 4.46 | 1.63 |
| Manawatu Wanganui | Growing Heifers - 1-2 | Mar | 8.08 | 5.91 | 2.16 |
| Manawatu Wanganui | Growing Heifers - 1-2 | Apr | 7.94 | 5.82 | 2.13 |
| Manawatu Wanganui | Growing Heifers - 1-2 | May | 6.74 | 4.93 | *1.81*† |
| Manawatu Wanganui | Growing Heifers - 1-2 | Jun | 6.74 | 4.93 | *1.81*† |
| Manawatu Wanganui | Growing Heifers - 1-2 | Jul | 5.34 | 3.91 | 1.43 |
| Manawatu Wanganui | Growing Heifers - 1-2 | Aug | 5.59 | 4.09 | 1.5 |
| Manawatu Wanganui | Growing Heifers - 1-2 | Sep | 5.65 | 4.14 | 1.51 |
| Manawatu Wanganui | Growing Heifers - 1-2 | Oct | 5.39 | 3.95 | 1.44 |
| Manawatu Wanganui | Growing Heifers - 1-2 | Nov | 5.56 | 4.07 | 1.49 |
| Manawatu Wanganui | Growing Heifers - 1-2 | Dec | 6.07 | 4.45 | 1.63 |
| Manawatu Wanganui | Breeding Bulls | Jan | 8.13 | 5.96 | 2.18 |
| Manawatu Wanganui | Breeding Bulls | Feb | 7.38 | 5.4 | 1.98 |
| Manawatu Wanganui | Breeding Bulls | Mar | 9.42 | 6.9 | 2.52 |
| Manawatu Wanganui | Breeding Bulls | Apr | 8.94 | 6.55 | 2.39 |
| Manawatu Wanganui | Breeding Bulls | May | 9.36 | 6.86 | 2.51 |
| Manawatu Wanganui | Breeding Bulls | Jun | 8.6 | 6.3 | 2.3 |
| Manawatu Wanganui | Breeding Bulls | Jul | 8.93 | 6.54 | 2.39 |
| Manawatu Wanganui | Breeding Bulls | Aug | 8.77 | 6.42 | 2.35 |
| Manawatu Wanganui | Breeding Bulls | Sep | 8.37 | 6.13 | 2.24 |
| Manawatu Wanganui | Breeding Bulls | Oct | 7.63 | 5.59 | 2.04 |
| Manawatu Wanganui | Breeding Bulls | Nov | 7.51 | 5.5 | 2.01 |
| Manawatu Wanganui | Breeding Bulls | Dec | 7.84 | 5.74 | 2.1 |
| Marlborough | Milking Cows - Mature | Jan | 12.99 | 9.51 | 3.48 |
| Marlborough | Milking Cows - Mature | Feb | 11.03 | 8.08 | 2.95 |
| Marlborough | Milking Cows - Mature | Mar | 13.23 | 9.68 | 3.54 |
| Marlborough | Milking Cows - Mature | Apr | 11.5 | 8.42 | 3.08 |
| Marlborough | Milking Cows - Mature | May | 9.65 | 7.06 | 2.58 |
| Marlborough | Milking Cows - Mature | Jun | 7.51 | 5.5 | 2.01 |
| Marlborough | Milking Cows - Mature | Jul | 9.52 | 6.97 | 2.55 |
| Marlborough | Milking Cows - Mature | Aug | 10.4 | 7.62 | 2.79 |
| Marlborough | Milking Cows - Mature | Sep | 15.22 | 11.15 | 4.08 |
| Marlborough | Milking Cows - Mature | Oct | 15.21 | 11.14 | 4.07 |
| Marlborough | Milking Cows - Mature | Nov | 14.26 | 10.44 | 3.82 |
| Marlborough | Milking Cows - Mature | Dec | 13.6 | 9.96 | 3.64 |
| Marlborough | Growing Heifers - 0-1 | Jan | 2.91 | 2.13 | 0.78 |
| Marlborough | Growing Heifers - 0-1 | Feb | 2.89 | 2.12 | 0.77 |
| Marlborough | Growing Heifers - 0-1 | Mar | 4.08 | 2.99 | 1.09 |
| Marlborough | Growing Heifers - 0-1 | Apr | 4.26 | 3.12 | 1.14 |
| Marlborough | Growing Heifers - 0-1 | May | 4.86 | 3.56 | 1.3 |
| Marlborough | Growing Heifers - 0-1 | Jun | 4.81 | 3.52 | 1.29 |
| Marlborough | Growing Heifers - 0-1 | Jul | 3.59 | 2.63 | *0.96** |
| Marlborough | Growing Heifers - 0-1 | Aug | 3.59 | 2.63 | *0.96** |
| Marlborough | Growing Heifers - 0-1 | Sep | 3.59 | 2.63 | *0.96** |
| Marlborough | Growing Heifers - 0-1 | Oct | 1.88 | 1.38 | 0.5 |
| Marlborough | Growing Heifers - 0-1 | Nov | 2.14 | 1.56 | 0.57 |
| Marlborough | Growing Heifers - 0-1 | Dec | 2.53 | 1.85 | 0.68 |
| Marlborough | Growing Heifers - 1-2 | Jan | 6.48 | 4.74 | 1.73 |
| Marlborough | Growing Heifers - 1-2 | Feb | 6.09 | 4.46 | 1.63 |
| Marlborough | Growing Heifers - 1-2 | Mar | 8.08 | 5.91 | 2.16 |
| Marlborough | Growing Heifers - 1-2 | Apr | 7.94 | 5.82 | 2.13 |
| Marlborough | Growing Heifers - 1-2 | May | 6.73 | 4.93 | *1.8*† |
| Marlborough | Growing Heifers - 1-2 | Jun | 6.73 | 4.93 | *1.8*† |
| Marlborough | Growing Heifers - 1-2 | Jul | 5.32 | 3.89 | 1.42 |
| Marlborough | Growing Heifers - 1-2 | Aug | 5.57 | 4.08 | 1.49 |
| Marlborough | Growing Heifers - 1-2 | Sep | 5.63 | 4.12 | 1.51 |
| Marlborough | Growing Heifers - 1-2 | Oct | 5.37 | 3.93 | 1.44 |
| Marlborough | Growing Heifers - 1-2 | Nov | 5.54 | 4.06 | 1.48 |
| Marlborough | Growing Heifers - 1-2 | Dec | 6.05 | 4.43 | 1.62 |
| Marlborough | Breeding Bulls | Jan | 8.13 | 5.96 | 2.18 |
| Marlborough | Breeding Bulls | Feb | 7.38 | 5.4 | 1.98 |
| Marlborough | Breeding Bulls | Mar | 9.42 | 6.9 | 2.52 |
| Marlborough | Breeding Bulls | Apr | 8.94 | 6.55 | 2.39 |
| Marlborough | Breeding Bulls | May | 9.36 | 6.86 | 2.51 |
| Marlborough | Breeding Bulls | Jun | 8.6 | 6.3 | 2.3 |
| Marlborough | Breeding Bulls | Jul | 8.93 | 6.54 | 2.39 |
| Marlborough | Breeding Bulls | Aug | 8.77 | 6.42 | 2.35 |
| Marlborough | Breeding Bulls | Sep | 8.37 | 6.13 | 2.24 |
| Marlborough | Breeding Bulls | Oct | 7.63 | 5.59 | 2.04 |
| Marlborough | Breeding Bulls | Nov | 7.51 | 5.5 | 2.01 |
| Marlborough | Breeding Bulls | Dec | 7.84 | 5.74 | 2.1 |
| Nelson | Milking Cows - Mature | Jan | 9.98 | 7.31 | 2.67 |
| Nelson | Milking Cows - Mature | Feb | 8.58 | 6.28 | 2.3 |
| Nelson | Milking Cows - Mature | Mar | 10.42 | 7.63 | 2.79 |
| Nelson | Milking Cows - Mature | Apr | 9.35 | 6.85 | 2.5 |
| Nelson | Milking Cows - Mature | May | 8.47 | 6.2 | 2.27 |
| Nelson | Milking Cows - Mature | Jun | 7.22 | 5.28 | 1.93 |
| Nelson | Milking Cows - Mature | Jul | 9.13 | 6.68 | 2.44 |
| Nelson | Milking Cows - Mature | Aug | 8.47 | 6.2 | 2.27 |
| Nelson | Milking Cows - Mature | Sep | 11.35 | 8.31 | 3.04 |
| Nelson | Milking Cows - Mature | Oct | 11.19 | 8.19 | 2.99 |
| Nelson | Milking Cows - Mature | Nov | 10.42 | 7.63 | 2.79 |
| Nelson | Milking Cows - Mature | Dec | 10.1 | 7.4 | 2.7 |
| Nelson | Growing Heifers - 0-1 | Jan | 2.83 | 2.08 | 0.76 |
| Nelson | Growing Heifers - 0-1 | Feb | 2.81 | 2.06 | 0.75 |
| Nelson | Growing Heifers - 0-1 | Mar | 3.96 | 2.9 | 1.06 |
| Nelson | Growing Heifers - 0-1 | Apr | 4.14 | 3.03 | 1.11 |
| Nelson | Growing Heifers - 0-1 | May | 4.72 | 3.45 | 1.26 |
| Nelson | Growing Heifers - 0-1 | Jun | 4.67 | 3.42 | 1.25 |
| Nelson | Growing Heifers - 0-1 | Jul | 3.49 | 2.55 | *0.93** |
| Nelson | Growing Heifers - 0-1 | Aug | 3.49 | 2.55 | *0.93** |
| Nelson | Growing Heifers - 0-1 | Sep | 3.49 | 2.55 | *0.93** |
| Nelson | Growing Heifers - 0-1 | Oct | 1.83 | 1.34 | 0.49 |
| Nelson | Growing Heifers - 0-1 | Nov | 2.08 | 1.52 | 0.56 |
| Nelson | Growing Heifers - 0-1 | Dec | 2.46 | 1.8 | 0.66 |
| Nelson | Growing Heifers - 1-2 | Jan | 6.3 | 4.61 | 1.69 |
| Nelson | Growing Heifers - 1-2 | Feb | 5.92 | 4.34 | 1.59 |
| Nelson | Growing Heifers - 1-2 | Mar | 7.86 | 5.75 | 2.1 |
| Nelson | Growing Heifers - 1-2 | Apr | 7.74 | 5.67 | 2.07 |
| Nelson | Growing Heifers - 1-2 | May | 6.54 | 4.79 | *1.75*† |
| Nelson | Growing Heifers - 1-2 | Jun | 6.54 | 4.79 | *1.75*† |
| Nelson | Growing Heifers - 1-2 | Jul | 5.16 | 3.78 | 1.38 |
| Nelson | Growing Heifers - 1-2 | Aug | 5.4 | 3.95 | 1.45 |
| Nelson | Growing Heifers - 1-2 | Sep | 5.46 | 4 | 1.46 |
| Nelson | Growing Heifers - 1-2 | Oct | 5.21 | 3.82 | 1.4 |
| Nelson | Growing Heifers - 1-2 | Nov | 5.38 | 3.94 | 1.44 |
| Nelson | Growing Heifers - 1-2 | Dec | 5.87 | 4.3 | 1.57 |
| Nelson | Breeding Bulls | Jan | 8.13 | 5.96 | 2.18 |
| Nelson | Breeding Bulls | Feb | 7.38 | 5.4 | 1.98 |
| Nelson | Breeding Bulls | Mar | 9.42 | 6.9 | 2.52 |
| Nelson | Breeding Bulls | Apr | 8.94 | 6.55 | 2.39 |
| Nelson | Breeding Bulls | May | 9.36 | 6.86 | 2.51 |
| Nelson | Breeding Bulls | Jun | 8.6 | 6.3 | 2.3 |
| Nelson | Breeding Bulls | Jul | 8.93 | 6.54 | 2.39 |
| Nelson | Breeding Bulls | Aug | 8.77 | 6.42 | 2.35 |
| Nelson | Breeding Bulls | Sep | 8.37 | 6.13 | 2.24 |
| Nelson | Breeding Bulls | Oct | 7.63 | 5.59 | 2.04 |
| Nelson | Breeding Bulls | Nov | 7.51 | 5.5 | 2.01 |
| Nelson | Breeding Bulls | Dec | 7.84 | 5.74 | 2.1 |
| Northland | Milking Cows - Mature | Jan | 10.42 | 7.63 | 2.79 |
| Northland | Milking Cows - Mature | Feb | 8.95 | 6.55 | 2.4 |
| Northland | Milking Cows - Mature | Mar | 10.85 | 7.95 | 2.91 |
| Northland | Milking Cows - Mature | Apr | 9.69 | 7.09 | 2.59 |
| Northland | Milking Cows - Mature | May | 8.61 | 6.3 | 2.3 |
| Northland | Milking Cows - Mature | Jun | 7.3 | 5.35 | 1.95 |
| Northland | Milking Cows - Mature | Jul | 9.31 | 6.82 | 2.49 |
| Northland | Milking Cows - Mature | Aug | 9.17 | 6.72 | 2.46 |
| Northland | Milking Cows - Mature | Sep | 12.7 | 9.3 | 3.4 |
| Northland | Milking Cows - Mature | Oct | 12.57 | 9.21 | 3.37 |
| Northland | Milking Cows - Mature | Nov | 11.76 | 8.61 | 3.15 |
| Northland | Milking Cows - Mature | Dec | 11.33 | 8.3 | 3.03 |
| Northland | Growing Heifers - 0-1 | Jan | 2.89 | 2.12 | 0.77 |
| Northland | Growing Heifers - 0-1 | Feb | 2.87 | 2.1 | 0.77 |
| Northland | Growing Heifers - 0-1 | Mar | 4.04 | 2.96 | 1.08 |
| Northland | Growing Heifers - 0-1 | Apr | 4.23 | 3.1 | 1.13 |
| Northland | Growing Heifers - 0-1 | May | 4.81 | 3.53 | 1.29 |
| Northland | Growing Heifers - 0-1 | Jun | 4.76 | 3.49 | 1.28 |
| Northland | Growing Heifers - 0-1 | Jul | 3.56 | 2.61 | *0.95** |
| Northland | Growing Heifers - 0-1 | Aug | 3.56 | 2.61 | *0.95** |
| Northland | Growing Heifers - 0-1 | Sep | 3.56 | 2.61 | *0.95** |
| Northland | Growing Heifers - 0-1 | Oct | 1.88 | 1.37 | 0.5 |
| Northland | Growing Heifers - 0-1 | Nov | 2.13 | 1.56 | 0.57 |
| Northland | Growing Heifers - 0-1 | Dec | 2.51 | 1.84 | 0.67 |
| Northland | Growing Heifers - 1-2 | Jan | 6.42 | 4.7 | 1.72 |
| Northland | Growing Heifers - 1-2 | Feb | 6.04 | 4.42 | 1.62 |
| Northland | Growing Heifers - 1-2 | Mar | 8.01 | 5.87 | 2.14 |
| Northland | Growing Heifers - 1-2 | Apr | 7.88 | 5.77 | 2.11 |
| Northland | Growing Heifers - 1-2 | May | 6.68 | 4.89 | *1.79*† |
| Northland | Growing Heifers - 1-2 | Jun | 6.68 | 4.89 | *1.79*† |
| Northland | Growing Heifers - 1-2 | Jul | 5.29 | 3.87 | 1.42 |
| Northland | Growing Heifers - 1-2 | Aug | 5.54 | 4.05 | 1.48 |
| Northland | Growing Heifers - 1-2 | Sep | 5.6 | 4.1 | 1.5 |
| Northland | Growing Heifers - 1-2 | Oct | 5.34 | 3.91 | 1.43 |
| Northland | Growing Heifers - 1-2 | Nov | 5.51 | 4.04 | 1.48 |
| Northland | Growing Heifers - 1-2 | Dec | 6.02 | 4.41 | 1.61 |
| Northland | Breeding Bulls | Jan | 8.13 | 5.96 | 2.18 |
| Northland | Breeding Bulls | Feb | 7.38 | 5.4 | 1.98 |
| Northland | Breeding Bulls | Mar | 9.42 | 6.9 | 2.52 |
| Northland | Breeding Bulls | Apr | 8.94 | 6.55 | 2.39 |
| Northland | Breeding Bulls | May | 9.36 | 6.86 | 2.51 |
| Northland | Breeding Bulls | Jun | 8.6 | 6.3 | 2.3 |
| Northland | Breeding Bulls | Jul | 8.93 | 6.54 | 2.39 |
| Northland | Breeding Bulls | Aug | 8.77 | 6.42 | 2.35 |
| Northland | Breeding Bulls | Sep | 8.37 | 6.13 | 2.24 |
| Northland | Breeding Bulls | Oct | 7.63 | 5.59 | 2.04 |
| Northland | Breeding Bulls | Nov | 7.51 | 5.5 | 2.01 |
| Northland | Breeding Bulls | Dec | 7.84 | 5.74 | 2.1 |
| Otago | Milking Cows - Mature | Jan | 11.59 | 8.48 | 3.1 |
| Otago | Milking Cows - Mature | Feb | 9.89 | 7.25 | 2.65 |
| Otago | Milking Cows - Mature | Mar | 11.95 | 8.75 | 3.2 |
| Otago | Milking Cows - Mature | Apr | 10.52 | 7.71 | 2.82 |
| Otago | Milking Cows - Mature | May | 9.16 | 6.71 | 2.45 |
| Otago | Milking Cows - Mature | Jun | 7.43 | 5.44 | 1.99 |
| Otago | Milking Cows - Mature | Jul | 9.39 | 6.88 | 2.52 |
| Otago | Milking Cows - Mature | Aug | 9.59 | 7.02 | 2.57 |
| Otago | Milking Cows - Mature | Sep | 13.53 | 9.91 | 3.62 |
| Otago | Milking Cows - Mature | Oct | 13.43 | 9.83 | 3.6 |
| Otago | Milking Cows - Mature | Nov | 12.57 | 9.2 | 3.37 |
| Otago | Milking Cows - Mature | Dec | 12.07 | 8.84 | 3.23 |
| Otago | Growing Heifers - 0-1 | Jan | 2.92 | 2.14 | 0.78 |
| Otago | Growing Heifers - 0-1 | Feb | 2.89 | 2.12 | 0.77 |
| Otago | Growing Heifers - 0-1 | Mar | 4.08 | 2.99 | 1.09 |
| Otago | Growing Heifers - 0-1 | Apr | 4.27 | 3.13 | 1.14 |
| Otago | Growing Heifers - 0-1 | May | 4.86 | 3.56 | 1.3 |
| Otago | Growing Heifers - 0-1 | Jun | 4.81 | 3.52 | 1.29 |
| Otago | Growing Heifers - 0-1 | Jul | 3.59 | 2.63 | *0.96** |
| Otago | Growing Heifers - 0-1 | Aug | 3.59 | 2.63 | *0.96** |
| Otago | Growing Heifers - 0-1 | Sep | 3.59 | 2.63 | *0.96** |
| Otago | Growing Heifers - 0-1 | Oct | 1.89 | 1.38 | 0.5 |
| Otago | Growing Heifers - 0-1 | Nov | 2.14 | 1.57 | 0.57 |
| Otago | Growing Heifers - 0-1 | Dec | 2.53 | 1.85 | 0.68 |
| Otago | Growing Heifers - 1-2 | Jan | 6.48 | 4.75 | 1.74 |
| Otago | Growing Heifers - 1-2 | Feb | 6.09 | 4.46 | 1.63 |
| Otago | Growing Heifers - 1-2 | Mar | 8.08 | 5.92 | 2.16 |
| Otago | Growing Heifers - 1-2 | Apr | 7.95 | 5.82 | 2.13 |
| Otago | Growing Heifers - 1-2 | May | 6.73 | 4.93 | *1.8*† |
| Otago | Growing Heifers - 1-2 | Jun | 6.73 | 4.93 | *1.8*† |
| Otago | Growing Heifers - 1-2 | Jul | 5.32 | 3.9 | 1.43 |
| Otago | Growing Heifers - 1-2 | Aug | 5.57 | 4.08 | 1.49 |
| Otago | Growing Heifers - 1-2 | Sep | 5.63 | 4.13 | 1.51 |
| Otago | Growing Heifers - 1-2 | Oct | 5.38 | 3.94 | 1.44 |
| Otago | Growing Heifers - 1-2 | Nov | 5.55 | 4.06 | 1.49 |
| Otago | Growing Heifers - 1-2 | Dec | 6.05 | 4.43 | 1.62 |
| Otago | Breeding Bulls | Jan | 8.13 | 5.96 | 2.18 |
| Otago | Breeding Bulls | Feb | 7.38 | 5.4 | 1.98 |
| Otago | Breeding Bulls | Mar | 9.42 | 6.9 | 2.52 |
| Otago | Breeding Bulls | Apr | 8.94 | 6.55 | 2.39 |
| Otago | Breeding Bulls | May | 9.36 | 6.86 | 2.51 |
| Otago | Breeding Bulls | Jun | 8.6 | 6.3 | 2.3 |
| Otago | Breeding Bulls | Jul | 8.93 | 6.54 | 2.39 |
| Otago | Breeding Bulls | Aug | 8.77 | 6.42 | 2.35 |
| Otago | Breeding Bulls | Sep | 8.37 | 6.13 | 2.24 |
| Otago | Breeding Bulls | Oct | 7.63 | 5.59 | 2.04 |
| Otago | Breeding Bulls | Nov | 7.51 | 5.5 | 2.01 |
| Otago | Breeding Bulls | Dec | 7.84 | 5.74 | 2.1 |
| Southland | Milking Cows - Mature | Jan | 13.56 | 9.93 | 3.63 |
| Southland | Milking Cows - Mature | Feb | 11.5 | 8.42 | 3.08 |
| Southland | Milking Cows - Mature | Mar | 13.77 | 10.08 | 3.69 |
| Southland | Milking Cows - Mature | Apr | 11.91 | 8.72 | 3.19 |
| Southland | Milking Cows - Mature | May | 9.97 | 7.3 | 2.67 |
| Southland | Milking Cows - Mature | Jun | 7.57 | 5.54 | 2.03 |
| Southland | Milking Cows - Mature | Jul | 9.53 | 6.98 | 2.55 |
| Southland | Milking Cows - Mature | Aug | 10.47 | 7.67 | 2.8 |
| Southland | Milking Cows - Mature | Sep | 15.36 | 11.25 | 4.11 |
| Southland | Milking Cows - Mature | Oct | 15.33 | 11.22 | 4.1 |
| Southland | Milking Cows - Mature | Nov | 14.37 | 10.52 | 3.85 |
| Southland | Milking Cows - Mature | Dec | 13.7 | 10.03 | 3.67 |
| Southland | Growing Heifers - 0-1 | Jan | 2.92 | 2.14 | 0.78 |
| Southland | Growing Heifers - 0-1 | Feb | 2.89 | 2.12 | 0.77 |
| Southland | Growing Heifers - 0-1 | Mar | 4.08 | 2.99 | 1.09 |
| Southland | Growing Heifers - 0-1 | Apr | 4.27 | 3.13 | 1.14 |
| Southland | Growing Heifers - 0-1 | May | 4.86 | 3.56 | 1.3 |
| Southland | Growing Heifers - 0-1 | Jun | 4.81 | 3.52 | 1.29 |
| Southland | Growing Heifers - 0-1 | Jul | 3.59 | 2.63 | *0.96** |
| Southland | Growing Heifers - 0-1 | Aug | 3.59 | 2.63 | *0.96** |
| Southland | Growing Heifers - 0-1 | Sep | 3.59 | 2.63 | *0.96** |
| Southland | Growing Heifers - 0-1 | Oct | 1.89 | 1.38 | 0.5 |
| Southland | Growing Heifers - 0-1 | Nov | 2.14 | 1.57 | 0.57 |
| Southland | Growing Heifers - 0-1 | Dec | 2.53 | 1.85 | 0.68 |
| Southland | Growing Heifers - 1-2 | Jan | 6.48 | 4.75 | 1.74 |
| Southland | Growing Heifers - 1-2 | Feb | 6.09 | 4.46 | 1.63 |
| Southland | Growing Heifers - 1-2 | Mar | 8.08 | 5.92 | 2.16 |
| Southland | Growing Heifers - 1-2 | Apr | 7.95 | 5.82 | 2.13 |
| Southland | Growing Heifers - 1-2 | May | 6.73 | 4.93 | *1.8*† |
| Southland | Growing Heifers - 1-2 | Jun | 6.73 | 4.93 | *1.8*† |
| Southland | Growing Heifers - 1-2 | Jul | 5.32 | 3.9 | 1.43 |
| Southland | Growing Heifers - 1-2 | Aug | 5.57 | 4.08 | 1.49 |
| Southland | Growing Heifers - 1-2 | Sep | 5.63 | 4.13 | 1.51 |
| Southland | Growing Heifers - 1-2 | Oct | 5.38 | 3.94 | 1.44 |
| Southland | Growing Heifers - 1-2 | Nov | 5.55 | 4.06 | 1.49 |
| Southland | Growing Heifers - 1-2 | Dec | 6.05 | 4.43 | 1.62 |
| Southland | Breeding Bulls | Jan | 8.13 | 5.96 | 2.18 |
| Southland | Breeding Bulls | Feb | 7.38 | 5.4 | 1.98 |
| Southland | Breeding Bulls | Mar | 9.42 | 6.9 | 2.52 |
| Southland | Breeding Bulls | Apr | 8.94 | 6.55 | 2.39 |
| Southland | Breeding Bulls | May | 9.36 | 6.86 | 2.51 |
| Southland | Breeding Bulls | Jun | 8.6 | 6.3 | 2.3 |
| Southland | Breeding Bulls | Jul | 8.93 | 6.54 | 2.39 |
| Southland | Breeding Bulls | Aug | 8.77 | 6.42 | 2.35 |
| Southland | Breeding Bulls | Sep | 8.37 | 6.13 | 2.24 |
| Southland | Breeding Bulls | Oct | 7.63 | 5.59 | 2.04 |
| Southland | Breeding Bulls | Nov | 7.51 | 5.5 | 2.01 |
| Southland | Breeding Bulls | Dec | 7.84 | 5.74 | 2.1 |
| Taranaki | Milking Cows - Mature | Jan | 11.54 | 8.45 | 3.09 |
| Taranaki | Milking Cows - Mature | Feb | 9.86 | 7.22 | 2.64 |
| Taranaki | Milking Cows - Mature | Mar | 11.89 | 8.7 | 3.18 |
| Taranaki | Milking Cows - Mature | Apr | 10.47 | 7.67 | 2.8 |
| Taranaki | Milking Cows - Mature | May | 8.98 | 6.57 | 2.4 |
| Taranaki | Milking Cows - Mature | Jun | 7.35 | 5.38 | 1.97 |
| Taranaki | Milking Cows - Mature | Jul | 9.45 | 6.92 | 2.53 |
| Taranaki | Milking Cows - Mature | Aug | 10.21 | 7.48 | 2.73 |
| Taranaki | Milking Cows - Mature | Sep | 14.88 | 10.9 | 3.98 |
| Taranaki | Milking Cows - Mature | Oct | 14.85 | 10.87 | 3.97 |
| Taranaki | Milking Cows - Mature | Nov | 13.91 | 10.18 | 3.72 |
| Taranaki | Milking Cows - Mature | Dec | 13.28 | 9.72 | 3.55 |
| Taranaki | Growing Heifers - 0-1 | Jan | 2.88 | 2.11 | 0.77 |
| Taranaki | Growing Heifers - 0-1 | Feb | 2.86 | 2.09 | 0.76 |
| Taranaki | Growing Heifers - 0-1 | Mar | 4.03 | 2.95 | 1.08 |
| Taranaki | Growing Heifers - 0-1 | Apr | 4.21 | 3.08 | 1.13 |
| Taranaki | Growing Heifers - 0-1 | May | 4.8 | 3.51 | 1.28 |
| Taranaki | Growing Heifers - 0-1 | Jun | 4.75 | 3.48 | 1.27 |
| Taranaki | Growing Heifers - 0-1 | Jul | 3.55 | 2.6 | *0.95** |
| Taranaki | Growing Heifers - 0-1 | Aug | 3.55 | 2.6 | *0.95** |
| Taranaki | Growing Heifers - 0-1 | Sep | 3.55 | 2.6 | *0.95** |
| Taranaki | Growing Heifers - 0-1 | Oct | 1.87 | 1.37 | 0.5 |
| Taranaki | Growing Heifers - 0-1 | Nov | 2.11 | 1.55 | 0.57 |
| Taranaki | Growing Heifers - 0-1 | Dec | 2.5 | 1.83 | 0.67 |
| Taranaki | Growing Heifers - 1-2 | Jan | 6.4 | 4.69 | 1.71 |
| Taranaki | Growing Heifers - 1-2 | Feb | 6.02 | 4.41 | 1.61 |
| Taranaki | Growing Heifers - 1-2 | Mar | 7.98 | 5.84 | 2.14 |
| Taranaki | Growing Heifers - 1-2 | Apr | 7.85 | 5.75 | 2.1 |
| Taranaki | Growing Heifers - 1-2 | May | 6.65 | 4.87 | *1.78*† |
| Taranaki | Growing Heifers - 1-2 | Jun | 6.65 | 4.87 | *1.78*† |
| Taranaki | Growing Heifers - 1-2 | Jul | 5.26 | 3.85 | 1.41 |
| Taranaki | Growing Heifers - 1-2 | Aug | 5.5 | 4.03 | 1.47 |
| Taranaki | Growing Heifers - 1-2 | Sep | 5.57 | 4.08 | 1.49 |
| Taranaki | Growing Heifers - 1-2 | Oct | 5.31 | 3.89 | 1.42 |
| Taranaki | Growing Heifers - 1-2 | Nov | 5.48 | 4.01 | 1.47 |
| Taranaki | Growing Heifers - 1-2 | Dec | 5.98 | 4.38 | 1.6 |
| Taranaki | Breeding Bulls | Jan | 8.13 | 5.96 | 2.18 |
| Taranaki | Breeding Bulls | Feb | 7.38 | 5.4 | 1.98 |
| Taranaki | Breeding Bulls | Mar | 9.42 | 6.9 | 2.52 |
| Taranaki | Breeding Bulls | Apr | 8.94 | 6.55 | 2.39 |
| Taranaki | Breeding Bulls | May | 9.36 | 6.86 | 2.51 |
| Taranaki | Breeding Bulls | Jun | 8.6 | 6.3 | 2.3 |
| Taranaki | Breeding Bulls | Jul | 8.93 | 6.54 | 2.39 |
| Taranaki | Breeding Bulls | Aug | 8.77 | 6.42 | 2.35 |
| Taranaki | Breeding Bulls | Sep | 8.37 | 6.13 | 2.24 |
| Taranaki | Breeding Bulls | Oct | 7.63 | 5.59 | 2.04 |
| Taranaki | Breeding Bulls | Nov | 7.51 | 5.5 | 2.01 |
| Taranaki | Breeding Bulls | Dec | 7.84 | 5.74 | 2.1 |
| Tasman | Milking Cows - Mature | Jan | 11.39 | 8.34 | 3.05 |
| Tasman | Milking Cows - Mature | Feb | 9.72 | 7.12 | 2.6 |
| Tasman | Milking Cows - Mature | Mar | 11.72 | 8.58 | 3.14 |
| Tasman | Milking Cows - Mature | Apr | 10.34 | 7.57 | 2.77 |
| Tasman | Milking Cows - Mature | May | 8.9 | 6.51 | 2.38 |
| Tasman | Milking Cows - Mature | Jun | 7.29 | 5.34 | 1.95 |
| Tasman | Milking Cows - Mature | Jul | 9.3 | 6.81 | 2.49 |
| Tasman | Milking Cows - Mature | Aug | 9.66 | 7.08 | 2.59 |
| Tasman | Milking Cows - Mature | Sep | 13.82 | 10.12 | 3.7 |
| Tasman | Milking Cows - Mature | Oct | 13.76 | 10.08 | 3.68 |
| Tasman | Milking Cows - Mature | Nov | 12.86 | 9.42 | 3.44 |
| Tasman | Milking Cows - Mature | Dec | 12.32 | 9.02 | 3.3 |
| Tasman | Growing Heifers - 0-1 | Jan | 2.83 | 2.08 | 0.76 |
| Tasman | Growing Heifers - 0-1 | Feb | 2.81 | 2.06 | 0.75 |
| Tasman | Growing Heifers - 0-1 | Mar | 3.96 | 2.9 | 1.06 |
| Tasman | Growing Heifers - 0-1 | Apr | 4.14 | 3.03 | 1.11 |
| Tasman | Growing Heifers - 0-1 | May | 4.72 | 3.45 | 1.26 |
| Tasman | Growing Heifers - 0-1 | Jun | 4.67 | 3.42 | 1.25 |
| Tasman | Growing Heifers - 0-1 | Jul | 3.49 | 2.55 | *0.93** |
| Tasman | Growing Heifers - 0-1 | Aug | 3.49 | 2.55 | *0.93** |
| Tasman | Growing Heifers - 0-1 | Sep | 3.49 | 2.55 | *0.93** |
| Tasman | Growing Heifers - 0-1 | Oct | 1.83 | 1.34 | 0.49 |
| Tasman | Growing Heifers - 0-1 | Nov | 2.08 | 1.52 | 0.56 |
| Tasman | Growing Heifers - 0-1 | Dec | 2.46 | 1.8 | 0.66 |
| Tasman | Growing Heifers - 1-2 | Jan | 6.3 | 4.61 | 1.69 |
| Tasman | Growing Heifers - 1-2 | Feb | 5.92 | 4.34 | 1.59 |
| Tasman | Growing Heifers - 1-2 | Mar | 7.86 | 5.75 | 2.1 |
| Tasman | Growing Heifers - 1-2 | Apr | 7.74 | 5.67 | 2.07 |
| Tasman | Growing Heifers - 1-2 | May | 6.54 | 4.79 | *1.75*† |
| Tasman | Growing Heifers - 1-2 | Jun | 6.54 | 4.79 | *1.75*† |
| Tasman | Growing Heifers - 1-2 | Jul | 5.16 | 3.78 | 1.38 |
| Tasman | Growing Heifers - 1-2 | Aug | 5.4 | 3.95 | 1.45 |
| Tasman | Growing Heifers - 1-2 | Sep | 5.46 | 4 | 1.46 |
| Tasman | Growing Heifers - 1-2 | Oct | 5.21 | 3.82 | 1.4 |
| Tasman | Growing Heifers - 1-2 | Nov | 5.38 | 3.94 | 1.44 |
| Tasman | Growing Heifers - 1-2 | Dec | 5.87 | 4.3 | 1.57 |
| Tasman | Breeding Bulls | Jan | 8.13 | 5.96 | 2.18 |
| Tasman | Breeding Bulls | Feb | 7.38 | 5.4 | 1.98 |
| Tasman | Breeding Bulls | Mar | 9.42 | 6.9 | 2.52 |
| Tasman | Breeding Bulls | Apr | 8.94 | 6.55 | 2.39 |
| Tasman | Breeding Bulls | May | 9.36 | 6.86 | 2.51 |
| Tasman | Breeding Bulls | Jun | 8.6 | 6.3 | 2.3 |
| Tasman | Breeding Bulls | Jul | 8.93 | 6.54 | 2.39 |
| Tasman | Breeding Bulls | Aug | 8.77 | 6.42 | 2.35 |
| Tasman | Breeding Bulls | Sep | 8.37 | 6.13 | 2.24 |
| Tasman | Breeding Bulls | Oct | 7.63 | 5.59 | 2.04 |
| Tasman | Breeding Bulls | Nov | 7.51 | 5.5 | 2.01 |
| Tasman | Breeding Bulls | Dec | 7.84 | 5.74 | 2.1 |
| Waikato | Milking Cows - Mature | Jan | 10.81 | 7.92 | 2.89 |
| Waikato | Milking Cows - Mature | Feb | 9.27 | 6.79 | 2.48 |
| Waikato | Milking Cows - Mature | Mar | 11.21 | 8.21 | 3 |
| Waikato | Milking Cows - Mature | Apr | 9.96 | 7.29 | 2.67 |
| Waikato | Milking Cows - Mature | May | 8.79 | 6.43 | 2.35 |
| Waikato | Milking Cows - Mature | Jun | 7.33 | 5.37 | 1.96 |
| Waikato | Milking Cows - Mature | Jul | 9.33 | 6.83 | 2.5 |
| Waikato | Milking Cows - Mature | Aug | 9.42 | 6.9 | 2.52 |
| Waikato | Milking Cows - Mature | Sep | 13.23 | 9.69 | 3.54 |
| Waikato | Milking Cows - Mature | Oct | 13.13 | 9.62 | 3.52 |
| Waikato | Milking Cows - Mature | Nov | 12.29 | 9 | 3.29 |
| Waikato | Milking Cows - Mature | Dec | 11.8 | 8.64 | 3.16 |
| Waikato | Growing Heifers - 0-1 | Jan | 2.89 | 2.12 | 0.77 |
| Waikato | Growing Heifers - 0-1 | Feb | 2.86 | 2.1 | 0.77 |
| Waikato | Growing Heifers - 0-1 | Mar | 4.04 | 2.96 | 1.08 |
| Waikato | Growing Heifers - 0-1 | Apr | 4.23 | 3.09 | 1.13 |
| Waikato | Growing Heifers - 0-1 | May | 4.81 | 3.52 | 1.29 |
| Waikato | Growing Heifers - 0-1 | Jun | 4.76 | 3.49 | 1.27 |
| Waikato | Growing Heifers - 0-1 | Jul | 3.56 | 2.6 | *0.95** |
| Waikato | Growing Heifers - 0-1 | Aug | 3.56 | 2.6 | *0.95** |
| Waikato | Growing Heifers - 0-1 | Sep | 3.56 | 2.6 | *0.95** |
| Waikato | Growing Heifers - 0-1 | Oct | 1.87 | 1.37 | 0.5 |
| Waikato | Growing Heifers - 0-1 | Nov | 2.12 | 1.55 | 0.57 |
| Waikato | Growing Heifers - 0-1 | Dec | 2.5 | 1.83 | 0.67 |
| Waikato | Growing Heifers - 1-2 | Jan | 6.42 | 4.7 | 1.72 |
| Waikato | Growing Heifers - 1-2 | Feb | 6.04 | 4.42 | 1.62 |
| Waikato | Growing Heifers - 1-2 | Mar | 8.01 | 5.86 | 2.14 |
| Waikato | Growing Heifers - 1-2 | Apr | 7.88 | 5.77 | 2.11 |
| Waikato | Growing Heifers - 1-2 | May | 6.67 | 4.88 | *1.79*† |
| Waikato | Growing Heifers - 1-2 | Jun | 6.67 | 4.88 | *1.79*† |
| Waikato | Growing Heifers - 1-2 | Jul | 5.27 | 3.86 | 1.41 |
| Waikato | Growing Heifers - 1-2 | Aug | 5.52 | 4.04 | 1.48 |
| Waikato | Growing Heifers - 1-2 | Sep | 5.58 | 4.08 | 1.49 |
| Waikato | Growing Heifers - 1-2 | Oct | 5.32 | 3.9 | 1.43 |
| Waikato | Growing Heifers - 1-2 | Nov | 5.49 | 4.02 | 1.47 |
| Waikato | Growing Heifers - 1-2 | Dec | 6 | 4.39 | 1.61 |
| Waikato | Breeding Bulls | Jan | 8.13 | 5.96 | 2.18 |
| Waikato | Breeding Bulls | Feb | 7.38 | 5.4 | 1.98 |
| Waikato | Breeding Bulls | Mar | 9.42 | 6.9 | 2.52 |
| Waikato | Breeding Bulls | Apr | 8.94 | 6.55 | 2.39 |
| Waikato | Breeding Bulls | May | 9.36 | 6.86 | 2.51 |
| Waikato | Breeding Bulls | Jun | 8.6 | 6.3 | 2.3 |
| Waikato | Breeding Bulls | Jul | 8.93 | 6.54 | 2.39 |
| Waikato | Breeding Bulls | Aug | 8.77 | 6.42 | 2.35 |
| Waikato | Breeding Bulls | Sep | 8.37 | 6.13 | 2.24 |
| Waikato | Breeding Bulls | Oct | 7.63 | 5.59 | 2.04 |
| Waikato | Breeding Bulls | Nov | 7.51 | 5.5 | 2.01 |
| Waikato | Breeding Bulls | Dec | 7.84 | 5.74 | 2.1 |
| Wellington | Milking Cows - Mature | Jan | 11.88 | 8.7 | 3.18 |
| Wellington | Milking Cows - Mature | Feb | 10.14 | 7.42 | 2.71 |
| Wellington | Milking Cows - Mature | Mar | 12.21 | 8.94 | 3.27 |
| Wellington | Milking Cows - Mature | Apr | 10.73 | 7.86 | 2.87 |
| Wellington | Milking Cows - Mature | May | 9.41 | 6.89 | 2.52 |
| Wellington | Milking Cows - Mature | Jun | 7.47 | 5.47 | 2 |
| Wellington | Milking Cows - Mature | Jul | 9.33 | 6.83 | 2.5 |
| Wellington | Milking Cows - Mature | Aug | 9.15 | 6.7 | 2.45 |
| Wellington | Milking Cows - Mature | Sep | 12.61 | 9.24 | 3.38 |
| Wellington | Milking Cows - Mature | Oct | 12.48 | 9.14 | 3.34 |
| Wellington | Milking Cows - Mature | Nov | 11.67 | 8.54 | 3.12 |
| Wellington | Milking Cows - Mature | Dec | 11.25 | 8.24 | 3.01 |
| Wellington | Growing Heifers - 0-1 | Jan | 2.91 | 2.13 | 0.78 |
| Wellington | Growing Heifers - 0-1 | Feb | 2.89 | 2.12 | 0.77 |
| Wellington | Growing Heifers - 0-1 | Mar | 4.08 | 2.99 | 1.09 |
| Wellington | Growing Heifers - 0-1 | Apr | 4.26 | 3.12 | 1.14 |
| Wellington | Growing Heifers - 0-1 | May | 4.86 | 3.56 | 1.3 |
| Wellington | Growing Heifers - 0-1 | Jun | 4.81 | 3.52 | 1.29 |
| Wellington | Growing Heifers - 0-1 | Jul | 3.59 | 2.63 | *0.96** |
| Wellington | Growing Heifers - 0-1 | Aug | 3.59 | 2.63 | *0.96** |
| Wellington | Growing Heifers - 0-1 | Sep | 3.59 | 2.63 | *0.96** |
| Wellington | Growing Heifers - 0-1 | Oct | 1.89 | 1.38 | 0.51 |
| Wellington | Growing Heifers - 0-1 | Nov | 2.14 | 1.57 | 0.57 |
| Wellington | Growing Heifers - 0-1 | Dec | 2.54 | 1.86 | 0.68 |
| Wellington | Growing Heifers - 1-2 | Jan | 6.48 | 4.74 | 1.73 |
| Wellington | Growing Heifers - 1-2 | Feb | 6.09 | 4.46 | 1.63 |
| Wellington | Growing Heifers - 1-2 | Mar | 8.08 | 5.91 | 2.16 |
| Wellington | Growing Heifers - 1-2 | Apr | 7.94 | 5.82 | 2.13 |
| Wellington | Growing Heifers - 1-2 | May | 6.74 | 4.93 | *1.81*† |
| Wellington | Growing Heifers - 1-2 | Jun | 6.74 | 4.93 | *1.81*† |
| Wellington | Growing Heifers - 1-2 | Jul | 5.34 | 3.91 | 1.43 |
| Wellington | Growing Heifers - 1-2 | Aug | 5.59 | 4.09 | 1.5 |
| Wellington | Growing Heifers - 1-2 | Sep | 5.65 | 4.14 | 1.51 |
| Wellington | Growing Heifers - 1-2 | Oct | 5.39 | 3.95 | 1.44 |
| Wellington | Growing Heifers - 1-2 | Nov | 5.56 | 4.07 | 1.49 |
| Wellington | Growing Heifers - 1-2 | Dec | 6.07 | 4.45 | 1.63 |
| Wellington | Breeding Bulls | Jan | 8.13 | 5.96 | 2.18 |
| Wellington | Breeding Bulls | Feb | 7.38 | 5.4 | 1.98 |
| Wellington | Breeding Bulls | Mar | 9.42 | 6.9 | 2.52 |
| Wellington | Breeding Bulls | Apr | 8.94 | 6.55 | 2.39 |
| Wellington | Breeding Bulls | May | 9.36 | 6.86 | 2.51 |
| Wellington | Breeding Bulls | Jun | 8.6 | 6.3 | 2.3 |
| Wellington | Breeding Bulls | Jul | 8.93 | 6.54 | 2.39 |
| Wellington | Breeding Bulls | Aug | 8.77 | 6.42 | 2.35 |
| Wellington | Breeding Bulls | Sep | 8.37 | 6.13 | 2.24 |
| Wellington | Breeding Bulls | Oct | 7.63 | 5.59 | 2.04 |
| Wellington | Breeding Bulls | Nov | 7.51 | 5.5 | 2.01 |
| Wellington | Breeding Bulls | Dec | 7.84 | 5.74 | 2.1 |
| West Coast | Milking Cows - Mature | Jan | 12.01 | 8.79 | 3.21 |
| West Coast | Milking Cows - Mature | Feb | 10.22 | 7.48 | 2.74 |
| West Coast | Milking Cows - Mature | Mar | 12.3 | 9 | 3.29 |
| West Coast | Milking Cows - Mature | Apr | 10.77 | 7.89 | 2.88 |
| West Coast | Milking Cows - Mature | May | 9.29 | 6.81 | 2.49 |
| West Coast | Milking Cows - Mature | Jun | 7.36 | 5.39 | 1.97 |
| West Coast | Milking Cows - Mature | Jul | 9.26 | 6.78 | 2.48 |
| West Coast | Milking Cows - Mature | Aug | 9.44 | 6.91 | 2.53 |
| West Coast | Milking Cows - Mature | Sep | 13.35 | 9.78 | 3.58 |
| West Coast | Milking Cows - Mature | Oct | 13.27 | 9.72 | 3.55 |
| West Coast | Milking Cows - Mature | Nov | 12.4 | 9.08 | 3.32 |
| West Coast | Milking Cows - Mature | Dec | 11.89 | 8.71 | 3.18 |
| West Coast | Growing Heifers - 0-1 | Jan | 2.83 | 2.08 | 0.76 |
| West Coast | Growing Heifers - 0-1 | Feb | 2.81 | 2.06 | 0.75 |
| West Coast | Growing Heifers - 0-1 | Mar | 3.96 | 2.9 | 1.06 |
| West Coast | Growing Heifers - 0-1 | Apr | 4.14 | 3.03 | 1.11 |
| West Coast | Growing Heifers - 0-1 | May | 4.72 | 3.45 | 1.26 |
| West Coast | Growing Heifers - 0-1 | Jun | 4.67 | 3.42 | 1.25 |
| West Coast | Growing Heifers - 0-1 | Jul | 3.49 | 2.55 | *0.93** |
| West Coast | Growing Heifers - 0-1 | Aug | 3.49 | 2.55 | *0.93** |
| West Coast | Growing Heifers - 0-1 | Sep | 3.49 | 2.55 | *0.93** |
| West Coast | Growing Heifers - 0-1 | Oct | 1.83 | 1.34 | 0.49 |
| West Coast | Growing Heifers - 0-1 | Nov | 2.08 | 1.52 | 0.56 |
| West Coast | Growing Heifers - 0-1 | Dec | 2.46 | 1.8 | 0.66 |
| West Coast | Growing Heifers - 1-2 | Jan | 6.3 | 4.61 | 1.69 |
| West Coast | Growing Heifers - 1-2 | Feb | 5.92 | 4.34 | 1.59 |
| West Coast | Growing Heifers - 1-2 | Mar | 7.86 | 5.75 | 2.1 |
| West Coast | Growing Heifers - 1-2 | Apr | 7.74 | 5.67 | 2.07 |
| West Coast | Growing Heifers - 1-2 | May | 6.54 | 4.79 | *1.75*† |
| West Coast | Growing Heifers - 1-2 | Jun | 6.54 | 4.79 | *1.75*† |
| West Coast | Growing Heifers - 1-2 | Jul | 5.16 | 3.78 | 1.38 |
| West Coast | Growing Heifers - 1-2 | Aug | 5.4 | 3.95 | 1.45 |
| West Coast | Growing Heifers - 1-2 | Sep | 5.46 | 4 | 1.46 |
| West Coast | Growing Heifers - 1-2 | Oct | 5.21 | 3.82 | 1.4 |
| West Coast | Growing Heifers - 1-2 | Nov | 5.38 | 3.94 | 1.44 |
| West Coast | Growing Heifers - 1-2 | Dec | 5.87 | 4.3 | 1.57 |
| West Coast | Breeding Bulls | Jan | 8.13 | 5.96 | 2.18 |
| West Coast | Breeding Bulls | Feb | 7.38 | 5.4 | 1.98 |
| West Coast | Breeding Bulls | Mar | 9.42 | 6.9 | 2.52 |
| West Coast | Breeding Bulls | Apr | 8.94 | 6.55 | 2.39 |
| West Coast | Breeding Bulls | May | 9.36 | 6.86 | 2.51 |
| West Coast | Breeding Bulls | Jun | 8.6 | 6.3 | 2.3 |
| West Coast | Breeding Bulls | Jul | 8.93 | 6.54 | 2.39 |
| West Coast | Breeding Bulls | Aug | 8.77 | 6.42 | 2.35 |
| West Coast | Breeding Bulls | Sep | 8.37 | 6.13 | 2.24 |
| West Coast | Breeding Bulls | Oct | 7.63 | 5.59 | 2.04 |
| West Coast | Breeding Bulls | Nov | 7.51 | 5.5 | 2.01 |
| West Coast | Breeding Bulls | Dec | 7.84 | 5.74 | 2.1 |

* Applied an average of Apr-Jun and Oct-Nov.

† Applied an average of Mar-Apr and Jul-Aug.

## Beef cattle

**Table S2**. N excretion rates for different age classes of beef cattle (2021-2022), sourced from Pickering, Gibbs [3]. Note that estimated values are in italics with the method indicated in a footnote to the Table.

| Class | Month | Total Excreta kg N/head.month | Nitrogen Excreted In Urine kg N/head | Nitrogen Excreted In Faeces kg N/head |
| --- | --- | --- | --- | --- |
| Breeding Growing Cows 0-1 | Jan | 1.11 | 0.83 | 0.28 |
| Breeding Growing Cows 0-1 | Feb | 1.24 | 0.93 | 0.31 |
| Breeding Growing Cows 0-1 | Mar | 3.39 | 2.54 | 0.85 |
| Breeding Growing Cows 0-1 | Apr | 3.56 | 2.67 | 0.89 |
| Breeding Growing Cows 0-1 | May | 4.12 | 3.08 | 1.03 |
| Breeding Growing Cows 0-1 | Jun | 4 | 3 | 1.01 |
| Breeding Growing Cows 0-1 | Jul | 4.34 | 3.25 | 1.09 |
| Breeding Growing Cows 0-1 | Aug | 4.53 | 3.39 | 1.14 |
| Breeding Growing Cows 0-1 | Sep | 5 | 3.74 | *1.26‡* |
| Breeding Growing Cows 0-1 | Oct | 5 | 3.74 | *1.26‡* |
| Breeding Growing Cows 0-1 | Nov | 5 | 3.74 | *1.26‡* |
| Breeding Growing Cows 0-1 | Dec | 5 | 3.74 | *1.26‡* |
| Breeding Growing Cows 1-2 | Jan | 5.3 | 3.97 | 1.33 |
| Breeding Growing Cows 1-2 | Feb | 5.07 | 3.8 | 1.27 |
| Breeding Growing Cows 1-2 | Mar | 6.73 | 5.04 | 1.69 |
| Breeding Growing Cows 1-2 | Apr | 6.71 | 5.03 | 1.69 |
| Breeding Growing Cows 1-2 | May | 7.43 | 5.56 | 1.86 |
| Breeding Growing Cows 1-2 | Jun | 6.93 | 5.19 | 1.74 |
| Breeding Growing Cows 1-2 | Jul | 7.25 | 5.43 | 1.82 |
| Breeding Growing Cows 1-2 | Aug | 7.31 | 5.48 | 1.84 |
| Breeding Growing Cows 1-2 | Sep | 4.49 | 3.36 | 1.13 |
| Breeding Growing Cows 1-2 | Oct | 4.22 | 3.16 | 1.06 |
| Breeding Growing Cows 1-2 | Nov | 4.33 | 3.24 | 1.09 |
| Breeding Growing Cows 1-2 | Dec | 4.73 | 3.55 | 1.19 |
| Breeding Bulls Mixed Age | Jan | 10.17 | 7.62 | 2.55 |
| Breeding Bulls Mixed Age | Feb | 9.47 | 7.1 | 2.38 |
| Breeding Bulls Mixed Age | Mar | 12.12 | 9.08 | 3.04 |
| Breeding Bulls Mixed Age | Apr | 11.39 | 8.53 | 2.86 |
| Breeding Bulls Mixed Age | May | 12.07 | 9.04 | 3.03 |
| Breeding Bulls Mixed Age | Jun | 10.79 | 8.08 | 2.71 |
| Breeding Bulls Mixed Age | Jul | 11.19 | 8.38 | 2.81 |
| Breeding Bulls Mixed Age | Aug | 10.89 | 8.16 | 2.74 |
| Breeding Bulls Mixed Age | Sep | 10.36 | 7.76 | 2.6 |
| Breeding Bulls Mixed Age | Oct | 9.31 | 6.98 | 2.34 |
| Breeding Bulls Mixed Age | Nov | 9.28 | 6.95 | 2.33 |
| Breeding Bulls Mixed Age | Dec | 9.74 | 7.29 | 2.44 |
| Slaughter Heifers 0-1 | Jan | 1.62 | 1.21 | 0.41 |
| Slaughter Heifers 0-1 | Feb | 1.8 | 1.35 | 0.45 |
| Slaughter Heifers 0-1 | Mar | 4.22 | 3.16 | 1.06 |
| Slaughter Heifers 0-1 | Apr | 4.46 | 3.34 | 1.12 |
| Slaughter Heifers 0-1 | May | 5.2 | 3.89 | 1.3 |
| Slaughter Heifers 0-1 | Jun | 5.06 | 3.79 | 1.27 |
| Slaughter Heifers 0-1 | Jul | 5.69 | 4.26 | 1.43 |
| Slaughter Heifers 0-1 | Aug | 5.95 | 4.46 | 1.49 |
| Slaughter Heifers 0-1 | Sep | 6.49 | 4.86 | *1.63‡* |
| Slaughter Heifers 0-1 | Oct | 6.49 | 4.86 | *1.63‡* |
| Slaughter Heifers 0-1 | Nov | 6.49 | 4.86 | *1.63‡* |
| Slaughter Heifers 0-1 | Dec | 6.49 | 4.86 | *1.63‡* |
| Slaughter Heifers 1-2 | Jan | 6.87 | 5.15 | 1.73 |
| Slaughter Heifers 1-2 | Feb | 6.6 | 4.95 | 1.66 |
| Slaughter Heifers 1-2 | Mar | 8.77 | 6.57 | 2.2 |
| Slaughter Heifers 1-2 | Apr | 8.66 | 6.48 | 2.17 |
| Slaughter Heifers 1-2 | May | 9.53 | 7.14 | 2.39 |
| Slaughter Heifers 1-2 | Jun | 8.81 | 6.6 | 2.21 |
| Slaughter Heifers 1-2 | Jul | 9.49 | 7.11 | 2.38 |
| Slaughter Heifers 1-2 | Aug | 9.51 | 7.12 | 2.39 |
| Slaughter Heifers 1-2 | Sep | 5.92 | 4.44 | 1.49 |
| Slaughter Heifers 1-2 | Oct | 5.58 | 4.18 | 1.4 |
| Slaughter Heifers 1-2 | Nov | 5.78 | 4.33 | 1.45 |
| Slaughter Heifers 1-2 | Dec | 6.34 | 4.75 | 1.59 |
| Slaughter Steers 0-1 | Jan | 2.31 | 1.73 | 0.58 |
| Slaughter Steers 0-1 | Feb | 2.54 | 1.9 | 0.64 |
| Slaughter Steers 0-1 | Mar | 5.32 | 3.98 | 1.34 |
| Slaughter Steers 0-1 | Apr | 5.63 | 4.21 | 1.41 |
| Slaughter Steers 0-1 | May | 6.58 | 4.93 | 1.65 |
| Slaughter Steers 0-1 | Jun | 6.4 | 4.79 | 1.61 |
| Slaughter Steers 0-1 | Jul | 7.15 | 5.36 | 1.8 |
| Slaughter Steers 0-1 | Aug | 7.48 | 5.6 | 1.88 |
| Slaughter Steers 0-1 | Sep | 8.32 | 6.23 | *2.09‡* |
| Slaughter Steers 0-1 | Oct | 8.32 | 6.23 | *2.09‡* |
| Slaughter Steers 0-1 | Nov | 8.32 | 6.23 | *2.09‡* |
| Slaughter Steers 0-1 | Dec | 8.32 | 6.23 | *2.09‡* |
| Slaughter Steers 1-2 | Jan | 8.86 | 6.64 | 2.23 |
| Slaughter Steers 1-2 | Feb | 8.58 | 6.43 | 2.15 |
| Slaughter Steers 1-2 | Mar | 11.45 | 8.57 | 2.87 |
| Slaughter Steers 1-2 | Apr | 11.19 | 8.38 | 2.81 |
| Slaughter Steers 1-2 | May | 12.29 | 9.2 | 3.09 |
| Slaughter Steers 1-2 | Jun | 11.26 | 8.44 | 2.83 |
| Slaughter Steers 1-2 | Jul | 12 | 8.99 | 3.01 |
| Slaughter Steers 1-2 | Aug | 11.99 | 8.98 | 3.01 |
| Slaughter Steers 1-2 | Sep | 7.45 | 5.58 | 1.87 |
| Slaughter Steers 1-2 | Oct | 7.03 | 5.26 | 1.76 |
| Slaughter Steers 1-2 | Nov | 7.35 | 5.5 | 1.84 |
| Slaughter Steers 1-2 | Dec | 8.08 | 6.05 | 2.03 |
| Slaughter Bulls 0-1 | Jan | 2.35 | 1.76 | 0.59 |
| Slaughter Bulls 0-1 | Feb | 2.55 | 1.91 | 0.64 |
| Slaughter Bulls 0-1 | Mar | 5.29 | 3.96 | 1.33 |
| Slaughter Bulls 0-1 | Apr | 5.59 | 4.18 | 1.4 |
| Slaughter Bulls 0-1 | May | 6.51 | 4.87 | 1.63 |
| Slaughter Bulls 0-1 | Jun | 6.34 | 4.75 | 1.59 |
| Slaughter Bulls 0-1 | Jul | 7.14 | 5.35 | 1.79 |
| Slaughter Bulls 0-1 | Aug | 7.47 | 5.6 | 1.88 |
| Slaughter Bulls 0-1 | Sep | 8.28 | 6.2 | *2.08‡* |
| Slaughter Bulls 0-1 | Oct | 8.28 | 6.2 | *2.08‡* |
| Slaughter Bulls 0-1 | Nov | 8.28 | 6.2 | *2.08‡* |
| Slaughter Bulls 0-1 | Dec | 8.28 | 6.2 | *2.08‡* |
| Slaughter Bulls 1-2 | Jan | 8.79 | 6.59 | 2.21 |
| Slaughter Bulls 1-2 | Feb | 8.52 | 6.38 | 2.14 |
| Slaughter Bulls 1-2 | Mar | 11.39 | 8.53 | 2.86 |
| Slaughter Bulls 1-2 | Apr | 11.23 | 8.41 | 2.82 |
| Slaughter Bulls 1-2 | May | 12.39 | 9.28 | 3.11 |
| Slaughter Bulls 1-2 | Jun | 11.46 | 8.58 | 2.88 |
| Slaughter Bulls 1-2 | Jul | 12.37 | 9.26 | 3.1 |
| Slaughter Bulls 1-2 | Aug | 12.41 | 9.29 | 3.12 |
| Slaughter Bulls 1-2 | Sep | 7.44 | 5.57 | 1.87 |
| Slaughter Bulls 1-2 | Oct | 7.03 | 5.27 | 1.77 |
| Slaughter Bulls 1-2 | Nov | 7.33 | 5.49 | 1.84 |
| Slaughter Bulls 1-2 | Dec | 8.07 | 6.05 | 2.03 |
| Breeding Growing Cows 2-3 | Jan | 7.09 | 5.31 | 1.78 |
| Breeding Growing Cows 2-3 | Feb | 6.66 | 4.99 | 1.67 |
| Breeding Growing Cows 2-3 | Mar | 8.72 | 6.53 | 2.19 |
| Breeding Growing Cows 2-3 | Apr | 8.68 | 6.5 | 2.18 |
| Breeding Growing Cows 2-3 | May | 9.66 | 7.24 | 2.43 |
| Breeding Growing Cows 2-3 | Jun | 9.39 | 7.03 | 2.36 |
| Breeding Growing Cows 2-3 | Jul | 10.52 | 7.88 | 2.64 |
| Breeding Growing Cows 2-3 | Aug | 11.88 | 8.9 | 2.98 |
| Breeding Growing Cows 2-3 | Sep | 6.8 | 5.09 | 1.71 |
| Breeding Growing Cows 2-3 | Oct | 6.23 | 4.66 | 1.56 |
| Breeding Growing Cows 2-3 | Nov | 6.15 | 4.61 | 1.54 |
| Breeding Growing Cows 2-3 | Dec | 6.57 | 4.92 | 1.65 |
| Breeding Mature Cows | Jan | 8.72 | 6.53 | 2.19 |
| Breeding Mature Cows | Feb | 8.27 | 6.2 | 2.08 |
| Breeding Mature Cows | Mar | 7.6 | 5.69 | 1.91 |
| Breeding Mature Cows | Apr | 7.39 | 5.54 | 1.86 |
| Breeding Mature Cows | May | 8.08 | 6.05 | 2.03 |
| Breeding Mature Cows | Jun | 7.78 | 5.83 | 1.95 |
| Breeding Mature Cows | Jul | 8.72 | 6.53 | 2.19 |
| Breeding Mature Cows | Aug | 9.95 | 7.45 | 2.5 |
| Breeding Mature Cows | Sep | 9.07 | 6.79 | 2.28 |
| Breeding Mature Cows | Oct | 8.14 | 6.09 | 2.04 |
| Breeding Mature Cows | Nov | 8 | 5.99 | 2.01 |
| Breeding Mature Cows | Dec | 8.3 | 6.21 | 2.08 |

‡ Applied an average of Jun-Aug and Jan-Mar.

## Sheep

**Table S3**. N excretion rates for different age classes of sheep (2021-2022), sourced from Pickering, Gibbs [3]. Note that estimated values are in italics with the method indicated in a footnote to the Table.

| Class | Month | Total Excreta kg N/head.month | Nitrogen Excreted In Urine kg N/head | Nitrogen Excreted In Faeces kg N/head |
| --- | --- | --- | --- | --- |
| Dry Ewes | Jan | 0.987 | 0.694 | *0.294*§ |
| Dry Ewes | Feb | 0.698 | 0.49 | *0.207*§ |
| Dry Ewes | Mar | 0.892 | 0.626 | *0.265§* |
| Dry Ewes | Apr | 0.857 | 0.602 | *0.255§* |
| Dry Ewes | May | 0.927 | 0.651 | *0.276§* |
| Dry Ewes | Jun | 0.893 | 0.627 | *0.266§* |
| Dry Ewes | Jul | 1.094 | 0.769 | *0.326§* |
| Dry Ewes | Aug | 1.392 | 0.978 | *0.414§* |
| Dry Ewes | Sep | 1.971 | 1.385 | *0.586§* |
| Dry Ewes | Oct | 1.417 | 0.995 | *0.422§* |
| Dry Ewes | Nov | 1.379 | 0.969 | *0.41§* |
| Dry Ewes | Dec | 1.44 | 1.012 | *0.429§* |
| Mature Breeding Ewes | Jan | 1.229 | 0.864 | 0.366 |
| Mature Breeding Ewes | Feb | 0.869 | 0.61 | 0.258 |
| Mature Breeding Ewes | Mar | 1.11 | 0.779 | 0.33 |
| Mature Breeding Ewes | Apr | 1.067 | 0.749 | 0.317 |
| Mature Breeding Ewes | May | 1.154 | 0.811 | 0.343 |
| Mature Breeding Ewes | Jun | 1.112 | 0.781 | 0.331 |
| Mature Breeding Ewes | Jul | 1.362 | 0.957 | 0.405 |
| Mature Breeding Ewes | Aug | 1.733 | 1.217 | 0.516 |
| Mature Breeding Ewes | Sep | 2.454 | 1.724 | 0.73 |
| Mature Breeding Ewes | Oct | 1.764 | 1.239 | 0.525 |
| Mature Breeding Ewes | Nov | 1.717 | 1.206 | 0.511 |
| Mature Breeding Ewes | Dec | 1.793 | 1.26 | 0.534 |
| Growing Breeding Sheep | Jan | 1.409 | 0.99 | 0.419 |
| Growing Breeding Sheep | Feb | 1.059 | 0.744 | 0.315 |
| Growing Breeding Sheep | Mar | 1.387 | 0.974 | 0.413 |
| Growing Breeding Sheep | Apr | 1.01 | 0.71 | 0.3 |
| Growing Breeding Sheep | May | 1.132 | 0.795 | 0.337 |
| Growing Breeding Sheep | Jun | 1.126 | 0.791 | 0.335 |
| Growing Breeding Sheep | Jul | 1.408 | 0.989 | 0.419 |
| Growing Breeding Sheep | Aug | 1.806 | 1.268 | 0.537 |
| Growing Breeding Sheep | Sep | 2.545 | 1.788 | 0.757 |
| Growing Breeding Sheep | Oct | 1.869 | 1.313 | 0.556 |
| Growing Breeding Sheep | Nov | 1.834 | 1.288 | 0.546 |
| Growing Breeding Sheep | Dec | 1.94 | 1.363 | 0.577 |
| Growing Non-Breeding Sheep | Jan | 1.229 | 0.863 | 0.366 |
| Growing Non-Breeding Sheep | Feb | 1.156 | 0.812 | 0.344 |
| Growing Non-Breeding Sheep | Mar | 1.516 | 1.065 | 0.451 |
| Growing Non-Breeding Sheep | Apr | 1.099 | 0.772 | 0.327 |
| Growing Non-Breeding Sheep | May | 1.21 | 0.85 | 0.36 |
| Growing Non-Breeding Sheep | Jun | 1.125 | 0.79 | 0.335 |
| Growing Non-Breeding Sheep | Jul | 1.221 | 0.858 | 0.363 |
| Growing Non-Breeding Sheep | Aug | 1.225 | 0.861 | 0.365 |
| Growing Non-Breeding Sheep | Sep | 1.195 | 0.839 | 0.356 |
| Growing Non-Breeding Sheep | Oct | 1.094 | 0.769 | 0.326 |
| Growing Non-Breeding Sheep | Nov | 1.099 | 0.772 | 0.327 |
| Growing Non-Breeding Sheep | Dec | 1.176 | 0.826 | 0.35 |
| Wethers | Jan | 0.951 | 0.668 | 0.283 |
| Wethers | Feb | 0.869 | 0.61 | 0.258 |
| Wethers | Mar | 1.11 | 0.779 | 0.33 |
| Wethers | Apr | 1.063 | 0.747 | 0.316 |
| Wethers | May | 1.133 | 0.796 | 0.337 |
| Wethers | Jun | 1.034 | 0.726 | 0.308 |
| Wethers | Jul | 1.094 | 0.769 | 0.326 |
| Wethers | Aug | 1.074 | 0.754 | 0.319 |
| Wethers | Sep | 1.024 | 0.72 | 0.305 |
| Wethers | Oct | 0.922 | 0.648 | 0.274 |
| Wethers | Nov | 0.894 | 0.628 | 0.266 |
| Wethers | Dec | 0.934 | 0.656 | 0.278 |
| Lambs-Sl1 | Jan | 1.195 | 0.84 | 0.356 |
| Lambs-Sl1 | Feb | 1.296 | 0.91 | 0.386 |
| Lambs-Sl1 | Mar | 1.028 | 0.722 | 0.306 |
| Lambs-Sl1 | Apr | 0.796 | 0.559 | 0.237 |
| Lambs-Sl1 | May | 0.903 | 0.634 | 0.269 |
| Lambs-Sl1 | Jun | 0.876 | 0.615 | 0.261 |
| Lambs-Sl1 | Jul | 0.966 | 0.679 | *0.287ɸ* |
| Lambs-Sl1 | Aug | 0.997 | 0.7 | *0.297ɸ* |
| Lambs-Sl1 | Sep | 0.047 | 0.033 | *0.014ɸ* |
| Lambs-Sl1 | Oct | 0.047 | 0.033 | 0.014 |
| Lambs-Sl1 | Nov | 0.277 | 0.195 | 0.083 |
| Lambs-Sl1 | Dec | 0.628 | 0.441 | 0.187 |
| Lambs-Sl2 | Jan | 1.195 | 0.84 | *0.356ɸ* |
| Lambs-Sl2 | Feb | 1.296 | 0.91 | *0.386ɸ* |
| Lambs-Sl2 | Mar | 1.028 | 0.722 | *0.306ɸ* |
| Lambs-Sl2 | Apr | 0.796 | 0.559 | *0.237ɸ* |
| Lambs-Sl2 | May | 0.903 | 0.634 | *0.269ɸ* |
| Lambs-Sl2 | Jun | 0.876 | 0.615 | *0.261ɸ* |
| Lambs-Sl2 | Jul | 0.966 | 0.679 | 0.287 |
| Lambs-Sl2 | Aug | 0.997 | 0.7 | 0.297 |
| Lambs-Sl2 | Sep | 0.047 | 0.033 | *0.014ɸ* |
| Lambs-Sl2 | Oct | 0.047 | 0.033 | *0.014ɸ* |
| Lambs-Sl2 | Nov | 0.277 | 0.195 | *0.083ɸ* |
| Lambs-Sl2 | Dec | 0.628 | 0.441 | *0.187ɸ* |
| Rams | Jan | 1.766 | 1.241 | 0.525 |
| Rams | Feb | 1.65 | 1.159 | 0.491 |
| Rams | Mar | 2.123 | 1.492 | 0.632 |
| Rams | Apr | 1.978 | 1.39 | 0.588 |
| Rams | May | 2.092 | 1.47 | 0.622 |
| Rams | Jun | 1.864 | 1.31 | 0.555 |
| Rams | Jul | 1.954 | 1.373 | 0.581 |
| Rams | Aug | 1.901 | 1.335 | 0.565 |
| Rams | Sep | 1.804 | 1.267 | 0.537 |
| Rams | Oct | 1.624 | 1.141 | 0.483 |
| Rams | Nov | 1.623 | 1.14 | 0.483 |
| Rams | Dec | 1.71 | 1.201 | 0.509 |

§ Applied MatureBreedingEwe_month * DryEwe_July / MatureBreedingEwe_July.

ɸ Applied values from the other lamb class for the same month.

## Deer

**Table S4**. N excretion rates for different age classes of deer (2021-2022), sourced from Pickering, Gibbs [3]. Note that estimated values are in italics with the method indicated in a footnote to the Table.

| Class | Month | Total Excreta kg N/head.month | Nitrogen Excreted In Urine kg N/head | Nitrogen Excreted In Faeces kg N/head |
| --- | --- | --- | --- | --- |
| Breeding Hinds 2+ yrs | Jan | 3.326 | 2.46 | 0.866 |
| Breeding Hinds 2+ | Feb | 2.953 | 2.184 | 0.769 |
| Breeding Hinds 2+ | Mar | 3.06 | 2.263 | 0.797 |
| Breeding Hinds 2+ | Apr | 2.223 | 1.644 | 0.579 |
| Breeding Hinds 2+ | May | 2.388 | 1.766 | 0.622 |
| Breeding Hinds 2+ | Jun | 2.543 | 1.881 | 0.662 |
| Breeding Hinds 2+ | Jul | 2.686 | 1.986 | 0.699 |
| Breeding Hinds 2+ | Aug | 2.973 | 2.199 | 0.774 |
| Breeding Hinds 2+ | Sep | 3.162 | 2.339 | 0.823 |
| Breeding Hinds 2+ | Oct | 2.859 | 2.115 | 0.745 |
| Breeding Hinds 2+ | Nov | 3.37 | 2.492 | 0.878 |
| Breeding Hinds 2+ | Dec | 3.302 | 2.442 | 0.86 |
| Hinds 0-1 | Jan | 0.112 | 0.083 | 0.029 |
| Hinds 0-1 | Feb | 0.232 | 0.171 | 0.06 |
| Hinds 0-1 | Mar | 0.723 | 0.535 | 0.188 |
| Hinds 0-1 | Apr | 1.201 | 0.888 | 0.313 |
| Hinds 0-1 | May | 0.974 | 0.72 | 0.254 |
| Hinds 0-1 | Jun | 1.489 | 1.101 | 0.388 |
| Hinds 0-1 | Jul | 1.7 | 1.257 | 0.443 |
| Hinds 0-1 | Aug | 1.824 | 1.349 | 0.475 |
| Hinds 0-1 | Sep | 1.884 | 1.394 | 0.491 |
| Hinds 0-1 | Oct | 1.792 | 1.325 | 0.467 |
| Hinds 0-1 | Nov | 1.813 | 1.341 | 0.472 |
| Hinds 0-1 | Dec | 1.92 | 1.42 | *0.5ɵ* |
| Hinds 1-2 | Jan | 2.099 | 1.552 | 0.547 |
| Hinds 1-2 | Feb | 1.967 | 1.455 | 0.512 |
| Hinds 1-2 | Mar | 2.521 | 1.864 | 0.656 |
| Hinds 1-2 | Apr | 2.562 | 1.895 | 0.667 |
| Hinds 1-2 | May | 2.835 | 2.097 | 0.738 |
| Hinds 1-2 | Jun | 2.932 | 2.168 | 0.763 |
| Hinds 1-2 | Jul | 3.174 | 2.347 | 0.826 |
| Hinds 1-2 | Aug | 3.489 | 2.581 | 0.909 |
| Hinds 1-2 | Sep | 3.71 | 2.744 | 0.966 |
| Hinds 1-2 | Oct | 3.417 | 2.527 | 0.89 |
| Hinds 1-2 | Nov | 4.016 | 2.97 | 1.046 |
| Hinds 1-2 | Dec | 1.931 | 1.428 | 0.503 |
| Stags 0-1 | Jan | 0.3 | 0.222 | 0.078 |
| Stags 0-1 | Feb | 0.412 | 0.305 | 0.107 |
| Stags 0-1 | Mar | 0.964 | 0.713 | 0.251 |
| Stags 0-1 | Apr | 1.456 | 1.077 | 0.379 |
| Stags 0-1 | May | 1.129 | 0.835 | 0.294 |
| Stags 0-1 | Jun | 1.777 | 1.314 | 0.463 |
| Stags 0-1 | Jul | 1.996 | 1.476 | 0.52 |
| Stags 0-1 | Aug | 2.137 | 1.58 | 0.556 |
| Stags 0-1 | Sep | 2.205 | 1.631 | 0.574 |
| Stags 0-1 | Oct | 2.1 | 1.553 | 0.547 |
| Stags 0-1 | Nov | 2.118 | 1.567 | 0.552 |
| Stags 0-1 | Dec | 0.107 | 0.079 | 0.028 |
| Stags 1-2 | Jan | 2.482 | 1.836 | 0.646 |
| Stags 1-2 | Feb | 2.329 | 1.723 | 0.607 |
| Stags 1-2 | Mar | 2.603 | 1.925 | 0.678 |
| Stags 1-2 | Apr | 2.637 | 1.95 | 0.687 |
| Stags 1-2 | May | 2.89 | 2.137 | 0.752 |
| Stags 1-2 | Jun | 2.755 | 2.038 | 0.717 |
| Stags 1-2 | Jul | 2.916 | 2.157 | 0.759 |
| Stags 1-2 | Aug | 2.946 | 2.179 | 0.767 |
| Stags 1-2 | Sep | 2.535 | 1.875 | 0.66 |
| Stags 1-2 | Oct | 2.29 | 1.693 | 0.596 |
| Stags 1-2 | Nov | 2.479 | 1.833 | 0.646 |
| Stags 1-2 | Dec | 2.256 | 1.669 | 0.588 |
| Stags 2-3 | Jan | 2.709 | 2.003 | 0.705 |
| Stags 2-3 | Feb | 2.461 | 1.82 | 0.641 |
| Stags 2-3 | Mar | 3.129 | 2.314 | 0.815 |
| Stags 2-3 | Apr | 3.15 | 2.33 | 0.82 |
| Stags 2-3 | May | 3.432 | 2.538 | 0.894 |
| Stags 2-3 | Jun | 3.258 | 2.409 | 0.848 |
| Stags 2-3 | Jul | 3.44 | 2.544 | 0.896 |
| Stags 2-3 | Aug | 3.459 | 2.559 | 0.901 |
| Stags 2-3 | Sep | 3.023 | 2.236 | 0.787 |
| Stags 2-3 | Oct | 2.724 | 2.015 | 0.709 |
| Stags 2-3 | Nov | 2.575 | 1.904 | 0.671 |
| Stags 2-3 | Dec | 2.586 | 1.912 | 0.673 |
| Breeding stags 3+ | Jan | 2.606 | 1.928 | 0.679 |
| Breeding stags 3+ | Feb | 2.368 | 1.751 | 0.617 |
| Breeding stags 3+ | Mar | 3.012 | 2.228 | 0.784 |
| Breeding stags 3+ | Apr | 3.032 | 2.242 | 0.789 |
| Breeding stags 3+ | May | 3.302 | 2.442 | 0.86 |
| Breeding stags 3+ | Jun | 3.133 | 2.317 | 0.816 |
| Breeding stags 3+ | Jul | 3.308 | 2.447 | 0.861 |
| Breeding stags 3+ | Aug | 3.326 | 2.46 | 0.866 |
| Breeding stags 3+ | Sep | 2.982 | 2.206 | 0.777 |
| Breeding stags 3+ | Oct | 2.694 | 1.993 | 0.702 |
| Breeding stags 3+ | Nov | 2.551 | 1.887 | 0.664 |
| Breeding stags 3+ | Dec | 2.488 | 1.84 | 0.648 |

ɵ Applied an average of Oct-Nov and Jan-Feb.

All other livestock equations calculate annual dung N excretion. This is to be distributed evenly, and proportional to the months these livestock types are present.

## Outdoor pigs

Total N_ex_ = head x N_ex_ factor

where:

Total N_ex_ Excreted nitrogen in kilograms per year – listed as 11.05 kg head^-1^ yr^-1^ [4]

Head Number of animals – **a user-inputted figure**

N_ex_ factor Nitrogen excretion rate (N/head/year)

Note that NZPork have advised the MPI inventory equation for swine is not appropriate as the IPCC calculations are for an “average” pig. Outdoor sows deposit more N than an average pig. The replacement calculation could be updated once provided.

## Poultry

These equations refer to the combined annual excretion of N in urine and dung. We split N inputs equally between urine and dung when applying an index.

Total N_ex_ = head x N_ex_ factor

where:

Total N_ex_ Excreted nitrogen in kilograms per year

Head Number of animals – **a user-inputted figure**

N_ex_ factor Nitrogen excretion rate (N/head/year)

- Broilers Total Nex = head x 0.39
- Layers Total Nex = head x 0.42
- Other (including ducks, turkeys, emus, ostriches) Total Nex = head x 0.60

## Goats

These equations refer to the combined annual excretion of N in urine and dung. We split N inputs equally between urine and dung when applying an index. We suspect that meat goats and dairy goats may excrete different amounts of N in urine, but have no data on this so treat them as equal.

Total N_ex_ = head x N_ex_ factor

where:

Total N_ex_ Excreted nitrogen in kilograms per year

Head Number of animals – **a user-inputted figure**

N_ex_ factor Nitrogen excretion rate (N/head/year)

| Goat type | Equation |
| --- | --- |
| Dairy | Total Nex = head x 12.7 |
| Non-dairy | Total Nex = head x 10.6 |

## Horses, mules and asses

These equations refer to the combined annual excretion of N in urine and dung. We split N inputs equally between urine and dung when applying an index.

Total N_ex_ = head x N_ex_ factor

where:

Total N_ex_ Excreted nitrogen in kilograms per year

Head Number of animals – **a user-inputted figure**

N_ex_ factor Nitrogen excretion rate (25 N/head/year)

## Alpacas

Use values for mature breeding ewes.

# Sources of N from residues

The total mineral N inputs (the amount of N/ha to be multiplied by the transport factor) is calculated by summing total mineral N inputs from fertiliser, composts, and residues for the month.

Users need to input a crop type (including fallow) for every month from a drop-down box and a yield for the month of harvest.

To estimate N losses from **runoff**, only use estimates for above-ground residues (i.e., Eqn 1).

To estimate N losses from **leaching** use above- and below-ground residues (after removing N that is immobilised) and any modifications from sections 2 to 5 (i.e., Eqn 2 plus Eqn 3 onwards).

Mineral N inputs from residues, and monthly crop uptake, are calculated as described below.

**1. Estimating average soil mineral N inputs from crop residues**

The amount of nitrogen (N) in crop residues is based on the work of Pickering, Gibbs [3], who calculated N content for the purposes of calculating nitrous oxide emissions. The fact that some residues immobilise N and some release N is crudely accounted for by assuming mineralisation in residues with a C:N ratio below a critical value of 25 [5], and immobilisation (mineral N taken up from the soil) by residues with a high C:N ratio. This agrees with the critical C:N ratio of 24 found by Trinsoutrot, Recous [6], but a lower critical C:N ratio of 18 [7] or higher critical C:N ratios of 37 [8], 40 [9], and 44 [10] have been found in other studies. This indicates that the C:N ratio alone is not particularly accurate, and better relationships have been found by including lignin content into decomposition equations [8, 10], which include the rate of N release. However, this information is not available for all crops, so the C:N ratio has been used in this instance. It is assumed that N supply from burnt residues is small, so is not accounted for in this version, but can be included in later versions if needed. There will be no immobilisation if residues are burnt.

The amount of N supplied by crop residues (if the C:N ratio is <25) is calculated according to equation 1 below

Eqn 1. 𝐴𝐺𝑁*R*,*c* = 𝐷𝑀𝐹𝑐 × (𝑃𝑟𝑜𝑑𝑐 / 𝐻𝐼𝑐 − 𝑃𝑟𝑜𝑑𝑐) × 𝑁𝐴𝐺

Where:

- AGNR,c = Amount of above-ground nitrogen returned to soils through incorporation of crop residues for crop type c (kg N ha^-1^)
- DMFc = Dry matter factor, used to convert the tonnes of fresh residues produced to tonnes of dry matter produced for crop type c (see Table S5 for values)
- Prodc = Annual production of crop type, c (kg ha^-1^). This is entered by the grower
- HIc = Harvest index, fraction of the crop (c) that is harvested for the primary purpose of growing the crop (see Table S5 for values)
- NAG,c = Nitrogen content of above-ground residue for crop type c (kg N kg^-1^ Dry Matter) (see Table S5 for values)

Above-ground N is assumed to be available to runoff, whereas both above- and below-ground N from residues are assumed to be available to leaching.

The amount of N supplied by roots from crop residues is calculated according to equation 2 below.

Eqn 2. 𝐵𝐺𝑁,*c* = 𝐷𝑀𝐹𝑐 × ( 𝑃𝑟𝑜𝑑𝑐 / 𝐻𝐼𝑐 ) × 𝑅𝑆𝑐 × 𝑁𝐵𝐺,c

Where:

- BGN,c = Amount of below-ground nitrogen returned to soils after the crop through incorporation of crop residues for crop type c (kgN ha^-1^)
- DMFc = Dry matter factor, used to convert total production to dry matter crop production for crop type c (see Table S5 for values)
- Prodc = Annual production of crop type, c (kg/ha). This is entered by the grower.
- HIc = Harvest index, fraction of the crop (c) that is harvested for the primary purpose of growing the crop (see Table S5 for values)
- RSc = Root:shoot ratio for crop type c. assumed to be 0.1 for all crops [11]
- NBG,c = Nitrogen content of below-ground residue for crop type c (kg N/kg DM) (see Table 1 for values)

The amount of mineral N (in kg N/ha) supplied by mineralisation of above-ground (NminAGR) and below-ground (NminBGR) crop residues (if the C:N ratio is <25) is calculated by the formulae below (Equations 3 and 4). If C:N is between 25 and 40, it is assumed that no N is released from residues. The nitrogen content of the residues is multiplied by 0.6, since approximately 60% of the nitrogen in the residues is released by mineralisation in the short term [10]. This is a very crude approximation which in future versions could be made to vary with residue type. The remaining 40% of the N becomes part of the organic N pool, which is slowly released over many years, and not considered in this N leaching risk index tool.

Eqn 3. If C:N_AG_<25, NminAGR = 𝐴𝐺𝑁*R*_c_ x 0.6

Where C:N_AG_ = the carbon to nitrogen ratio of the aboveground biomass (see Table S5 for values)

Eqn 4. NminBGR = *B*𝐺𝑁*R*_c_ x 0.6

Data for C:N ratios for roots of each crop is difficult to find. For crops where data were available, the values were often between 30 and 40 [12], and the amounts of mineral N either immobilised or released was variable. However, root N only comprises a small amount of the N contribution of crop residues, so N release has been assumed.

The total amount (kg N ha^-1^) of N mineralised (NminT) is the sum of that supplied from mineralisation the above- and below-ground residues

(NminT) = NminAGR + NminBGR

For forage cereals, stock numbers are input and NminBGR are calculated but NminAGR is assumed to be zero due to grazing. For crops that are grazed, values yield values as for any crop and stock numbers in the appropriate months. Stock numbers should be a maximum number in the month i.e., not averaged for part of a month or across the farm.

The monthly release of this amount of nitrogen will be apportioned as described in Table S5.

**Table S5**. Proportion of crop residue N released per month after incorporation into the soil.

| 1^st^ month | 2^nd^ month | 3^rd^ month |
| --- | --- | --- |
| 70% | 20% | 10% |

This is a very crude approximation of the release rates provided by De Neve and Hofman [10]. Greater accuracy may be achieved in future iterations of the index by providing different release rates for different seasons. Rates of N release or immobilisation will be slower if the residues remain on the soil surface, since N release can only occur if there is adequate soil moisture, and immobilisation of soil N only occurs when the residues are in contact with soil N [35].

Immobilisation by residues with C:N>40 may be crudely estimated according to the relationship from Trinsoutrot, Recous [6].

N immobilised = 14.6 x N_residues_ – 24.6

Were N immobilised is g N kg^-1^ residual C and N_residues_ is organic N in residues (g kg^-1^ Dry Matter)

Rearranging this becomes:

N immobilised (kg ha^-1^) = kg residual C ha^-1^ x (14.6 x NAG x 1000 – 24.6)/1000

and kg residual C ha^-1^ = 𝐴𝐺𝑁*R*,*c* x C:N ratio.

This immobilisation could be assumed to occur within a month of incorporation after harvest, since laboratory studies often show that immobilisation by crop residues is often rapid [6], although again there is much variability depending on factors such as residue type, temperature and degree of incorporation into the soil. If sufficient immobilisation occurs, the risk for the month of immobilisation can be zero.

Nitrogen inputs from perennial crop prunings are not included in Table S6 since they are not likely to contribute to increased risk of N leaching in the short term. They are considered to enter the slow-release organic N pool, which is not considered in this index, except to highlight N risk during periods of fallow and crop establishment [36].

**Table S6**. Parameters for the calculation of crop residue N content and crop N uptake. These are rooting depth, harvest index (HI), the nitrogen concentration in the above-ground residues (NAGR) and below-ground residues (NBG), the dry matter factor (DM), and the carbon to nitrogen ratio (C:N). Most of the data for HI, NAG, NBG and DMF are from Pickering et al. 2022. Rooting depths from Lott and Hammond [13] and Alberta Agriculture and Forestry [14] and apply only to leaching. Other data as listed in the References column.

| Species | Rooting depth | HI | NAG  (kg N/kg DM) | NBG  (kg N/kg DM) | DMF residues | C:N | References |
| --- | --- | --- | --- | --- | --- | --- | --- |
| Wheat | Deep | 0.41 | 0.005 | 0.009 | 0.86 | 58 | [15] |
| Barley | Deep | 0.46 | 0.005 | 0.009 | 0.86 | 58 | [16] |
| Oats | Deep | 0.30 | 0.005 | 0.009 | 0.86 | 58 | [17] |
| Forage cereal | Deep | 0.37 | Na | 0.009 | 0.13 | 20 | [18] |
| Maize (grain) | Deep | 0.50 | 0.007 | 0.007 | 0.86 | 79 | [19] |
| Field seed peas | Intermediate | 0.50 | 0.020 | 0.015 | 0.86 | 28 | [20] |
| Peas fresh and processed | Intermediate | 0.45 | 0.030 | 0.015 | 0.21 | 12* |  |
| Potatoes | Intermediate | 0.90 | 0.020 | 0.010 | 0.22 | 22 | [21] |
| Onions | Shallow | 0.80 | 0.020 | 0.010 | 0.11 | 23 | [22] |
| Sweet corn | Intermediate | 0.55 | 0.009 | 0.007 | 0.24 | 32 | [23] |
| Squash | Intermediate | 0.80 | 0.020 | 0.010 | 0.20 | 12* |  |
| Herbage seeds | Intermediate | 0.11 | 0.015 | 0.010 | 0.85 | 30* |  |
| Legume seeds | Intermediate | 0.09 | 0.040 | 0.010 | 0.85 | 30* |  |
| Brassica seeds | Intermediate | 0.20 | 0.010 | 0.008 | 0.85 | 30* |  |
| Cauliflower | Intermediate | 0.24 | 0.023 | 0.010 | 0.12 | 17 | [24, 25] |
| Broccoli | Intermediate | 0.35 | 0.015 | 0.010 | 0.20 | 26 | [16, 26] |
| Beans | Intermediate | 0.37 | 0.033 | 0.010 | 0.17 | 11 | [27, 28] |
| Carrots | Intermediate | 0.77 | 0.022 | 0.010 | 0.18 | 24 | [28] |
| Beetroot | Intermediate | 0.85 | 0.030 | 0.010 | 0.28 | 10 | [28] |
| Tomatoes | Intermediate | 0.67 | 0.022 | 0.010 | 0.19 | 10 | [28] |
| Lettuce | Shallow | 0.38 | 0.024 | 0.041 | 0.07 | 12 | [15, 29, 30] |
| Cabbage | Intermediate | 0.70 | 0.029 | 0.013 | 0.15 | 14 | [31, 32] |
| Brussels sprouts | Intermediate | 0.35 | 0.021 | 0.009 | 0.18 | 15 | [9,[33] |
| Celery | Shallow | 0.50 | 0.024 | 0.020 | 0.17 | 15 | [10, 29, 34] |
| Grey pumpkin | Intermediate | 0.86 | 0.014 | 0.010* | 0.12 | 30* | [21] |
| Asian greens (e.g., Pak Choi) | Shallow | 0.60* | 0.024* | 0.030* | 0.08* | 12* |  |
| Leeks | Shallow | 0.70* | 0.029 | 0.036 | 0.11 | 12 | [8, 15] |
| Spinach | Shallow | 0.70* | 0.025* | 0.010* | 0.12* | 12* |  |
| Long term pasture - dairy (prior to cultivation) | | See section 3 below | | | | | |
| Long term pasture - sheep, beef, deer (prior to cultivation) | | See section 3 below | | | | | |
| Short term pastures (prior to cultivation) | | See section 3 below | | | | | |
| Green manure |  | See section 4 below | | | | | |
| Fallow |  | See section 5 below | | | | | |

*Estimated values

**2. Effect of rooting depth on the risk of nitrate leaching**

Nitrogen applied to deep-rooting crops that have a high N requirement typically have a lower risk of leaching than N applied to shallow-rooting crops. To account for this, the sum of monthly nitrogen leaching risk for the different crops in Table S6 is multiplied by a rooting depth factor (Table S7). The nitrogen leaching risk for deep-rooting crops will be multiplied by 0.7 (i.e., N leaching is reduced relative to pasture at 60 cm rooting depth), and for shallow-rooting crops the N leaching risk will be multiplied by 1.4 (i.e., risk is increased relative to pasture). For crops with intermediate-rooting depths, the multiplier is 1, i.e., the risk is like to pasture. This is summarised in the table below. For fallow the multiplier is 1.8.

**Table S7**. Multiplier to apply to the sum of monthly N loss during the months where each crop is grown.

| Rooting depth | N leaching risk multiplier |
| --- | --- |
| Shallow | 1.4 |
| Intermediate | 1.0 |
| Deep | 0.7 |

**3. Estimating average soil mineral N inputs to leaching from cultivation of long- and short-term pasture residues**

Soil mineral N inputs from pasture residues are calculated based on the methodology of Thomas, Wallace [36]. Users indicate pasture as the crop type for all months from January preceding a new crop or fallow.

The N supplied from long-term dairy, sheep, beef or deer pastures, whereby pasture residues N_p_ (kg ha^-1^) is calculated as:

Eqn 5. 𝑁,*p* = (AG_DM_ × N_AG_) + (BG_DM_ × 𝑁_𝐵𝐺_)

Where:

- AG_DM_ is the above-ground dry matter, which in dairy pastures, is taken to be 1.4 Mg DM/ha, and in sheep and beef pastures is 0.75 Mg DM/ha.
- N_AG_ is assumed to be 2% for both sheep and beef and dairy pastures.
- BG_DM_ for sheep and beef pastures is taken to be 7.2 Mg DM/ha, and 2.8 Mg DM/ha for dairy pastures.
- 𝑁_𝐵𝐺_ is taken to be 1.2% for sheep and beef pastures and 1.6% for dairy pastures.

For short-term pastures (i.e., those that are only present in the system for < 2 years), AG_DM_ is 1.2 Mg DM/ha, BG_DM_ is 2 Mg DM/ha N_BG_ is 1.4% and N_AG_ is 2%.

This N is assumed to be converted to mineral N distributed according to the pattern shown in Table S8. Note that there is much variation in the rate of conversion of these pastoral N residues into mineral N [8, 27], as this depends on numerous factors such as soil temperature, degree of incorporation, soil moisture, and pasture composition, which would be much more accurately described by a model.

**Table S8**. Percentage of pasture residue N converted to mineral N per month following incorporation into soil. Twenty percent is assumed to slowly mineralise after four months, but at a sufficiently slow rate that it can be ignored. The remaining 20% is assumed to go into the long-term N pool that is not at risk of leaching in the short-term.

| 1^st^ month | 2^nd^ month | 3^rd^ month |
| --- | --- | --- |
| 40 | 15 | 5 |

**4. Green manure crops**

Green manures crops are defined as short-term crops (commonly three months or less) that are planted for the purpose of supplying nutrients (e.g., N) to the subsequent crop, so they typically have a high leaf N concentration. Green manure crops are distinct from catch crops, which have an extensive, deep root system, a longer growing period and a lower leaf N concentration.

The amount of mineral N from green manures (GMN) available for leaching is estimated according to equation 6.

Eqn 6. GMN = 𝑃𝑟𝑜𝑑𝑐 × 𝑁𝐴𝐺 X 0.8

The nitrogen supplied by green manures is multiplied by 0.8, since approximately 70% of the crop N is released by mineralisation, with an additional 10% N added to account for mineral N supply from the roots. The remaining 20% of the crop N is assumed to go into the slow-release organic N pool. The green manure N would be distributed according to the pattern in Table S6. For simplicity, only two categories of green manures are considered,

1) grass and cereal green manures, with a NAG concentration of 0.012 kg N kg^-1^ DM

2) all other green manures, with a NAG concentration of 0.03 kg N kg^-1^ DM [37].

Note that in this instance, *Prodc* has the units of kg DM ha^-1^, since it is assumed that growers will be more familiar with this unit for green manure crop yield, than with kg FW ha^-1^.

**5. Process for handling fallow periods and crop establishment**

The risk of leaching N losses is greatly increased during fallow periods, when N uptake and crop cover is non-existent or very small. To account for this, soil N mineralisation is added in. This rate varies with factors such as temperature and paddock management history. We have crudely estimated the supply of mineral N from mineralisation (Nmin) as 0.5 kg N ha^-1^ day^-1^ in the summer, and 0.25 kg N ha^-1^ day^-1^ in the winter, with intermediate values for September and April (Table S9). Soil N mineralisation will be included as a N input for the months of fallow plus the first month after sowing a crop.

**Table S9.** Table of soil N mineralisation values (Nmin, kg N ha^-1^ month^-1^) to be added as a N input during fallow periods, starting with the first month after harvest of a crop and up to (i.e., including) the first month of the subsequent crop.

| Month | J | F | M | A | M | J | J | A | S | O | N | D |
| --- | --- | --- | --- | --- | --- | --- | --- | --- | --- | --- | --- | --- |
| Nmin | 15.5 | 14 | 15.5 | 10 | 7.75 | 7.5 | 7.75 | 7.75 | 10 | 15.5 | 15 | 15.5 |

# Sources of N from fertiliser

The data in Table S10 and S11 are offered to the user if they do not know the N concentration of their product. Additional fertilisers from other suppliers can be added as needed, provided they supply an analysis of N concentration and that the concentration for the product is consistent with time.

Nitrogen application rates are calculated as the kg of product by the %N/100.

**Table S10**. Representative N fertiliser concentrations from Ballance Agri-Nutrients and Ravensdown Fertiliser Co-operative, effective 19 and 16^th^ of August, respectively [38, 39].

| Ballance fertiliser | % N | Ravensdown Fertiliser | % N |
| --- | --- | --- | --- |
| SustaiN | 45.9 | N-Protect | 45.9 |
| SustaiN 15K | 32.1 | Urea | 46 |
| SustaiN 20K | 27.5 | Granular Ammonium Sulphate | 20 |
| SustaiN 25K | 23.0 | Nitrogen Super | 6 |
| SustaiN Ammo 30N | 29.8 | Calcium Ammonium Nitrate (CAN) | 27 |
| SustaiN Ammo 36N | 35.4 | Ammo 31 | 30.4 |
| PhaSedN | 25.3 | Ammo 36 | 35.6 |
| PhaSedN Quick Start | 31.3 | Nitro S ™ | 29.9 |
| PastureSure 5K | 9.5 | N-Protect S ™ | 29.8 |
| PastureSure 10K | 7.6 | Ureammopot | 25.7 |
| PastureSure 15K | 7.6 | Flexi-N (South Island only) | 43.2 |
| PastureSure 15S | 9.5 | Flexi-N (North Island only) | 45.3 |
| PastureSure Boost | 9.1 | Flowfert N (South Island only) | 18 |
| PastureSure Balancer | 6.0 | Super Mag N | 6.9 |
| PastureSure Impact | 12.1 | 15% Granular Potash Super Mag N | 5.9 |
| PasturemagPlus (with SustaiN) | 6.9 | 20% Granular Potash Super Mag N | 5.5 |
| PasturemagPlus 5K (with SustaiN) | 6.2 | Dairy Pasture Boost 4 | 4 |
| PasturemagPlus 10K (with SustaiN) | 5.5 | Dairy Pasture Boost 6 | 4 |
| PasturemagPlus 15K (with SustaiN) | 4.8 | Dairy Pasture Boost 10 | 4 |
| PasturemagPlus 12N (with SustaiN) | 11.5 | Dairy Pasture Boost 12 | 4 |
| PasturemagPlus Hay & Silage (with SustaiN) | 9.2 | Pasture 6 Ravensdown Bulk | 5.5 |
| Nrich Urea | 46.0 | Cropmaster® DAP | 17.6 |
| Nrich SOA | 19.5 | DAP 13 S | 10.6 |
| Nrich Ammo 30N | 29.8 | Cropmaster® 11 | 10.6 |
| Nrich Ammo 36N | 35.4 | Cropmaster® 13 | 12.3 |
| Cropzeal 15P | 13.2 | Cropmaster® 15 | 14.8 |
| Cropzeal 16N | 15.2 | Cropmaster® 16 High K Bulk | 15.4 |
| Cropzeal 20N | 19.1 | Cropmaster® 20 | 18.8 |
| Cropzeal Boron Boost | 16.0 | Cropmaster® Brassica mix | 14.1 |
| DAP | 17.6 | Cropmaster® Brassica + Boron Blend | 13.6 |
| DAP Sulphur Super | 10.6 | Ammo-Phos® MAP | 10 |
| 20% Potash DAP Sulphur Super | 8.5 | Ammo-Phos® / Hycrop 7-15-15 | 7 |
| YaraMila Actyva S 15-7-12.5 | 15.0 | Ammo-Phos® / Hycrop 9-19-7 | 8.5 |
| YaraMila 12-10-10 | 13.0 | Nitrophoska® Select | 15 |
| YaraMila 8-11-20 | 8.0 | Nitrophosak Extra (North Island only) | 12 |
| YaraMila Complex | 12.0 | Cropstart 12-5-14 | 12 |
| YaraMila GrowerNZ | 13.0 | Compound Extra | 12 |
| YaraBela CAN | 27 | Cropstart Select | 15 |
| YaraLiva Nitrabor | 15.4 | Potash Gold 7-15-13 | 7 |
| YaraRega 9-0-30 | 9.0 | Potash Gold 15-10-10 | 14.2 |
| Pure Protamin | 13 | Potash Gold 14-7-14 | 14.3 |
|  |  | Urea | 46 |
|  |  | Granular Ammonium Sulphate | 20 |
|  |  | Calcium Ammonium Nitrate (CAN) | 27 |
|  |  | Cropmaster® DAP | 17.6 |
|  |  | Cropmaster® 15 | 14.8 |
|  |  | Cropmaster® 20 | 18.8 |
|  |  | Garden Fertiliser | 6.6 |
|  |  | Lawn Fertiliser | 14.5 |
|  |  | Avocado Regular Mix + TE | 9.6 |
|  |  | Cropstart Select | 15 |

**Table S11**. Dry matter and N content of dairy slurry, manures and poultry manures to be considered as fertiliser N inputs. Note that these are separate from farm dairy effluent applied up to nine months of the year. Data from [40-44].

| Manure type | Dry matter content (%) | N content (%) |
| --- | --- | --- |
| Scraped solids | 25.9 | 5.9 |
| Bunker manure | 23.1 | 5.6 |
| Manure + residues scraped from carbon-rich pads | 38.2 | 3.7 |
| Solids behind a weeping wall | 22.5 | 2.4 |
| Mechanically separated solids | 25.9 | 5.9 |
| Farm dairy effluent slurry from a stirred pond | 1.7 | 0.6 |
| Poultry manure | 66 | 1.9 |
| Poultry compost | 56 | 2.5 |

# Source of N from erosion

We estimated nitrogen (N) losses from soil erosion from erosion estimates of sediment and soil N concentrations for different soil orders, land uses and annual rainfall amounts.

**Estimating soil erosion losses**

Observations for sediment loss were obtained from the literature (Table S12). These were used to generate mean observed sediment losses for land use and slope classes (flat, rolling, easy and steep corresponding to <7, 7-15, 15.01-25, and >25 degrees, respectively) where there were three or more studies (Table S12). Too few data were available to make any further inferences on the role of different practices or land use intensity within each of these land use by slope classes. The only exception was, grazed winter forage cropping (as a land use management) which has been well studied owing to its higher sediment loss compared to pasture grazed in winter [45].

**Table S12**. Sediment yields and mean annual rainfall observed for farm to catchment scale studies of different land uses (and grazed winter forage crops) and slope classes across New Zealand.

| **Land Use and Management** | **Sediment yield (kg ha^-1^ yr^-1^)** | | **Mean annual rainfall (mm)** | **Slope class (degrees)** | **References** |
| --- | --- | --- | --- | --- | --- |
| Arable | 130 | 1100 | | 11.5 | [46] |
| Arable | 230 | 800 | | 3.5 | [47]^1^ |
| Dairy | 1250 | 780 | | 11.5 | [48] |
| Dairy | 142 | 1132 | | 3.5 | [49] |
| Dairy | 58 | 850 | | 3.5 | [50] |
| Dairy | 67 | 1132 | | 3.5 | [51] |
| Dairy | 38 | 1160 | | 3.5 | [52, 53] |
| Dairy | 149 | 1250 | | 3.5 | [52, 53] |
| Dairy | 72 | 1330 | | 3.5 | [52, 53] |
| Dairy | 883 | 4830 | | 3.5 | [52, 53] |
| Dairy | 32 | 900 | | 3.5 | [52, 53] |
| Deer (drystock) | 4480 | 687 | | 19 | [54] |
| Deer (drystock) | 3950 | 944 | | 11.5 | [54] |
| Deer (drystock) | 3356 | 687 | | 19 | [55] |
| Deer (drystock) | 158 | 1100 | | 3.5 | [56] |
| Deer (drystock) | 850 | 1300 | | 19 | [57] |
| Deer (drystock) | 2068 | 800 | | 11.5 | [57] |
| Deer (drystock) | 398 | 800 | | 19 | [57] |
| Exotic forest | 140 | 1300 | | 35 | [58] |
| Exotic forest | 40 | 1550 | | 19 | [59] |
| Native forest | 320 | 1600 | | 35 | [60] |
| Native forest | 320 | 1600 | | 35 | [60] |
| Native forest | 27 | 1500 | | 19 | [61] |
| Native forest | 600 | 1664 | | 35 | [62] |
| Native forest | 240 | 2600 | | 35 | [63] |
| Native forest | 270 | 1550 | | 19 | [59] |
| Sheep and Beef (drystock) | 700 | 1200 | | 19 | [64] |
| Sheep and Beef (drystock) | 1220 | 1200 | | 19 | [65] |
| Sheep and Beef (drystock) | 97 | 690 | | 19 | [66] |
| Sheep and Beef (drystock) | 374 | 1401 | | 19 | [67] |
| Sheep and Beef (drystock) | 1400 | 1000 | | 35 | [68] |
| Sheep and Beef (drystock) | 22 | 1500 | | 19 | [61] |
| Sheep and Beef (drystock) | 2632 | 1600 | | 35 | [60] |
| Sheep and Beef (drystock) | 128 | 1923 | | 11.5 | [69] |
| Sheep and Beef (drystock) | 2740 | 1200 | | 19 | [65] |
| Sheep and Beef (drystock) | 183 | 1006 | | 11.5 | [70] |
| Sheep and Beef (drystock) | 970 | 1664 | | 35 | [62] |
| Sheep and Beef (drystock) | 430 | 1300 | | 19 | [58] |
| Sheep and Beef (drystock) | 220 | 1550 | | 19 | [59] |
| Horticulture (Vegetables)^2^ | 7000 | 1200 | | 19 | [71] |
| Horticulture (Vegetables)^2^ | 16000 | 1200 | | 19 | [72] |
| Horticulture (Vegetables) | 490 | 1200 | | 3.5 | [73] |
| Winter forage crop (grazed) | 1012 | 800 | | 11.5 | [74] |
| Winter forage crop (grazed) | 1980 | 700 | | 11.5 | [75] |
| Winter forage crop (grazed) | 1100 | 1100 | | 11.5 | [76] |
| Winter forage crop (grazed) | 204 | 1100 | | 11.5 | [76] |
| Winter forage crop (grazed) | 640 | 1083 | | 11.5 | [45] |
| Winter forage crop (grazed) | 400 | 1083 | | 11.5 | [45] |

^1^ Data taken for catchments in the UK dominated by arable cropping (>70%) where the rainfall (600-900 mm), soil texture (silt loam), and slope (flat) were considered similar to those likely in New Zealand.

^2^ Data not included as vegetable growing on easy slopes is no longer likely under the National Policy Statement for Freshwater Management which effectively bans or discourages intensive land use on slopes >15^o^ [77].

**Table S13**. Mean observed annual sediment yields (kg ha^-1^) from different land uses at each slope.

| **Land use and management** | **Flat** | **Rolling** | **Easy** | **Steep** |
| --- | --- | --- | --- | --- |
| Arable | 180 | 180 |  |  |
| Dairy | 180 | 1250 |  |  |
| Deer (drystock) | 158 | 2517^1^ | 2517^1^ |  |
| Exotic forest |  |  | 167 | 167 |
| Native forest |  |  | 296 | 296 |
| Sheep and Beef (drystock) |  | 156 | 725 | 1667 |
| Horticultural (Vegetables) | 490 |  |  |  |
| Winter forage crop (grazed) |  | 889 |  |  |

^1^These observations were excluded from the analysis owing to the bias caused the large influence of wallows on the data compared to the presence of wallowing in a normal deer farm.

Estimates for seasonal cover factors used in the Revised Universal Soil Loss Equation (RUSLE) were taken from Donovan [78] for New Zealand. As no New Zealand data were available for vegetables, these were sourced from a study of European soils [79] and the data for temperate soils from a study of global soils [80] (Table S14). These data were then adjusted by multipliers (from 70-800) to yield values that were like annual sediment yields (Table S15; Figure S1). We have isolated the cover factor as the dominant human-influenced factor within RUSLE. No data were readily available for practice values, but through prior calibration in New Zealand, land management practices are bundled within cover factors, e.g., see [81, 82]. However, we have produced these estimates to make use of user-supplied data on land use and slope (also used for the filtering of mitigations and modifiers).

**Table S14**. Seasonal cover factors and soil total N concentration by land use and management.

| **Land use and Management** | **Total N (g kg^-1^)** | **Spring** | **Summer** | **Autumn** | **Winter** |
| --- | --- | --- | --- | --- | --- |
| Native forest | 3.20 | 0.002 | 0.0012 | 0.0012 | 0.003 |
| Exotic forest | 3.20 | 0.005 | 0.004 | 0.004 | 0.007 |
| Dairy | 6.18 | 0.04 | 0.03 | 0.03 | 0.05 |
| Drystock | 5.00 | 0.04 | 0.03 | 0.03 | 0.05 |
| Arable (incl. perennial horticulture) | 3.50 | 0.3 | 0.28 | 0.33 | 0.35 |
| Horticultural (Vegetables) | 8.40 | 0.35 | 0.28 | 0.43 | 0.43 |
| Winter forage crop (grazed) | 5.00 | 0.05 | 0.04 | 0.04 | 0.06 |

**Table S15**. Values of sediment loss derived using New Zealand cover factors, adjusted for different slope classes. Values for adjustment are in parentheses and are derived using expert opinion^1^. Values in bold have corresponding observations.

| **Land use and management** | **Flat** | **Rolling** | **Easy** | **Steep** |
| --- | --- | --- | --- | --- |
| Native forest | 21 (800) | 53 (800) | **85 (800)** | **328 (1600)** |
| Exotic forest | 28 (400) | 75 (400) | 120 (400) | 226 (400) |
| Dairy | **158 (300)** | **822 (600)** | 1317 (600) | 2481 (600) |
| Drystock | **158 (300)** | **411 (300**) | **878 (400)** | **1654 (400)** |
| Arable (incl. perennial horticulture) | **441 (100)** | **841 (70)** | 1358 (70) | 2505 (70) |
| Horticultural (Vegetables) | **522 (100)** | **1409 (100)** | 2317 (100) | 4255 (100) |
| Winter forage crop (grazed) | **399 (600)** | **1050 (600)** | 1680 (600) | 3150 (600) |

^1^ We adjusted cover factors based on empirical evidence that erosion rates increase with slope. However, we did not adjust cover factors where empirical evidence did not exist or because the land use was unlikely such as for arable, horticultural, dairy and winter forage crops on easy and steep slopes.

**Figure S1**. Plot of observed versus expected annual losses of sediment.

Estimated sediment losses by land use (and management) and slope class were then multiplied by total soil N concentrations sourced from sampling conducted by Regional Authorities from 1995 to 2017 and reported to the Ministry for the Environment and Statistics New Zealand as part of State of the Environment reporting [83]. No significant differences were noted for soil N concentrations between Authorities nor by year. Median total soil N concentrations are reported at the land use by soil order level (Table S15) but used in the calculation of eroded soil N by land use as freely accessible data for soil order was unavailable at the time. The resulting estimates of seasonal soil N losses via erosion are given in Table S17 by land use (and management) and slope class. Seasonal losses are split evenly across the three months of the season: for example, if a value of 1 kg ha^-1^ is given for spring, the months of September, October and November are each allocated a soil erosion N source of a third of the kg of N-loss per hectare. Note that the risk of N loss from unproductive land is treated the same as forested land – where no N inputs are recorded and the risk of loss is limited to erosion.

**Table S16**. Mean, standard deviation, median and count of soil samples used to calculate soil total nitrogen concentrations for different land use by soil order combinations.

| **Land use by soil order** | **Mean soil total N concentration (g kg^-1^)** | **Standard deviation of soil total N (g kg^-1^)** | **Median soil total N concentration (g kg^-1^)** | **Count** |
| --- | --- | --- | --- | --- |
| Crop_Hort | 4.91 | 6.14 | 3.50 | 311 |
| Allophanic | 10.43 | 12.26 | 6.57 | 57 |
| Brown | 3.91 | 1.61 | 3.30 | 32 |
| Gley | 3.58 | 1.93 | 3.19 | 41 |
| Granular | 3.10 | 1.24 | 2.85 | 31 |
| Organic | 10.22 | 4.47 | 9.47 | 10 |
| Pallic | 3.21 | 0.87 | 2.96 | 35 |
| Pumice | 5.32 | 1.18 | 5.60 | 5 |
| Recent | 3.18 | 1.42 | 3.07 | 87 |
| Ultic | 3.50 | 0.63 | 3.71 | 13 |
| Dairy | 10.34 | 14.29 | 6.18 | 340 |
| Allophanic | 13.37 | 15.17 | 8.29 | 49 |
| Brown | 11.80 | 17.08 | 5.78 | 72 |
| Gley | 5.94 | 1.64 | 5.79 | 43 |
| Granular | 26.72 | 28.88 | 7.33 | 23 |
| Melanic | 6.60 | - | 6.60 | 1 |
| Organic | 15.23 | 6.21 | 14.28 | 16 |
| Pallic | 4.27 | 1.39 | 3.80 | 14 |
| Podzol | 5.62 | 1.28 | 5.49 | 4 |
| Pumice | 5.96 | 1.93 | 6.06 | 49 |
| Recent | 4.71 | 1.29 | 4.78 | 52 |
| Ultic | 15.90 | 20.60 | 6.85 | 17 |
| Drystock | 7.01 | 8.80 | 5.00 | 407 |
| Allophanic | 11.86 | 13.38 | 9.07 | 46 |
| Brown | 7.09 | 9.71 | 4.50 | 115 |
| Gley | 4.56 | 1.50 | 4.35 | 28 |
| Granular | 9.12 | 10.09 | 6.43 | 35 |
| Melanic | 6.75 | 1.70 | 6.80 | 5 |
| Organic | 12.56 | 5.35 | 14.80 | 5 |
| Pallic | 4.35 | 1.33 | 3.90 | 62 |
| Podzol | 6.45 | 1.82 | 5.78 | 4 |
| Pumice | 5.86 | 1.48 | 5.90 | 34 |
| Recent | 3.60 | 1.12 | 3.67 | 49 |
| Ultic | 11.55 | 15.66 | 5.81 | 24 |
| Forestry | 7.21 | 10.28 | 3.20 | 120 |
| Allophanic | 11.69 | 7.24 | 11.01 | 12 |
| Brown | 7.75 | 10.42 | 3.26 | 40 |
| Gley | 6.47 | 1.51 | 6.47 | 2 |
| Granular | 30.58 | 18.67 | 35.75 | 4 |
| Pallic | 3.12 | 0.82 | 3.30 | 9 |
| Podzol | 3.68 | 0.51 | 3.90 | 3 |
| Pumice | 3.51 | 1.17 | 3.33 | 14 |
| Recent | 0.85 | 0.73 | 0.60 | 9 |
| Ultic | 6.83 | 11.60 | 2.90 | 27 |

**Table S17**. Estimates of seasonal soil N-losses via erosion (kg N ha^-1^) by land use (and management) and slope class. Note that land uses such as dairy, arable, horticultural and winter forage cropping are highly unlikely on easy or steep slopes.

| **Season** | **Slope class** | **Native forest** | **Exotic forest** | **Dairy** | **Drystock** | **Arable (incl. perennial horticulture)** | **Horticultural (Vegetables)** | **Winter forage crop (grazed)** |
| --- | --- | --- | --- | --- | --- | --- | --- | --- |
| Spring | Flat | 0.04 | 0.05 | 0.43 | 0.43 | 0.52 | 1.27 | 0.91 |
|  | Rolling | 0.02 | 0.04 | 0.65 | 0.33 | 0.34 | 1.02 | 0.73 |
|  | Easy | 0.02 | 0.04 | 0.65 | 0.43 | 0.40 | 1.57 | 0.73 |
|  | Steep | 0.12 | 0.07 | 1.09 | 0.72 | 0.42 | 1.57 | 1.09 |
| Summer | Flat | 0.02 | 0.04 | 0.33 | 0.33 | 0.48 | 1.02 | 0.73 |
|  | Rolling | 0.08 | 0.13 | 2.14 | 1.07 | 1.11 | 3.35 | 2.39 |
|  | Easy | 0.13 | 0.22 | 3.53 | 2.36 | 1.83 | 5.53 | 3.95 |
|  | Steep | 0.50 | 0.42 | 6.70 | 4.47 | 3.46 | 10.48 | 7.49 |
| Autumn | Flat | 0.02 | 0.04 | 0.33 | 0.33 | 0.57 | 1.57 | 0.73 |
|  | Rolling | 0.08 | 0.13 | 2.14 | 1.07 | 1.30 | 5.14 | 2.39 |
|  | Easy | 0.13 | 0.22 | 3.53 | 2.36 | 2.15 | 8.50 | 3.95 |
|  | Steep | 0.50 | 0.42 | 6.70 | 4.47 | 4.08 | 16.10 | 7.49 |
| Winter | Flat | 0.06 | 0.07 | 0.54 | 0.54 | 0.60 | 1.57 | 1.09 |
|  | Rolling | 0.20 | 0.23 | 3.57 | 1.78 | 1.38 | 5.14 | 3.59 |
|  | Easy | 0.33 | 0.38 | 5.89 | 3.93 | 2.28 | 8.50 | 5.93 |
|  | Steep | 1.25 | 0.73 | 11.16 | 7.44 | 4.33 | 16.10 | 11.24 |

# Mitigations and modifiers

This section gives the description of data filters and descriptive text for how mitigations are to be used to reduce baseline risk by altering source inputs (e.g., the user uses the data contained in other sections to alter source inputs) or baseline risk is reduced via a modifier multiplier between 0-1 (Table S18).

References are given, where possible, for the original source for the magnitude of a modification multiplier (i.e., reduction effect), but are checked against four sources who have independently collated, interpreted, and summarised ranges for some modifiers [84-87]. Note that runoff here is interpreted as surface/near-surface runoff (overland flow and throughflow), and interflow.

Modifiers are presented to the user in order of effectiveness. After selection, modifiers are applied in order of most to the least effective, reducing risk by the modified amount prior to the application of the next modification. Modifiers assume full effectiveness and good implementation.

The following internal tool data will be used to filter the modifiers or mitigations to each block:

- Enterprise type - Arable, Beef, Dairy, Deer, Forestry, Grazed Forage Crop, Horticulture Annual (incl veg), Horticulture Perennial, Outdoor Pigs, Sheep, Livestock - Other
- Flow path (leaching or runoff)
- Slope (Flat, Rolling, Easy or Steep)
- Climate (annual rainfall <800, 800-1600, and >1600 mm)^[[1]](#footnote-1)^
- Soil Composition (relevant types being silt loam texture, sandy texture, sandy or not sandy textured). The filtering will be handled by 2 specific data fields that will enable modifiers 1, 2, 3 & 4:
- Riparian Filter (value can be True or False)
- Riparian Buffer (value can be True or False)

If Riparian filter = True; soil composition is relevant for modifier 1 & 2

If Riparian Buffer = True; soil composition is relevant for modifier 3

If Riparian Buffer = False; soil composition is relevant for modifier 4

**Table S18**. Reduction efficiencies (at a block scale) for mitigation actions and modifiers, relevant to flow paths and soil (Riparian Filter/Riparian Buffer) x slope x climate combinations. Note that the description applies to the implementation of the mitigation or modifier in the right place and at the right time. If actioned via a source mitigation, advice is given on which sources to alter. Modifier values are listed as the median for studies with a range given, where available. All refers to all land uses except forestry. R = runoff and L = leaching. Values in parentheses are ranges but only given to the user for reference (i.e., not used int the calculation). Confidence intervals are given where evidence permits (e.g., 0.80 ± 0.18).

| No. | Class | Action | Description | Actioned via | Enterprise  filter | Flow path  filter | Soil - Rip  Filter | Soil - Rip  Buffer | Slope | Rainfall | Modifier (multiply by) | Ref |
| --- | --- | --- | --- | --- | --- | --- | --- | --- | --- | --- | --- | --- |
| 1. | Riparian management | Narrow riparian filter (2-5% of hillslope length) | **Medium performance:** Dense grass or other vegetation at ground level. Average filter width is 2 to 5% of hillslope length. Assumes silt loam to sandy soil texture. | **Modifier** | All | Runoff | True |  | Flat |  | 0.49 (0.18-0.90) | [88] |
| 2. | Riparian management | Wide riparian filter (>5% of hillslope length) | **High performance:** Dense grass or other vegetation at ground level. Average filter width >5% of hillslope length. Assumes silt loam to sandy soil texture. | **Modifier** | All | Runoff | True |  | Flat |  | 0.32 (0.24-0.70) | [88] |
| 3. | Riparian management | Planted riparian buffer- Coarser than sandy loam | **Medium performance:** Buffer with trees and shrubs. Installed into farms where there is a shallow confining layer (< 2m depth below surface). Assumes sandy soil texture. NB. Riparian filters cannot effectively intercept artificial drainage waters. | **Modifier** | All | Leaching |  | True |  |  | 0.45 (0.30-0.60) | [88] |
| 4. | Riparian management | Planted riparian buffer- sandy loam or finer | **High performance:** Buffer with trees and shrubs. Installed into farms where there is a shallow confining layer (< 2m depth below surface). Assumes soils are not sand texture. NB. Riparian filters cannot effectively intercept artificial drainage waters. | **Modifier** | All | Leaching |  | False |  |  | 0.25 (0.00-0.30) | [88] |
| 5. | Riparian management | Stock exclusion | Preventing direct deposition of excreta and streambank damage. Assumes 100% connectivity for red deer due to wallowing and that farms comply with current stock exclusion regulations. Remaining effect estimated for catchments with high stream density. | **Modifier** | Dairy, Deer, Sheep and Beef | Runoff |  |  |  | Na | 0.80 | [55, 89-91] |
| 6. | Edge of field | Preserve and restore natural seepage wetlands | Natural seepage wetlands at the heads and sides of streams, commonly known as seeps, flushes, valley bottom or riparian wetlands. Wetlands slow water movement through them and encourage the deposition of suspended sediment and entrained contaminants. Seepage of nitrate-rich water through organic soils promotes effective nitrate-N removal via denitrification. Assumes that catchments are approximated by a block. For leaching, reductions assume that seepage wetlands receive 20% of leached N of which 75% is removed. | **Modifier** | All | Runoff  Leaching |  |  |  | All | R=0.5, L=0.85 | [92, 93] |
| 7.. | Edge of field | Constructed wetland- Small/  North Island | Assumed wetland size is ~1**%** of catchment area and that catchments are approximated by a block. Assumed mean annual air temp >12^o^C. Excludes highly permeable soils not able to sustain a wetland. | **Modifier** | All | Runoff  Leaching |  |  | Flat, Rolling | 800-1600 mm | R=0.75, L=0.88 | [94-96] |
| 8. | Edge of field | Constructed wetland- Medium/  North Island | Assumed wetland size is ~2**%** of catchment area and that catchments are approximated by a block. Assumes mean annual air temp >12^o^C. Excludes highly permeable soils not able to sustain a wetland. | **Modifier** | All | Runoff  Leaching |  |  | Flat, Rolling | 800-1600 mm | R = 0.64, L= 0.82 | [94-96] |
| 9. | Edge of field | Constructed wetland- Large /  North Island | Assumed wetland size is ~4**%** of catchment area and that catchments are approximated by a block. Assumes mean annual air temp >12^o^C. Excludes highly permeable soils not able to sustain a wetland. | **Modifier** | All | Runoff  Leaching |  |  | Flat, Rolling | 800-1600 mm | R= 0.52, L= 0.76 | [94-96] |
| 10. | Edge of field | Constructed wetland- Small/  South Island | Assumed wetland size is ~1**%** of catchment area and that catchments are approximated by a block. Assumes Mean annual air temp 8-12^o^C. Excludes highly permeable soils not able to sustain a wetland. | **Modifier** | All | Runoff  Leaching |  |  | Flat, Rolling | 800-1600 mm | R=0.82, L=0.91 | [94-96] |
| 11. | Edge of field | Constructed wetland- Medium/  South Island | Assumed wetland size is ~2**%** of catchment area and that catchments are approximated by a block. Assumes mean annual air temp 8-12^o^C. Excludes highly permeable soils not able to sustain a wetland. | **Modifier** | All | Runoff  Leaching |  |  | Flat, Rolling | 800-1600 mm | R=0.74, L=0.87 | [94-96] |
| 12. | Edge of field | Constructed wetland- Large/  South Island | Assumed wetland size is ~4**%** of catchment area and that catchments are approximated by a block. Assumes mean annual air temp 8-12^o^C. Excludes highly permeable soils not able to sustain a wetland. | **Modifier** | All | Runoff  Leaching |  |  | Flat, Rolling | 800-1600 mm | R=0.64, L=0.82 | [94-96] |
| 13. | Edge of field | Detainment bund  on free-draining soil | An engineered structure to slow water flows and allow sedimentation and infiltration. 120 m^3^ of storage volume per ha of contributing catchment, i.e., 1.5% of catchment with a 0.8 m average pond depth. Assumes that catchments are approximated by a block. Total N reduction are estimated from reductions in sediment loss (c. 50-60% from 17-55 ha catchment). We assume 30% of total N was lost in particulate form. | **Modifier** | All | Runoff |  |  | Rolling, Easy, Steep |  | 0.50 | [97, 98] |
| 14. | Edge of field | Woodchip denitrification beds intercepting tile drains | Denitrification beds comprise basins filled with woodchips that intercept drain flow before discharge to surface waters. The wood chips provide organic carbon that fuels the microbial conversion of nitrate in water to nitrogen gas, which is released to the atmosphere. Assumes Denitrification Bed 1 m deep ~1% of catchment area. Assumes that catchments are approximated by a block. Removal range is 0.1-0.8 (mid-point) of 0.5, but we assume artificial drainage captures half of the N leached | **Modifier** | All | Leaching |  |  | Flat | 800-1600 mm | 0.75 | [87, 99-102] |
| 15. | Edge of field | Cut outs or berms to direct laneway or stockyard runoff away from waterways | Direct water off laneways, near stockyards or recently cultivated paddocks away from waterways. Implementation assumes the presence of one laneway (used daily) or stockyard per 30 ha currently discharging into a waterway i.e., the effect of N-rich excreta in runoff is diluted by runoff from the rest of the ~30 ha catchment. | **Modifier** | Dairy, Sheep and Beef | Runoff |  |  |  | Na | 0.95 | [103-105] |
| 16. | Edge of field | Stock exclusion and riparian planting | Preventing direct deposition of excreta, streambank decomposition, and some filtering of soil from runoff. Assumes 100% connectivity for red deer due to wallowing and that farms comply with current stock exclusion regulations. Remaining effect estimated for catchments with high stream density. | **Modifier** | Deer, Sheep and Beef | Runoff |  |  |  | Na | 0.50 (Deer)  0.80 (Sheep and Beef) | [55, 89-91] |
| 17. | Cropping and cultivation | Catch cropping | Typically, short rotation crops with good cool season growth and a deep rooting system that helps to mop up N that would otherwise be leached. Effectiveness is dependent on when crops are sown in relation to grazing/N loading or harvest. Catch crops generally feature in two main systems: 1) summer/early autumn (Mar) and late autumn (May) cropping (S1), and 2) following winter forage crop grazing depending on the month sown (S2). Generally, for every month that sowing is delayed in S2, the efficacy declines by 10%. | **Modifier** | All (excluding perennial horticultural) | Leaching |  |  | Flat, Rolling |  | S1: 0.50 Mar, 0.90 May    S2: 0.70, 0.80 and 0.90 in Jul, Aug, and Sep, respectively. | [106], [107, 108] |
| 18. | Cropping and cultivation | Using winter active crops | Such as an annual ryegrass, Italian ryegrass and some late maturing perennial ryegrasses grow during winter and utilising soil N when leaching is likely. Effect is highly dependent on cultivar. Data shown for cv. Tabu. | Alter source inputs (change crop type for month through Appendix II) | All (excluding perennial horticultural) | Leaching |  |  |  | Na | Na | [109-111] |
| 19. | Cropping and cultivation | Direct Drilling | Avoids soil N mineralisation (hence no benefit to perennial pasture) but prevents soil disturbance, increasing roughness and likelihood of soil loss via erosion compared to conventional tillage. Reduction in particulate N assumed to be 60% and particulate N assumed to be 50% of runoff total N. | **Modifier** | All (excluding perennial horticultural) | Runoff |  |  | Flat, Rolling |  | 0.70 | [112] |
| 20. | Cropping and cultivation | Cultivation along contours | Cultivate along contours (rather than up and down the slope) to reduce erosion and loss of particulate N in runoff. Effect is highly variable and dependent on topography with a high likelihood that runoff will converge; hence, potential decrease in particulate N losses set at 20%, with particulate N comprising 50% of total runoff N loss. | **Modifier** | All (excluding perennial horticultural) | Runoff |  |  | Flat, Rolling |  | 0.90 | [72, 81, 113, 114] |
| 21. | Cropping and cultivation | Silt traps | Use silt traps to settle out sediment from water before it enters drains | **Modifier** | All (excluding perennial horticultural) | Runoff |  |  |  | Na | 0.90 | [72, 81, 113, 114] |
| 22. | Stock management | Change animal type | Animal type influences N leaching due to inherent differences in the spread of urinary N, the major source of N loss in grazed pastures. N leaching from sheep and deer is approximately half that from beef cows at the same level of feed intake. | Alter source inputs for dung and urine by changing stock type, and age by month using Appendix I | Dairy, Deer, Sheep and Beef | Leaching |  |  |  | Na | Na | [87, 115] |
| 23. | Stock management | Change stocking rate | Changes to stocking rate can be positive or negative depending on the number and type of stock present. | Alter source inputs for dung and urine by changing stock rate by month using Appendix I | Dairy, Deer, Sheep and Beef | Leaching |  |  |  | Na | Na | [116-118] |
| 24. | Stock management | Genetic improvement | Factors that affect longevity of animal lifetime acts to reduce N in urine by 6-20%: Factors include increase lambing percentages and better fertility in cattle. Calculated via lower (and linked) methane emissions. | **Modifier** | Deer and dairy cattle, sheep | Leaching |  |  |  | Na | 0.95 | [119] |
| 25. | Stock management | Increase rate of finishing, early culling in autumn | Increase rate of finishing or culling (in autumn) to remove stock from the farm faster | Alter source inputs for dung and urine by changing stock numbers by month using Appendix I | Deer, Pork, Sheep and Beef | Leaching |  |  |  | Na | Na | [115] |
| 26. | Stock management | Prevent fence line pacing | Plant fence lines and/or use outriggers to reduce pacing behaviour and erosion | **Modifier** | Deer | Runoff |  |  |  | Na | 0.95 | [66] |
|  | Additives | Nitrification inhibitors (Dicyandiamide, DCD) | Dicyandiamide (DCD) has previously been researched but no longer sold in New Zealand. This inhibitor slows the nitrification of ammonium to nitrate, reducing N available for leaching and increasing the likelihood of ammonium or nitrate being taken up by plants | **Modifier** | Dairy, Deer, Sheep and Beef | Leaching |  |  | Flat | <1600mm | 0.69 ± 0.18 | [120, 121] |
| 27. | Additives | Diuretics | Diuretics such as table salt increase water consumption by animals and cause an increase in the spread of urinary N. | **Modifier** | Dairy, Deer, Sheep and Beef | Leaching |  |  |  | Na | 0.88 | [122] |
| 28. | Additives | Use of gibberellic acid to boost pasture growth | Increase N uptake by promoting growth, especially in urine patches, if applied within 48 hrs of grazing. | **Modifier** | Dairy | Leaching |  |  |  | Na | 0.85 | [123, 124] |
| 29. | Irrigation / drainage | Variable Rate Irrigation | Applying irrigation according to soil diversity with soil moisture sensors to vary the daily rate applied and minimise leaching. Effect assumes 80% reduction of N leaching losses for centre-pivot irrigation-induced leaching (150mm out of 600mm of total drainage) equating to a total 20% reduction. | **Modifier** | All | Leaching |  |  |  | Na | 0.80 | [125, 126] |
| 30. | Irrigation / drainage | Prevent outwash from irrigation | Prevent outwash (i.e., runoff) resulting from over irrigation, most commonly from flood irrigation. Effect is for surface runoff only which is assumed to be 20% of runoff. | **Modifier** | All (excluding perennial horticultural) | Runoff |  |  |  | Na | 0.80 | [127, 128] |
| 31. | Irrigation / drainage | Controlled drainage | Delaying drainage to allow for sedimentation and denitrification. Only suitable for cropping areas in the North Island where soil deficits are strong enough for crops to benefit from increased moisture. | **Modifier** | Arable | Leaching and Runoff |  |  | Flat | North Island only | 0.86 ± 0.14 | [129, 130] |
| 32. | Grazing practices | Strategic grazing of cropland gullies | Delaying the grazing of gullies within the catchment until as late as possible in the winter and ensuring soil damage in these areas was minimized when grazing does occur. | Delay source inputs for dung and urine by changing stock numbers and age by month using Appendix I | Dairy, Deer, Sheep and Beef | Runoff |  |  | Rolling, Easy, Steep | >800 mm | Na | [75] |
| 33. | Grazing practices | On-off grazing in autumn and winter | Grazing restricted to 12 hours per day from March to May. Stock housed in barn during July and July. All winter and spring grazed crops removed from the system. | Delay source inputs for dung and urine by changing stock numbers and age by month using Appendix I | Dairy | Leaching and runoff |  |  |  | Na | Na | [131, 132] |
| 34. | Grazing practices | Use alternative forage/crop species to decrease the total N in the diet | Lowers mineral-N return to the soil | Alter source inputs for soil mineral N by changing crop type by month using Appendix II | Dairy, Deer, Sheep and Beef | Leaching and runoff |  |  |  | Na | Na | [107, 133-135] |
| 35. | Grazing practices | Graze cows off farm in winter | Removes stock from paddocks in winter when there is a high risk of loss of excretal-N in runoff and leaching | Decrease source inputs for dung and urine by removing stock in winter months using Appendix I | Dairy | Leaching and runoff |  |  |  | Na | Na | [103] |
| 36. | Grazing practices | Cut and carry pasture management with feeding facilities | Removes stock from paddocks in winter when there is a high risk of loss of excretal-N in runoff and leaching | Decrease source inputs for dung and urine by removing stock in months where cut and carry used via Appendix I | Dairy | Leaching and runoff |  |  | Flat |  | Na | [103] |
| 37. | Grazing practices | Reticulation | Discourages drinking from streams and excretal returns by placing reticulated water away from streams. Dairy already assumed to have access to reticulated water. | **Modifier** | Deer, Sheep and Beef | Runoff |  |  |  | Na | 0.95 | [115, 136] |
| 38. | Effluent management | Greater effluent pond storage and low-rate application | Coupling pond storage that is appropriate for the region (e.g., via one of the pond storage calculators and regional rules) with low rates of effluent application (< 4 mm/hr) can decrease losses by minimising the potential for surface runoff and sub surface losses via preferential flow. | **Modifier** | Dairy | Runoff |  |  | Flat |  | 0.67 | [137-139] |
| 39. | Effluent management | Better timing of effluent application | Effluent applied outside of winter-early spring | Alter source inputs for fertiliser N applied by month using Appendix IV | Dairy | Leaching and runoff |  |  | Flat |  | Na | [137, 139] |
| 40. | Effluent management | Enhanced pond systems | Covered Anaerobic Ponds to remove and digest organic suspended solids to methane-rich biogas for energy recovery. High-Rate Algal Ponds remove N in harvested algae. This is assumed to be reapplied to land. Hence savings occur via fertiliser reductions. | Decrease source inputs for fertiliser N applied by month by the amount of N saved by recycling through pond using Appendix IV | Dairy | Leaching and runoff |  |  | Flat |  | Na | [43, 140] |
| 41. | Effluent management | Move to land application system from two pond discharge to water system | Land application of effluent only allowed if shifting from direct discharge to streams. The quantity of N saved is likely to be 90% of the N going through the effluent system (producing effluent with a N concentration of 500 mg L^-1^ for 100 cows producing 70 L cow^-1^ day^-1^ for 300 days on 100 ha). Non-effluent land (90% of farm) leaches 30 kg N ha^-1^. This action is only provided for the farm level as a pond is unlikely to have its own block. | **Modifier** | Dairy | Leaching and runoff |  |  | Flat |  | 0.875% | [43, 138, 141] |
| 42. | Effluent management | Export effluent solids to run off or cropping areas | Solids are separated from effluent pond and not applied to milking platform, reduces the amount of N needed elsewhere. Estimates of the quantity of N in solids can be obtained from Houlbrooke, Longhurst [43] but approximated here to be the equivalent of ~630 kg N (300 mg N L^-1^ for 70 L cow^-1^ day^-1^ for 300 days). | Reduce inputs of fertiliser (Appendix IV) by 630 kg N over property applied in a summer month | Dairy | Leaching |  |  | Flat |  | Na | [43, 103] |
| 43. | Feed | Total N imported from feed | Decrease the kg of N as imported feed. N concentration of feed can be sourced from <https://www.dairynz.co.nz/feed/supplements/feed-values/> | Decrease inputs of fertiliser N (Appendix IV) by the amount of N applied in purchased feed | Dairy, Deer, Sheep and Beef | Leaching and runoff |  |  |  | Na | Na | [116, 142] |
| 44. | Feed | Including plantain in the diet | Results in lower N concentration in urine than cows grazing perennial ryegrass/white clover pastures. Also thought to inhibit nitrification. Reduces N loss by 1% for every 1% of plantain in diet up to a maximum of 20% plantain. Effect assumes 15% of diet is plantain in a well-kept sward over 7 years. | **Modifier** | Dairy, Deer, Sheep and Beef | Leaching |  |  |  | Na | 0.85 ± 0.15 | [126, 143-145] |
| 45. | Feed | Grow maize on effluent block | Allows lower cost maize growth on farm with no fertiliser for at least two years after pasture. Assumes linear relationship between N fertiliser application and leaching loss [118]. | Reduce annual fertiliser inputs to nil (Appendix IV) | Dairy | Leaching and runoff |  |  |  | Na | Na | [146, 147] |
| 46. | Nitrogen Fertiliser | Reduction of N fertiliser | Reduce the rate of N fertiliser applied by month | Reduce monthly fertiliser inputs by desired amount (Appendix IV) | Dairy, Deer, Sheep and Beef | Leaching and runoff |  |  |  | Na | Na | [132, 148] |
| 47. | Nitrogen Fertiliser | Precision fertiliser application | Apply rates according to soil type. Assumes an average reduced rate is applied representative of the area-weighted soil fertiliser is 30% less for a block. | Reduce monthly fertiliser inputs (Appendix IV) by 30% (or calculated saving from user/advisor) | All | Leaching |  |  |  | Na | Na | [103] |
| 48. | Stock exclusion | Alternative wallowing | Only applies to blocks with many wallows directly connected to streams, thereby providing a direct conduit for excreta deposited and the bed sediment disturbed during wallowing. A solution sees the fencing off or existing connected wallows and the creation of a wallow that is not connected to a stream. Effect only applies to 90% reductions in sediment, and hence sediment associated ammoniacal- and particulate-N lost in runoff. Ammoniacal- and particulate-N is assumed to be 50% of total N losses. | **Modifier** | Deer | Runoff |  |  |  | Na | 0.55 | [149] |
| 49. | Stock exclusion | Bridging stock crossings of streams | Avoid direct entry of faeces, urine and entrained hoof mud, and substrate disturbance during stream crossings | **Modifier** | Deer, Sheep and Beef | Runoff |  |  |  | Na | 0.95 | [87] |
| 50. | Forestry | Increasing forested area | Forest area doubled from 12.5% to 25% (on average) with erosion prone land planted first | Alter erosion input by modifying soil erosion losses via Table 1 Appendix III. Set monthly fertiliser input (Appendix IV) to nil | Sheep, beef | Runoff |  |  | Rolling, Easy, Steep |  | Na | [86, 150-153] |
| 51. | Forestry | Tree harvest | Season of harvest. Rapid establishment of vegetation cover after harvest. Effect is relative to standard forestry practice which see nitrate-N losses increase 2-6 times pre-harvest concentrations for six months. Effect discounted for 20-year rotation | **Modifier** | Forestry | Leaching and runoff |  |  | Rolling, Easy, Steep |  | 0.95 | [151, 154, 155] |
| 52. | Forestry | Space planting of trees | To reduce sediment or faecal loads coming from small areas of high runoff | Alter erosion input by modifying soil erosion losses via Table 1 Appendix III. Set monthly fertiliser input (Appendix IV) to nil | Deer, Sheep and Beef | Leaching and runoff |  |  | Rolling, Easy, Steep |  | Na | [150, 151, 155] |

# Testing the transport and overall risk

This section outlines two aspects:

1. Sensibility testing looking at the effect of different factors on APSIM transport outputs.
2. A comparison of observations of N loss and against tool estimates of risk.

## 1) Sensibility testing – transport risk

Leaching transport risk was derived from APSIM modelling of the probable leaching of a spike of N applied in any given month leaching below the root zone within two years. Runoff risk was derived from the median amount of runoff simulated by APSIM. The process is described in the Overview document. This section documents the sensibility testing of that APSIM modelling.

### Sub-sampling of the population of locations for sensibility analysis

The full population of locations (a soil-weather combination) was over 81,000 valid combinations. This is too many to produce a meaningful sensibility analysis so sub-sampling was required.

Rainfall (which drives both leaching and runoff transport as well as growing conditions) and air temperature (which drives growth) are known important factors. Preliminary analysis of the full data set showed that there was a negative relationship (Fig. S2) between these two variables, so the first decision was to sample with respect to the distribution of rainfall and then check that the sub-sample was a good representation of the air temperature distribution.


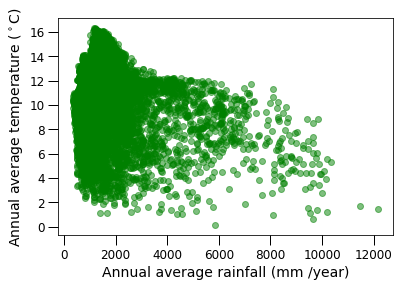


**Figure S2**. Plot of rainfall against average air temperature for the 10562 weather locations used in the simulations.

The full data set of 81710 naturally occurring combinations of weather data (henceforth termed “Agents”) and S-Map soil siblings (henceforth “Soils”) comprised the Population. The Agents were ranked (using pandas.DataFrame.rank with method=’average’) according to annual average rainfall and then all Agent-Soil combinations for every 10^th^ rank was selected as a sub-sample. This resulted in 4026 Agent-Soil combinations. The sub-sample is less than 10% of the population because of the method used for ranking in combination with the effect of the relatively wide and skewed (varying between 1 and 55 with a median of 7) distribution of soils per Agent (Fig, S3). The sampling regime did not include consideration of this feature of the population. This bias in the sampling is not important provided other key features of the climates and soils in the sub-sample are representative of the population.


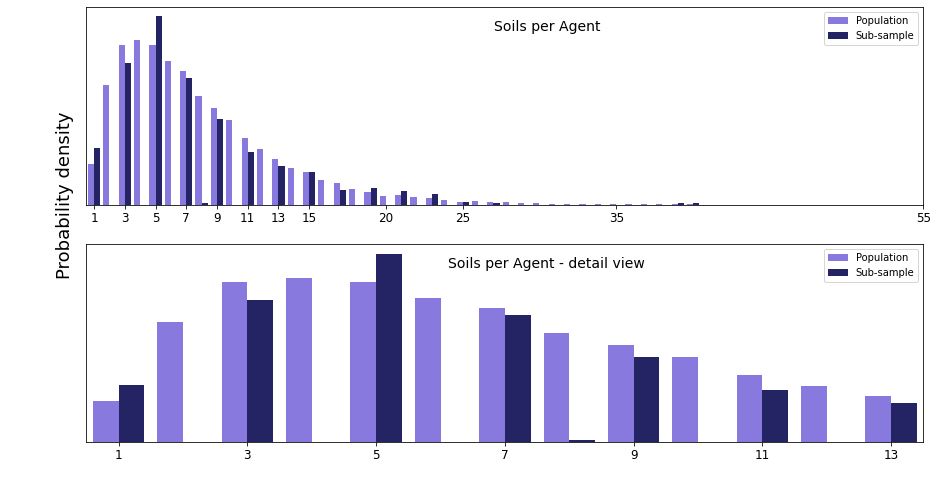


**Figure S3**. Probability density of the number of Soils within Agents for the population (lighter blue) and sub-sample (darker blue) showing the entire range (upper) and excluding the long tail (lower).

Following the above sampling, the distributions of rainfall, air temperature, plant-available water in the soil (PAW), saturated hydraulic conductivity in the topsoil (Ksat), concentration of soil carbon in the topsoil (Carbon), and soil order (Order) in the population and sub-sample were compared (Fig. S4). The distributions are favourable, so analysis proceeded with the sub-sample.


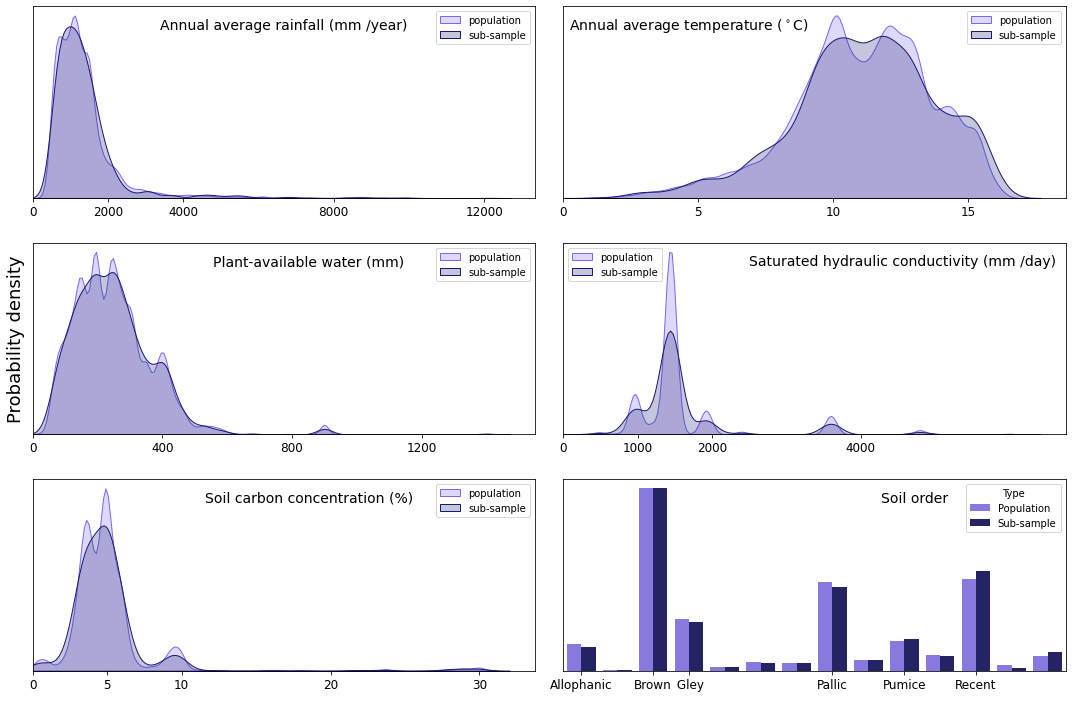


**Figure S4.** Probability densities of the sub-sample (sample size of 4026, darker blue) of Agent-Soil combinations compared to that of the full population (population 81710, lighter blue) with the characteristic concerned as shown on the individual plots.

### Calculation of transport risks

To make the runoff of water more consistent with the way we approach leaching transport factors, runoff from APSIM calculations for all land uses were divided by 200 (approximately the 98^th^ percentile of estimated runoff in mm). However, our initial estimates of the risk of N loss by runoff was far below observed N losses. We attribute this to the use of transport factors for all slope classes that were based on calculations for flat land. This meant that we were underestimating runoff from steep land. To gain equivalence between land uses we therefore multiplied all runoff values for forest (largely associated with steeper slopes) by 20 to get runoff close to the New Zealand-wide median value. We used the same adjustment for all flat land, as we had no data to warrant a different value.

Transport risk was calculated across 41 years of historic weather data so there was more than one possibility for aggregating the effect of year-to-year variability with the mean or the median value being the most sensible options. Figure S5 shows the effect of the two aggregation options against the unaggregated dataset. There was minimal variation between the two so the median was selected for usage.


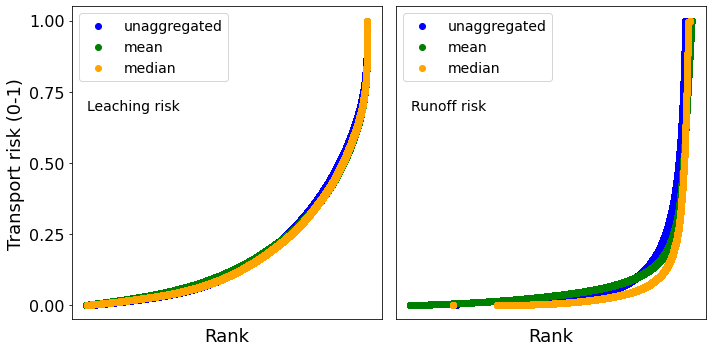


**Figure S5**. Leaching (left) and runoff (right) transport risk (vertical axis) plotted against the relative rank of the population (blue), the mean (green) and median (gold) across years of the sub-sample.

### Effect of weather and soil properties on transport risk

While the tool outputs were tested against data from the literature (see testing section) that data are relatively sparse. Therefore, extensive sensibility testing was done. Sensibility testing involved plotting the transport risk against expected drivers and examining the patterns for sensibility against expectation.

Note that, to simplify the language, risks are referred to as, for example, “in July”. More fully this means the risk of activities in July on leaching in the following two year or runoff in the following 30 days.

### Effect of weather and plant growth drivers on transport risks

Rainfall is an obvious driver of transport risk, yet its effects are not straightforward. Low rainfall can slow drainage and therefore transport but if rainfall is too low to support much growth, then risk can increase. The pattern and variability of the rainfall is also important to risk. The general patterns below (Fig. S6) make sense:

- examining the lower envelope of the data, there was a general trend for increasing risks with increasing rainfall;
- irrigation reduced leaching risk at low-rainfall sites in January (and to a much lesser extent in July), likely because of increased growth and uptake of N;
- runoff risk increased with rainfall at moderate rainfalls (those found in most agricultural areas) and was higher in July c.f. January; and
- at lower rainfall sites, irrigation increased runoff risk in January but not July.


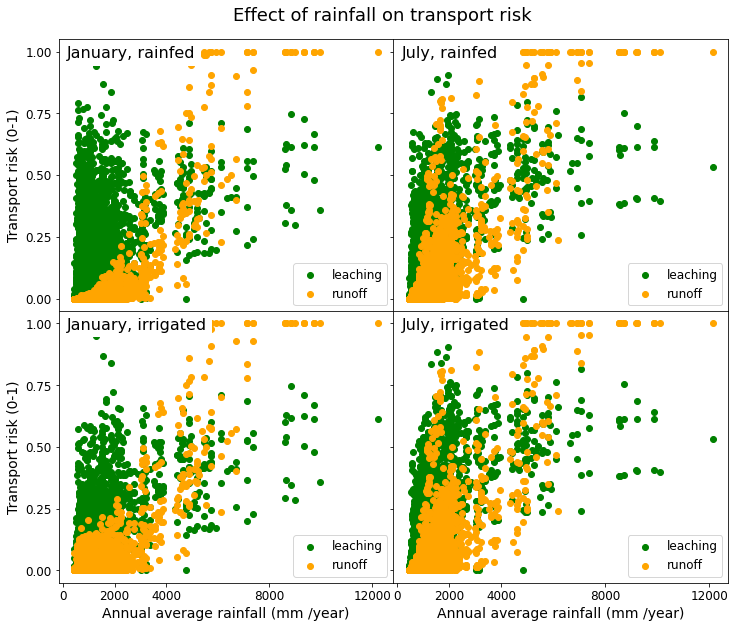


**Figure S6.** Effect of Agent annual average rainfall on leaching (green) and runoff (gold) transport risk in January (left) and June (right) for rainfed (upper) and irrigated (lower) conditions.

There were only minor patterns observable between air temperature and transport risks (Fig. S7) and most of the effects is likely through a secondary driver (plant growth) and the association between air temperature and rainfall.


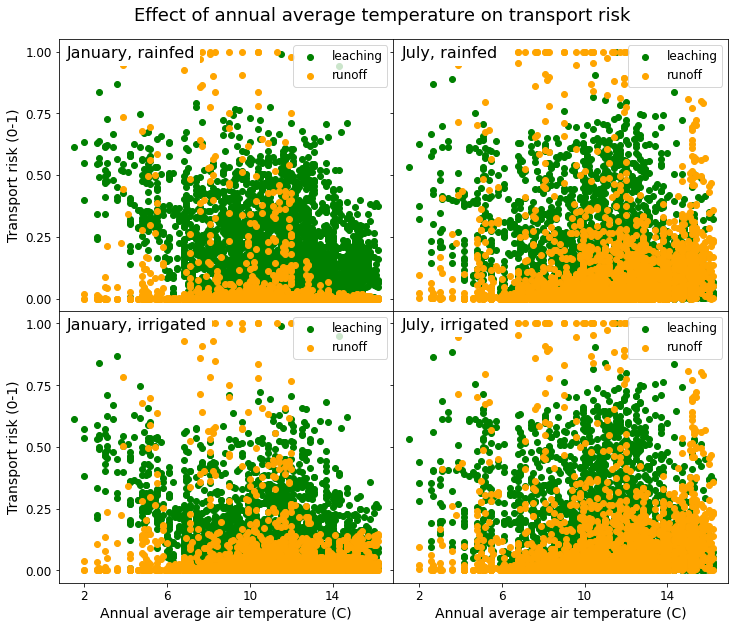


**Figure S7.** Effect of Agent annual average air temperature on leaching (green) and runoff (gold) transport risk in January (left) and June (right) for rainfed (upper) and irrigated (lower) conditions.

There was a strong negative relationship between plant production and leaching risk (Fig. S8). Plant production is an integrator of many weather and soil variables and takes account of, for example, variation of rainfall within and between years in a way that plotting against average rainfall cannot. As expected, there was little association between plant production and runoff risk.


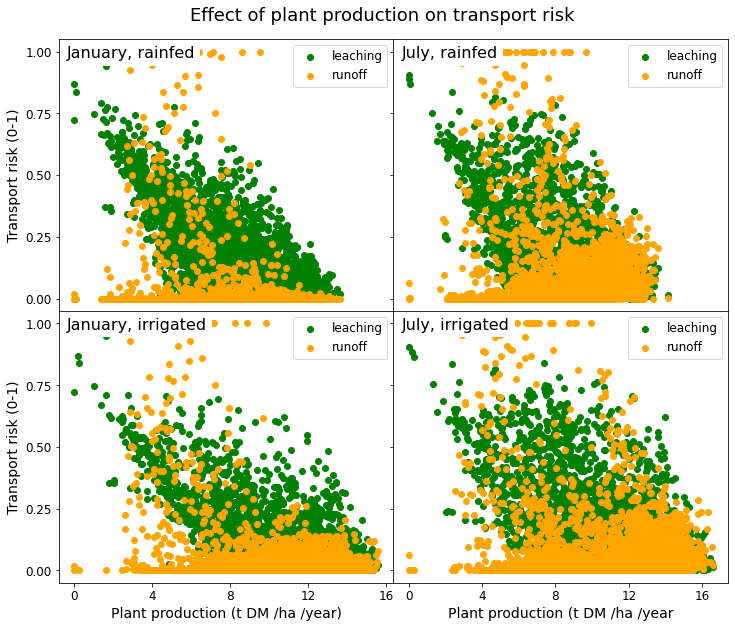


**Figure S8.** Effect of simulated plant production on leaching (green) and runoff (gold) transport risk in January (left) and June (right) for rainfed (upper) and irrigated (lower) conditions.

### Effect of soil properties on transport risks

Transport risks were examined against several soil properties. Figure S9 shows that leaching risk, as expected, generally increased as topsoil saturated hydraulic conductivity increased. The pattern of runoff risk with conductivity is somewhat messy at low conductivities (probably following the sampling distribution, see Fig. S2). At higher conductivities, runoff risk is generally low as might be expected.

Transport risks were also examined (data not shown) against the topsoil properties of carbon concentration, clay content, soil order and plant-available water within the pasture’s rootzone. No unexpected patterns were observed.


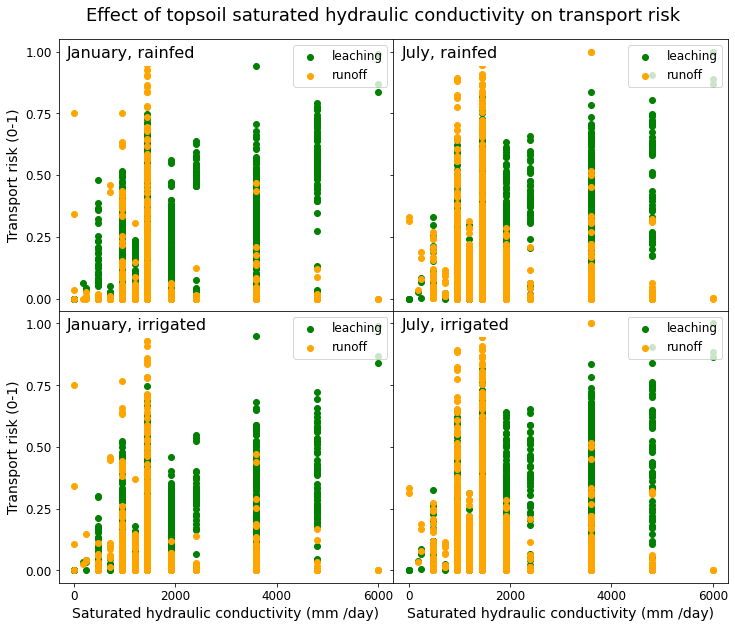


**Figure S9** Effect of topsoil saturated hydraulic conductivity on leaching (green) and runoff (gold) transport risk in January (left) and June (right) for rainfed (upper) and irrigated (lower) conditions.

## 2) Testing

### Comparing the range and relative magnitude of risk scores

We reinspected our database of observations (n = 155) by land use and flow path, separating measurements of leaching from runoff (inclusive of surface runoff and interflow calculated by difference from evapotranspiration and leaching). Using each observation’s location, modelled transport risks were multiplied by recorded N sources.

Observations were filtered out where there was low confidence in N inputs or the location, or where observations were recorded at an inappropriate scale (e.g., catchments >10ha). We also only included the mean of observations where multiple years of data were collected. Filtering resulted in 94 observations split across 1 observation for beef, 12 for cropping, 25 for dairy, 5 for deer, 7 for exotic forestry, 14 for horticulture, 5 for native forest, 5 for sheep, 11 for vegetables, and 3 for grazed winter forage cropping (Fig S10).

We plotted the risk of runoff plus leaching and runoff alone against the observations to determine if the range and relationship of estimated risk has similarity to that of observations (Figures S10-11). This plot was used to check if the magnitude of risk was within the range of observations and to check if risk responded to input values like that recorded for management at observed sites.

Once filtered, data points largely fell within the 95% prediction interval. We did not assess performance of the risk index using this relationship as the data were not normally distributed.

**Figure S10**. Plot of unfiltered (empty circles) and filtered (filled circles) observed (leaching + runoff) nitrogen losses against their corresponding risk nitrogen loss index values. The equation for the linear regression is shown to allow readers to gauge the magnitude of risk index values relative to observed values (via the slope = 0.95) but readers should be cautioned that this relationship does not hold statistical validity. The two values to the far right (145, 101) lie beyond the graph’s range and are from fluxmeter data.

**Figure S11**. Plot of filtered observed runoff (alone) nitrogen losses against their corresponding risk values.

# References

1. Luo, J., et al. *Review and revision of the methane conversion factor (MCF) for dairy cattle manure*. 2022. 34.

2. Stewart, D.P.C. and R. Rout *Reasonable Stock Water Requirements Guidelines for Resource Consent Applications*. 2007. 37.

3. Pickering, A., et al., *Methodology for calculation of New Zealand's agricultural greenhouse gas emissions*. 2022, Ministry for Pirmary Industries: Wellington, New Zealand. p. 223.

4. Hill, J., *Recalculate Pork Industry Emissions Inventory*. 2012: Palmerston North, New Zealand. p. 99.

5. Paul, E.A. and F.E. Clark, *Soil Microbiology and Biochemistry*. 1989, San Diego, CA: Academic Press Inc.

6. Trinsoutrot, I., et al., *Biochemical Quality of Crop Residues and Carbon and Nitrogen Mineralization Kinetics under Nonlimiting Nitrogen Conditions.* Soil Science Society of America Journal, 2000. **64**(3): p. 918-926.

7. Bolger, T.P., et al. *Nitrogen mineralisation from shoot and root residues of crop and pasture species*. 2001.

8. Chaves, B., et al., *Nitrogen mineralization of vegetable root residues and green manures as related to their (bio)chemical composition.* European Journal of Agronomy, 2004. **21**(2): p. 161-170.

9. Vigil, M.F. and D.E. Kissel, *Equations for Estimating the Amount of Nitrogen Mineralized from Crop Residues.* Soil Science Society of America Journal, 1991. **55**(3): p. 757-761.

10. De Neve, S. and G. Hofman, *Modelling N mineralization of vegetable crop residues during laboratory incubations.* Soil Biology and Biochemistry, 1996. **28**(10): p. 1451-1457.

11. Thomas, S., et al., *Review of nitrous oxide emission factors and activity data for crops*. 2011, Plant and Food Research: Wellington, New Zealand. p. 80.

12. Nicolardot, B., S. Recous, and B. Mary, *Simulation of C and N mineralisation during crop residue decomposition: A simple dynamic model based on the C:N ratio of the residues.* Plant and Soil, 2001. **228**(1): p. 83-103.

13. Lott, D.E. and V.E. Hammond *Water Wise: Vegetable and Fruit Production*. 2013. 4.

14. Alberta Agriculture and Forestry *Alberta Irrigation Management Manual*. 2016. 54.

15. Rahn, C.R. and R.D. Lillywhite, *A study of the quality factors affecting the short-term decomposition of field vegetable residues.* Journal of the Science of Food and Agriculture, 2002. **82**(1): p. 19-26.

16. Curtin, D., et al. *Contribution of vegetable crop residues to available nitrogen supply*. 2022. In press.

17. Carranca, C., et al., *Temporal dynamics of soil nitrogen, carbon and microbial activity in conservative and disturbed fields amended with mature white lupine and oat residues.* Geoderma, 2009. **151**(1): p. 50-59.

18. Muldoon, D., *Dry matter accumulation and changes in forage quality during primary growth and three regrowths of irrigated winter cereals.* Australian Journal of Experimental Agriculture, 1986. **26**(1): p. 87-98.

19. Kucharik, C.J. and K.R. Brye, *Integrated BIosphere Simulator (IBIS) Yield and Nitrate Loss Predictions for Wisconsin Maize Receiving Varied Amounts of Nitrogen Fertilizer.* Journal of Environmental Quality, 2003. **32**(1): p. 247-268.

20. Rezgui, C., et al., *Linking changes in the soil microbial community to C and N dynamics during crop residue decomposition.* Journal of Integrative Agriculture, 2021. **20**(11): p. 3039-3059.

21. Chatterjee, A. and U. Acharya, *Controls of carbon and nitrogen releases during crops’ residue decomposition in the Red River Valley, USA.* Archives of Agronomy and Soil Science, 2020. **66**(5): p. 614-624.

22. Thiébeau, P., et al., *Dataset of biomass and chemical quality of crop residues from European areas.* Data in Brief, 2021. **37**: p. 107227.

23. University of Minnesota Extension. *Can you take a nitrogen credit following sweet corn?* Minnesota Crop News 2021 [cited 2022; Available from: <https://blog-crop-news.extension.umn.edu/2021/08/can-you-take-nitrogen-credit-following.html>.

24. Nett, L., et al., *Emissions of nitrous oxide and ammonia after cauliflower harvest are influenced by soil type and crop residue management.* Nutrient Cycling in Agroecosystems, 2016. **106**(2): p. 217-231.

25. Kage, H. and H. Stützel, *A simple empirical model for predicting development and dry matter partitioning in cauliflower (Brassica oleracea L. botrytis).* Scientia Horticulturae, 1999. **80**(1): p. 19-38.

26. Jett, L.W., R.D. Morse, and C.R. O'Dell, *Plant Density Effects on Single-head Broccoli Production.* HortScience HortSci, 1995. **30**(1): p. 50-52.

27. Bending, G.D., M.K. Turner, and I.G. Burns, *Fate of nitrogen from crop residues as affected by biochemical quality and the microbial biomass.* Soil Biology and Biochemistry, 1998. **30**(14): p. 2055-2065.

28. Trolove, S., et al. *Protecting our groundwater: Fluxmeter network summary report*. 2021. **Report 20648**.

29. Hamilton, H.A. and R. Bernier, *N–P–K Fertilizer effects in yield, composition and residues of lettuce, celery, carrot and onion grown in an organic soil in Qubec.* Canadian Journal of Plant Science, 1975. **55**(2): p. 453-461.

30. Paterson, C.D. and C.R. Rahn, *The nitrogen contribution of lettuce crop residues intensive vegetable rotations.* Acta Horticulturae, 1996. **428**: p. 105-114.

31. Mitchell, R., J. Webb, and R. Harrison, *Crop residues can affect N leaching over at least two winters.* European Journal of Agronomy, 2001. **15**(1): p. 17-29.

32. Duarte, L.O., et al., *Dry matter and nutrient accumulation curve in cabbage crop.* Agronmia Revista Caatinga, 2019. **32**(3): p. 679-689.

33. Turan, M., et al., *Yield and Chemical Composition of Brussels Sprout (Brassica oleracea L. gemmifera) as Affected by Boron Management.* HortScience horts, 2009. **44**(1): p. 176-182.

34. Zink, F.W., *Celery growth and nutrient absorption studies.* Hilgardia, 1966. **20**(7): p. 10-10.

35. Chen, D., et al., *Influence of Lag Effect, Soil Release, And Climate Change on Watershed Anthropogenic Nitrogen Inputs and Riverine Export Dynamics.* Environmental Science & Technology, 2014. **48**(10): p. 5683-5690.

36. Thomas, S., D. Wallace, and M. Beare, *Pasture renewal activity data and factors for New Zealand*. 2014, Plant and Food Research: Wellington, New Zealand. p. 65.

37. Wheeler, D.M. *OVERSEER® Technical Manual: Technical Manual for the description of the OVERSEER® Nutrient Budgets engine*. 2018. 48.

38. Ballance Agri-Nutrients, *Ballance product price list*. 2022, Ballance Agri-Nutrients: Tauranga, New Zealand. p. 11.

39. Ravensdown Fertiliser Co-operative, *Fertiliser prices*. 2022, Ravensdown Fertiliser Co-operative: Christchurch, New Zealand. p. 9.

40. Parker, M.B., H.F. Perkins, and H.L. Fuller, *Nitrogen, Phosphorus and Potassium Content of Poultry Manure and Some Factors Influencing Its Composition1.* Poultry Science, 1959. **38**(5): p. 1154-1158.

41. Defra, *The Fertiliser Manual*. 2010, Government Publications: UK.

42. Sims, J.T. and D.C. Wolf, *Poultry Waste Management: Agricultural and Environmental Issues*, in *Advances in Agronomy*, D.L. Sparks, Editor. 1994, Academic Press. p. 1-83.

43. Houlbrooke, D., et al., *Characterising dairy manures and slurries*. 2011, AgResearch: Mosgiel, New Zealand. p. 119.

44. BioRich. *BioRich Conventional Compost Analysis*. 2022 [cited 2022; Available from: <https://irp.cdn-website.com/788219e7/files/uploaded/BioRich%20Conventional%20Compost%20May%202021.pdf>.

45. McDowell, R.W. and D.J. Houlbrooke, *Management options to decrease phosphorus and sediment losses from irrigated cropland grazed by cattle and sheep.* Soil Use and Management, 2009. **25**(3): p. 224-233.

46. Muller, K., et al., *Herbicide runoff studies in an arable soil under simulated rainfall.* New Zealand Plant Protection, 2002. **55**: p. 172-176.

47. Worrall, F., T.P. Burt, and N.J.K. Howden, *The flux of suspended sediment from the UK 1974 to 2010.* Journal of Hydrology, 2013. **504**: p. 29-39.

48. McDowell, R.W., *Phosphorus and sediment loss in a catchment with winter forage grazing of cropland by dairy cattle.* J Environ Qual, 2006. **35**(2): p. 575-83.

49. Wilcock, R.J., et al., *Water quality of a lowland stream in a New Zealand dairy farming catchment.* New Zealand Journal of Marine and Freshwater Research, 1999. **33**(4): p. 683-696.

50. Monaghan, R.M., et al., *Linkages between land management activities and water quality in an intensively farmed catchment in southern New Zealand.* Agriculture, Ecosystems &amp; Environment, 2007. **118**(1–4): p. 211-222.

51. Wilcock, R.J., et al., *Land‐use impacts and water quality targets in the intensive dairying catchment of the Toenepi Stream, New Zealand.* New Zealand Journal of Marine and Freshwater Research, 2006. **40**(1): p. 123-140.

52. Wilcock, R.J., et al., *Land-water interactions in five contrasting dairying catchments: issues and solutions.* Land Use and Water Resources Research, 2007. **7**: p. 2.1-2.10.

53. Davies-Colley, R.J. and J.W. Nagels, *Effects of dairying on water quality of lowland streams in Westland and Waikato.* Proceedings of the New Zealand Grassland Association, 2002. **64**: p. 107-114.

54. McDowell, R.W., *Water quality in headwater catchments with deer wallows.* J Environ Qual, 2007. **36**(5): p. 1377-82.

55. McDowell, R.W., *Water quality of a stream recently fenced‐off from deer.* New Zealand Journal of Agricultural Research, 2008. **51**(3): p. 291-298.

56. McDowell, R.W., *Contaminant Losses in Overland Flow from Cattle, Deer and Sheep Dung.* Water, Air, and Soil Pollution, 2006. **174**(1-4): p. 211-222.

57. McDowell, R.W., *Maintaining good water and soil quality in catchments containing deer farms.* International Journal of River Basin Management, 2009. **7**(3): p. 187-195.

58. Fahey, B.D. and M. Marden, *Sediment yields from a forested and a pasture catchment, coastal Hawke's Bay, North Island, New Zealand.* Journal of Hydrology (NZ), 2000. **39**(1): p. 49-63.

59. Dons, A., *Hydrology and sediment regime of a pasture, native forest, and pine forest catchment in the Central North Island, New Zealand.* New Zealand Journal of Forestry Science, 1987. **17**(2/3): p. 161-178.

60. Quinn, J.M. and M.J. Stroud, *Water quality and sediment and nutrient export from New Zealand hill-land catchments of contrasting land use.* New Zealand Journal of Marine and Freshwater Research, 2002. **36**(2): p. 409-429.

61. Cooper, A.B. and C.E. Thomsen, *Nitrogen and phosphorus in streamwaters from adjacent pasture, pine, and native forest catchments.* New Zealand Journal of Marine and Freshwater Research, 1988. **22**: p. 279-291.

62. Hughes, A.O., J.M. Quinn, and L.A. McKergow, *Land use influences on suspended sediment yields and event sediment dynamics within two headwater catchments, Waikato, New Zealand.* New Zealand Journal of Marine and Freshwater Research, 2012. **46**(3): p. 315-333.

63. O'Loughlin, C.L., L.K. Rowe, and A.J. Rearce, *Sediment yields from small forested catchments North wetland - Nelson, New Zealand.* Journal of Hydrology (NZ), 1978. **17**(1): p. 1-15.

64. Cooke, J.G. and T. Dons, *Source and sinks of nutrients in a New Zealand hill pasture catchment I. Stormflow generation.* Hydrological Processes, 1988. **2**: p. 109-122.

65. Lambert, M.G., et al., *Losses of nitrogen, phosphorus, and sediment in runoff from hill country under different fertiliser and grazing management regimes.* New Zealand Journal of Agricultural Research, 1985. **28**: p. 371-379.

66. McDowell, R.W., J.J. Drewry, and R.J. Paton, *Effects of deer grazing and fence-line pacing on water and soil quality.* Soil Use and Management, 2004. **20**(3): p. 302-307.

67. Smith, C.M., *Sediment, phosphorus, and nitrogen in channelised surface run-off from a New Zealand pastoral catchment.* New Zealand Journal of Marine and Freshwater Research, 1987. **21**(4): p. 627-639.

68. Bargh, B.J., *Output of water, suspended sediment, and phosphorus and nitrogen forms from a small agricultural catchment.* New Zealand Journal of Agricultural Research, 1978. **21**(1): p. 29-38.

69. Williamson, R.B., C.M. Smith, and A.B. Cooper, *Watershed riparian management and its benefits to a eutrophic lake.* Journal of Water Resources Planning and Management, 1996. **122**: p. 24-32.

70. Thorrold, B.S., et al., *Oteramika catchment study*, in *Proceedings of the New Zealand Fertiliser Manufacturers' Research Association Inc. Conference*. 1997, New Zealand Fertiliser Manufacturers' Research Association Inc, Auckland, New Zealand: Invercargill, New Zealand. p. 119-128.

71. Basher, L.R., C.W. Ross, and J. Dando, *Effects of carrot growing on volcanic ash soils in the Ohakune area, New Zealand.* Soil Research, 2004. **42**(3): p. 259-272.

72. Basher, L.R. and C.W. Ross, *Soil erosion rates under intensive vegetable production on clay loam, strongly structured soils at Pukekohe, New Zealand.* Soil Research, 2002. **40**(6): p. 947-961.

73. Hicks, D.M., *Storm Sediment Yields from Basins with Various Landscapes in Auckland Area*. 1994, NIWA: Christchurch, New Zealand. p. 39.

74. McDowell, R.W. and D.R. Stevens, *Potential waterway contamination associated with wintering deer on pastures and forage crops.* New Zealand Journal of Agricultural Research, 2008. **51**(3): p. 287-290.

75. Monaghan, R.M., et al., *Grazing strategies for reducing contaminant losses to water from forage crop fields grazed by cattle during winter.* New Zealand Journal of Agricultural Research, 2017. **60**(3): p. 333-348.

76. Burkitt, L.L., J.L. Winters, and D.J. Horne, *Sediment and nutrient losses under winter cropping on two Manawatu hill country soils.* Proceedings of the New Zealand Grassland Association, 2017. **79**: p. 19-26.

77. Ministry for the Environment *National Policy Statement for Freshwater Management 2020*. 2020. 70.

78. Donovan, M., *Modelling soil loss from surface erosion at high-resolution to better understand sources and drivers across land uses and catchments; a national-scale assessment of Aotearoa, New Zealand.* Environmental Modelling & Software, 2022. **147**: p. 105228.

79. Bakker, M.M., et al., *The response of soil erosion and sediment export to land-use change in four areas of Europe: The importance of landscape pattern.* Geomorphology, 2008. **98**(3): p. 213-226.

80. Nendel, C., D. Melzer, and P.J. Thorburn, *The nitrogen nutrition potential of arable soils.* Scientific Reports, 2019. **9**(1): p. 5851.

81. Dymond, J.R., *Soil erosion in New Zealand is a net sink of CO2.* Earth Surface Processes and Landforms, 2010. **35**(15): p. 1763-1772.

82. Dymond, J.R., H.D. Betts, and C.S. Schierlitz, *An erosion model for evaluating regional land-use scenarios.* Environmental Modelling & Software, 2010. **25**(3): p. 289-298.

83. NZStats. *Soil quality and land use*. 2022 [cited 2022 20 May]; Available from: <https://www.stats.govt.nz/indicators/soil-quality-and-land-use>.

84. Matheson, L., U. Djanibekov, and S. Greenhalgh, *Recommended mitigation bundles for cost analysis of mitigation of sediment and other freshwater contaminants in the Rangitāiki and Kaituna-Pongakawa-Waitahanui water management areas*. 2018, PerrinAg: Rotorua, New Zealand. p. 45.

85. Edkins, R., et al., *Reducing N-loss to water: A summary of available and effective solutions*. 2022, Lumen Environmental: Christchurch, New Zealand. p. 51.

86. McDowell, R.W., et al., *Quantifying contaminant losses to water from pastoral land uses in New Zealand III. What could be achieved by 2035?* New Zealand Journal of Agricultural Research, 2021.

87. McDowell, R.W., R.J. Wilcock, and D. Hamilton, *Assessment of Strategies to Mitigate the Impact or Loss of Contaminants from Agricultural Land to Fresh Waters*. 2013, Ministry for the Environment: Wellington, New Zealand.

88. McKergow, L., et al., *Preliminary riparian buffer guidelines: Filtering surface runoff and nitrate removal from subsurface flow*. 2020, NIWA: Hamilton, New Zealand. p. 41.

89. Daigneault, A.J., F.V. Eppink, and W.G. Lee, *A national riparian restoration programme in New Zealand: Is it value for money?* Journal of Environmental Management, 2017. **187**: p. 166-177.

90. O’Callaghan, P., et al., *The Environmental Impact of Cattle Access to Watercourses: A Review.* Journal of Environmental Quality, 2019. **48**(2): p. 340-351.

91. Low, H., I. McNab, and J. Brennan, *Mitigating nutrient loss from pastoral and crop farms* 2017, Horizons Regional Council: Palmerston North, New Zealand. p. 39.

92. Rutherford, J.C., D. Schroer, and G. Timpany, *How much runoff do riparian wetlands affect?* New Zealand Journal of Marine and Freshwater Research, 2009. **43**(5): p. 1079-1094.

93. McKergow, L., A. Hughes, and K. Rutherford, *Seepage wetland protection review*. 2017, NIWA: Hamilton, New Zealand. p. 46.

94. Tanner, C.C., et al., *Constructed Wetland Practitioners Guide: Design and Performance Estimates*. 2022, DairyNZ and NIWA: Hamilton, New Zealand. p. 40.

95. Tanner, C.C. and R.H. Kadlec, *Influence of hydrological regime on wetland attenuation of diffuse agricultural nitrate losses.* Ecological Engineering, 2013. **56**: p. 79-88.

96. Tanner, C.C. and J.P.S. Sukias, *Multiyear Nutrient Removal Performance of Three Constructed Wetlands Intercepting Tile Drain Flows from Grazed Pastures.* Journal of Environmental Quality, 2011. **40**(2): p. 620-633.

97. Levine, B., et al., *The ability of detainment bunds to decrease sediments transported from pastoral catchments in surface runoff.* Hydrological Processes, 2021. **35**(8): p. e14309.

98. Levine, B., *The ability of detainment bunds to mitigate the impact of pastoral agriculture on surface water quality in the Lake Rotorua catchment : a thesis presented in partial fulfilment of the requirements for the degree of Doctor of Philosophy in Soil Science, Palmerston North, New Zealand*. 2020, Massey University.

99. Schipper, L.A., et al., *Denitrifying bioreactors—An approach for reducing nitrate loads to receiving waters.* Ecological Engineering, 2010. **36**(11): p. 1532-1543.

100. Rivas, A., et al., *Nitrate removal and secondary effects of a woodchip bioreactor for the treatment of subsurface drainage with dynamic flows under pastoral agriculture.* Ecological Engineering, 2020. **148**: p. 105786.

101. Maxwell, B.M., et al., *High-frequency, in situ sampling of field woodchip bioreactors reveals sources of sampling error and hydraulic inefficiencies.* Journal of Environmental Management, 2020. **272**: p. 110996.

102. Hudson, N., S. Heubeck, and E. Baddock, *Woodchip denitrification filterperformance evaluation: Third year of operation*. 2019, NIWA: Hamilton, New Zealand. p. 144.

103. Waikato Regional Council. *Menu of practices to improve water quality*. [Webpage] 2017 [cited 2017 25 April]; Available from: <http://www.farmmenus.org.nz/>.

104. Monaghan, R.M. and L.C. Smith, *Contaminant losses in overland flow from dairy farm laneways in southern New Zealand.* Agriculture, Ecosystems & Environment, 2012. **159**: p. 170-175.

105. McDowell, R.W., K. Daly, and O. Fenton, *Mitigation of phosphorus, sediment and Escherichia coli losses in runoff from a dairy farm roadway.* Irish Journal of Agricultural and Food Research, 2020. **59**(1): p. 201-205.

106. Horrocks, A., et al., *Catch Crops for Reduced Nitrate Leaching: Lessons from the “Forages for Reduced Nitrate Leaching” programme and Sustainable Food and Fibre Futures project “Catch Crops to Reduce Nitrate Leaching”.* 2021: Christchurch, New Zealand. p. 17.

107. Malcolm, B.J., et al., *Catch crops and feeding strategy can reduce the risk of nitrogen leaching in late lactation fodder beet systems.* New Zealand Journal of Agricultural Research, 2020. **63**(1): p. 44-64.

108. Malcolm, B.J., et al., *Oat catch crop efficacy on nitrogen leaching varies after forage crop grazing.* Nutrient Cycling in Agroecosystems, 2022. **122**(3): p. 273-288.

109. Malcolm, B.J., et al., *The effect of four different pasture species compositions on nitrate leaching losses under high N loading.* Soil Use and Management, 2014. **30**(1): p. 58-68.

110. Carey, P.L., et al., *Comparison of nitrate leaching from oats and Italian ryegrass catch crops following simulated winter forage grazing: a field lysimeter study.* New Zealand Journal of Agricultural Research, 2017. **60**(3): p. 298-318.

111. Maxwell, T.M.R., et al., *Italian ryegrass swards reduce N leaching via greater N uptake and lower drainage over perennial ryegrass cultivars varying in cool season growth rates.* New Zealand Journal of Agricultural Research, 2019. **62**(1): p. 69-82.

112. Daigneault, A. and A.H. Elliott, *Land-use contaminant loads and mitigaiton costs*. 2017, Motu Economic and Public Policy: Wellington, New Zealand.

113. Horticulture New Zealand, *An overview: Horticulture industry strategy 'Growing a new future'*. 2010, Horticulture New Zealand: Wellington. p. 4.

114. Basher, L., et al., *Erosion and sediment transport from the market gardening lands at Pukekohe, Auckland, New Zealand.* Journal of hydrology. New Zealand, 1997. **36**(1): p. 73-95.

115. Doole, G.J., *Description of mitigation options defined within the economic model for Healthy Rivers Wai Ora Project*. 2015, University of Waikato: Hamilton, New Zealand. p. 77.

116. Beukes, P.C., et al., *The relationship between milk production and farm-gate nitrogen surplus for the Waikato region, New Zealand.* Journal of Environmental Management, 2012. **93**(1): p. 44-51.

117. Gourley, C.J.P. and D.M. Weaver, *Nutrient surpluses in Australian grazing systems: management practices, policy approaches, and difficult choices to improve water quality.* Crop and Pasture Science, 2012. **63**(9): p. 805-818.

118. Silva, R.G., et al., *A lysimeter study of the impact of cow urine, dairy shed euent, and nitrogen fertiliser on nitrate leaching.* Soil Research, 1999. **37**(2): p. 357-370.

119. Cruickshank, G.J., B.C. Thomson, and P.D. Muir, *Effect of management change on methane output within a sheep flock.* Proceedings of the New Zealand Society of Animal Production, 2009. **69**: p. 170-173.

120. Cameron, K.C., H.J. Di, and J.L. Moir, *Dicyandiamide (DCD) effect on nitrous oxide emissions, nitrate leaching and pasture yield in Canterbury, New Zealand.* New Zealand Journal of Agricultural Research, 2014. **57**(4): p. 251-270.

121. Ledgard, S.F., et al., *Effects of the nitrification inhibitor dicyandiamide (DCD) on pasture production, nitrous oxide emissions and nitrate leaching in Waikato, New Zealand.* New Zealand Journal of Agricultural Research, 2014. **57**(4): p. 294-315.

122. Ledgard, S.F., B. Welten, and K. Betteridge, *Salt as a mitigation option for decreasing nitrogen leaching losses from grazed pastures.* Journal of the Science of Food and Agriculture, 2015. **95**(15): p. 3033-3040.

123. Woods, R.R., et al., *Effects of forage type and gibberellic acid on nitrate leaching losses.* Soil Use and Management, 2016. **32**(4): p. 565-572.

124. Bishop, P. and P. Jeyakumar, *A comparison of three nitrate leaching mitigation treatments with dicyandiamide using lysimeters.* New Zealand Journal of Agricultural Research, 2021: p. 1-14.

125. McDowell, R.W., *Does variable rate irrigation decrease nutrient leaching losses from grazed dairy farming?* Soil Use and Management, 2017. **33**(4): p. 530-537.

126. Carlton, A.J., et al., *Nitrate leaching losses are lower from ryegrass/white clover forages containing plantain than from ryegrass/white clover forages under different irrigation.* New Zealand Journal of Agricultural Research, 2019. **62**(2): p. 150-172.

127. Houlbrooke, D., P. Carey, and R. Williams, *Management practices to minimise wipe-off losses from border-dyke irrigated land*, in *Carbon and nutrient management in agriculture*, L.D. Currie and L.J. Yates, Editors. 2008, Fertilizer and Lime Research Centre, Massey University: Palmerston North, New Zealand.

128. Monaghan, R.M., et al., *Linkages between land management activities and stream water quality in a border dyke-irrigated pastoral catchment.* Agriculture, Ecosystems & Environment, 2009. **129**(1-3): p. 201-211.

129. Ballantine, D.J. and C.C. Tanner, *Controlled drainage systems to reduce contaminant losses and optimize productivity from New Zealand pastoral systems.* New Zealand Journal of Agricultural Research, 2013. **56**(2): p. 171-185.

130. McDowell, R.W., C. Gongol, and B. Woodward, *Potential for controlled drainage to decrease nitrogen and phosphorus losses to Waituna Lagoon*. 2012, AgResearch: Mosgiel, New Zealand. p. 19.

131. Christensen, C.L., et al., *Duration-controlled grazing of dairy cows. 2: nitrogen losses in sub-surface drainage water and surface runoff.* New Zealand Journal of Agricultural Research, 2019. **62**(1): p. 48-68.

132. De Klein, C.A.M., et al., *Nitrogen performance indicators for dairy production systems.* Soil Research, 2017. **55**(5-6): p. 479-488.

133. Smith, L.C. and R.M. Monaghan, *Nitrogen leaching losses from fodder beet and kale crops grazed by dairy cows in southern Southland.* Journal of New Zealand Grasslands, 2020. **82**: p. 61-71.

134. de Ruiter, J.M., et al., *Crop management effects on supplementary feed quality and crop options for dairy feeding to reduce nitrate leaching.* New Zealand Journal of Agricultural Research, 2019. **62**(3): p. 369-398.

135. Bryant, R.H., et al., *Can alternative forages substantially reduce N leaching? findings from a review and associated modelling.* New Zealand Journal of Agricultural Research, 2020. **63**(1): p. 3-28.

136. Journeaux, P. and E. van Reenen, *Economic evaluation of stock water reticulation on hill country*. 2016, AgFirst: Wellington, New Zealand. p. 56.

137. Houlbrooke, D.J., et al., *Land application of farm dairy effluent to a mole and pipe drained soil: implications for nutrient enrichment of winter-spring drainage.* Australian Journal of Soil Research, 2008. **46**: p. 45-52.

138. Houlbrooke, D.J., et al., *A review of literature on the land treatment of farm‐dairy effluent in New Zealand and its impact on water quality.* New Zealand Journal of Agricultural Research, 2004. **47**(4): p. 499-511.

139. Monaghan, R.M., D.J. Houlbrooke, and L.C. Smith, *The use of low-rate sprinkler application systems for applying farm dairy effluent to land to reduce contaminant transfers.* New Zealand Journal of Agricultural Research, 2010. **53**(4): p. 389-402.

140. Craggs, R., et al., *High rate algal pond systems for low-energy wastewater treatment, nutrient recovery and energy production.* New Zealand Journal of Botany, 2014. **52**(1): p. 60-73.

141. Wilcock, R.J., et al., *Trends in water quality of five dairy farming streams in response to adoption of best practice and benefits of long-term monitoring at the catchment scale.* Marine and Freshwater Research, 2013. **64**(5): p. 401-412.

142. Monaghan, R.M., C.A.M. de Klein, and R.W. Muirhead, *Prioritisation of farm scale remediation efforts for reducing losses of nutrients and faecal indicator organisms to waterways: A case study of New Zealand dairy farming.* Journal of Environmental Management, 2008. **87**(4): p. 609-622.

143. Al-Marashdeh, O., et al., *Integrating plantain (Plantago lanceolate l.) and italian ryegrass (lolium multiflorum lam.) into new zealand grazing dairy system: The effect on farm productivity, profitability, and nitrogen losses.* Animals, 2021. **11**(2): p. 1-20.

144. Simon, P.L., et al., *The efficacy of Plantago lanceolata for mitigating nitrous oxide emissions from cattle urine patches.* Science of The Total Environment, 2019. **691**: p. 430-441.

145. Dodd, M., et al., *A comparison of temperate pasture species mixtures selected to increase dairy cow production and reduce urinary nitrogen excretion.* New Zealand Journal of Agricultural Research, 2019. **62**(4): p. 504-527.

146. Burggraaf, V.T., et al., *A case study of the effects of diet and winter management on dairy production, profit and nitrate leaching in Rotorua.* Proceedings of the New Zealand Society of Animal Production, 2019. **79**: p. 71-73.

147. Johnstone, P., et al., *Growing maize silage in dairy effluent paddocks for two consecutive seasons - effect on crop yield and soil nitrogen.* Proceedings of the New Zealand Grassland Association, 2010. **72**: p. 117-120.

148. Ledgard, S.F., J.W. Penno, and M.S. Sprosen, *Nitrogen inputs and losses from clover/grass pastures grazed by dairy cows, as affected by nitrogen fertilizer application.* The Journal of Agricultural Science, 1999. **132**(2): p. 215-225.

149. McDowell, R.W., *The use of safe wallows to improve water quality in deer farmed catchments.* New Zealand Journal of Agricultural Research, 2009. **52**(1): p. 81-90.

150. Davis, M., *Nitrogen leaching losses from forests in New Zealand.* New Zealand Journal of Forestry Science, 2014. **44**(1): p. 2.

151. Larned, S.T., et al., *Evidence for the effects of land use on freshwater ecosystems in New Zealand.* New Zealand Journal of Marine and Freshwater Research, 2020. **54**(3): p. 551-591.

152. Monaghan, R., et al., *Quantifying contaminant losses to water from pastoral landuses in New Zealand II. The effects of some farm mitigation actions over the past two decades.* New Zealand Journal of Agricultural Research, 2021. **10.1080/00288233.2021.1876741**.

153. Dymond, J.R., et al., *Development of a New Zealand SedNet model for assessment of catchment-wide soil-conservation works.* Geomorphology, 2016. **257**(Supplement C): p. 85-93.

154. Hughes, A.O. and J.M. Quinn, *The effect of forestry management activities on stream water quality within a headwater plantation Pinus radiata forest.* Forest Ecology and Management, 2019. **439**: p. 41-54.

155. Baillie, B.R. and D.G. Neary, *Water quality in New Zealand’s planted forests: a review.* New Zealand Journal of Forestry Science, 2015. **45**(1): p. 7.

156. Drewry, J.J., et al., *Collation of nutrient, sediment, and E. coli losses from land uses to freshwater, and an initial analysis of some factors contributing to nitrogen loss*. 2022, Manaaki Whenua Landcare Research: Palmerston North, New Zealand. p. 55.

1. Climate being precipitation [↑](#footnote-ref-1)
